# On real-time calibrated prediction for complex model-based decision support in pandemics: Part 2

**DOI:** 10.1371/journal.pcbi.1014299

**Published:** 2026-06-01

**Authors:** Trevelyan J. McKinley, Daniel B. Williamson, Xiaoyu Xiong, James M. Salter, Robert Challen, Leon Danon, Ben Youngman, Doug McNeall

**Affiliations:** 1 University of Exeter Medical School, University of Exeter, Exeter, United Kingdom; 2 Department of Mathematics and Statistics, University of Exeter, Exeter, United Kingdom; 3 School of Engineering Mathematics and Technology, University of Bristol, Bristol, United Kingdom; 4 Jean Golding Institute, University of Bristol, Bristol, United Kingdom; 5 Hadley Centre for Climate Science and Services, UK Met Office, Exeter, United Kingdom; Northeastern University, UNITED STATES OF AMERICA

## Abstract

Calibration of complex stochastic infectious disease models is challenging. These often have high-dimensional input and output spaces, with the models exhibiting complex, non-linear dynamics. Coupled with a paucity of necessary data, this results in a large number of non-ignorable hidden states that must be handled by the inference routine. Likelihood-based approaches to this missing data problem are very flexible, but challenging to scale, due to having to monitor and update these hidden states. Methods based on simulating the hidden states directly from the model-of-interest have an advantage that they are often more straightforward to code, and thus are easier to implement and adapt in real-time. However, these often require evaluating very large numbers of simulations, rendering them infeasible for many large-scale problems. We present a framework for using emulation-based methods to calibrate a large-scale, stochastic, age-structured, spatial meta-population model of COVID-19 transmission in England and Wales. By embedding a model discrepancy process into the simulation model, and combining this with particle filtering, we show that it is possible to calibrate complex models to high-dimensional data by emulating the log-likelihood surface instead of individual data points. The use of embedded model discrepancy also helps to alleviate other key challenges, such as the introduction of infection across space and time. We conclude with a discussion of major challenges remaining and key areas for future work.

## Author summary

Mathematical models of infectious disease dynamics are important tools in furthering our understanding of key drivers of epidemic spread, but also in informing the efficacy of potential disease management strategies. During ongoing outbreaks, it is often necessary to develop and fit simulation models to emerging data in real-time, as new information and data emerges. This requires undesirable trade-offs, where increasing model complexity can make the outputs more realistic and useful to policy-makers, but the inference problem far more challenging (and often computationally infeasible within realistic time-frames). In Part 1 we developed a framework for using emulation-based methods (employing fast *surrogate* models) to calibrate complex epidemic models that overcomes key challenges for real-time development and fitting of complex models using uncertainty quantification methods. In this paper we further develop these approaches and show that they can be scaled to perform well in high-dimensional models, illustrating their utility by successfully calibrating a large-scale, stochastic, age-structured, spatial meta-population model of COVID-19 transmission to data available from 315 spatial regions in England and Wales. We conclude with a discussion about remaining challenges and future directions.

## Introduction

Mechanistic models of infectious disease (ID) transmission are becoming increasingly used to inform disease control strategies for novel outbreaks of communicable diseases. During the recent COVID-19 pandemic in the UK, ID models were used routinely (through the Scientific Pandemic Influenza Group on Modelling, Operational Subgroup (SPI-M-O) of the Scientific Advisory Group for Emergencies (SAGE)) to provide predictions for key quantities, such as the number of infections and deaths, as well as the potential for hospital and ventilator capacities to be exceeded. Furthermore, these models were routinely used to assess the efficacy of potential policy interventions, including lockdown and vaccination rollout [1–10], which they can do by leveraging the ability of such models for forecasting the future evolution of an outbreak under different scenarios.

However, this also highlighted key areas where current calibration methodologies fell short. Gold-standard methods for model calibration, that account for the large amount of non-ignorable missing data and the mechanistic nature of disease spread, are highly computationally intensive to evaluate. The requirement for models to calibrated in real-time (within 1–7 days at most), meant that models had to be quick to develop, evaluate and fit. This necessarily leads to compromises: usually models were either heavily simplified, losing realism in favour of computational tractability, or they were more realistic but fitted to aggregated data (e.g., national or regional levels—Fig AA in S1 Appendix), losing the ability to make predictions and forecasts at more nuanced resolutions (such as at Lower Tier Local Authority or electoral ward levels—Figs AB–AC in S1 Appendix respectively). Furthermore, there is a need for frameworks to be developed that can be adapted quickly in response to a rapidly changing disease landscape, for example the introduction of new strains of the pathogen or the availability of new data sources. This greatly favours methods that have the potential to be integrated into general-purpose software. To this end, *simulation-based* inference methods are attractive because general-purpose simulation software already exists, e.g., [11–13], but also because infectious disease modellers are adept at developing and evaluating complex simulation models. In any case, simulation models are required for both forecasting and scenario planning, even if they are not used for calibration.

A challenge is that simulation-based inference methods, such as Approximate Bayesian Computation (ABC; [14,15]), often require hundreds of thousands of model runs, even for simple models. For computationally intensive models this quickly becomes infeasible to evaluate. These computational challenges are exacerbated further as the size of the input space grows (i.e., as more parameters are introduced, since there is now a much higher volume of input space to search for good model runs), and also as the size of the output space grows, since it becomes more difficult to find good matches to high dimensional data without introducing substantive approximations [15,16].

In Part 1 [17] we highlighted the utility of Uncertainty Quantification (UQ) methods for the calibration of computationally intensive simulation models. Of key interest are methods that utilise emulators, which are surrogate models that aim to mimic the behaviour of the complex simulation model but are substantially faster to evaluate. This means that emulators can be used in place of the complex simulation model in computationally expensive inference algorithms. These ideas have been successfully applied across a range of fields, including infectious disease models [15,18–25]. In particular, UQ-practitioners argue that incorporating model discrepancy as a key source of uncertainty is essential, to reflect the fact that any model is only an approximation of reality [20,24,26].

Nonetheless, despite the utility of such approaches there remain significant challenges in applying such methods in real-time, and in extending these methods to calibrate to data collected at higher resolutions, for example, if we want to simultaneously track and predict epidemic trajectories in different age-categories across fine spatio-temporal resolutions. This latter idea is the principal motivation for the current paper, driven by the fact that during the COVID-19 outbreak there were key questions—for example assessing the potential impacts of targeted interventions, such as tiered lockdowns or spatio-temporally varying movement restrictions—that required models that could produce accurate forecasts and predictions at the necessary resolution of the intervention. Our goal is therefore to develop methods that facilitate policy-makers being better able to ask the kinds of questions that they want to ask, rather than limit their questions according to the capabilities of current calibration methodologies.

Although there were highly complex and sophisticated simulation models developed during COVID that were specified at high spatio-temporal resolutions [27], to our knowledge it was nevertheless still necessary to fit such models to data at higher-level aggregations, such as the regional and national levels [20], and as such are not optimised for fine-scale forecasting. Therefore we ideally need to develop methods that are able to:

1. incorporate as much epidemiological realism as is required to answer key questions-of-interest;
2. be developed, implemented and updated quickly in response to an emerging outbreak of a novel pathogen;
3. have a flexible way to deal with uncertainties from processes that cannot be directly captured through the model or data, such as the location and frequency of introductions of infection from outside of the system;
4. be able to track epidemic trajectories at fine (e.g., spatio-temporal) resolutions in order to make more nuanced forecasts and allow for the potential for more targeted interventions to be evaluated;
5. be able to deal with data available at different resolutions with differing levels of associated uncertainties;
6. be able to be run in real-time to provide timely information to policy-makers.

In Part 1 [17] we developed a novel simulation-based approach that introduced four main ideas to help tackle some of these problems, which are:

1. Set up a structured model discrepancy (MD) process and embed this within the simulation pipeline. In the models dicussed here we specify an idealised non-uniform zero mean noise process, that explicitly operates to adjust the *hidden states* of the model over-and-above the *simulator* (i.e., the MD is not treated simply as additional observation error). To do this effectively the MD must also be structured such that the adjusted hidden states are epidemiologically valid (that is, states are consistent with each other and the observed data). To this end, we have developed a model discrepancy process that ensures epidemiological validity, but could also be incorporated into general-purpose simulation software (at least for standard compartmental models).
2. Introduce a robust mechanism for building in information from available data to constrain the space of plausible epidemic trajectories. In the UQ field this is often termed ‘data assimilation’. Here we propose using particle filtering to do this [28–30], and simple filters could be readily integrated into existing simulation software (at least for standard compartmental models). Particle filters also generate unbiased estimates of the likelihood, which are typically more precise than naïve Monte Carlo estimators [29,30]. This is fundamentally important for stochastic models, especially when simulating across complex populations such as those involving spatial, network or meta-population structures. Since there are a huge number of potential trajectories that could be realised across the structured population, without some form of data assimilation it is highly unlikely that we will see good model runs that are consistent with the high dimensional data without requiring very large numbers of simulations from the model, even in a perfect model/parameter scenario [15]. This also means that numerical estimates of the likelihood will usually have a much higher Monte Carlo error if particle filtering is not used [29,30]. As such the data assimilation facilitates *much* more efficient inference, as well as improving both prediction and forecasting.

The combination of the embedded MD and data assimilation also tackles several other key challenges that we encountered when trying to calibrate the model to a large number of spatial regions, most notably that of seeding (i.e., the introduction of infections in the early stages of an outbreak), since the MD provides a means to capture the effects of mechanistic processes that cannot be easily implemented in the simulation model. Since, in its idealised form the MD process places higher weight around zero, then the likelihood estimates are penalised if excessive MD adjustments are required to compensate for poor fits of the simulator for a given set of parameters.

3. Exploit the use of fast surrogate models (*emulators*) to efficiently explore the parameter space given that the simulator and associated particle filter are computationally intensive to evaluate. Instead of emulating the model outputs directly, we instead emulate the log-likelihood estimates obtained from the particle filters. By emulating only a single output, this circumvents a key challenge with standard emulation techniques that require separate emulators for each output we wish to calibrate to, which for high-dimensional output spaces could undermine any potential efficiency gains of emulation.
4. We combine this with a calibration technique akin to *history matching*, where we remove poor parts of the input space (where the likelihood is estimated to be close to zero after accounting for major sources of uncertainty). To do this we introduce a novel definition for the Not Ruled Out Yet (NROY) space that accounts for the emulator uncertainty.

**Fig 1:**
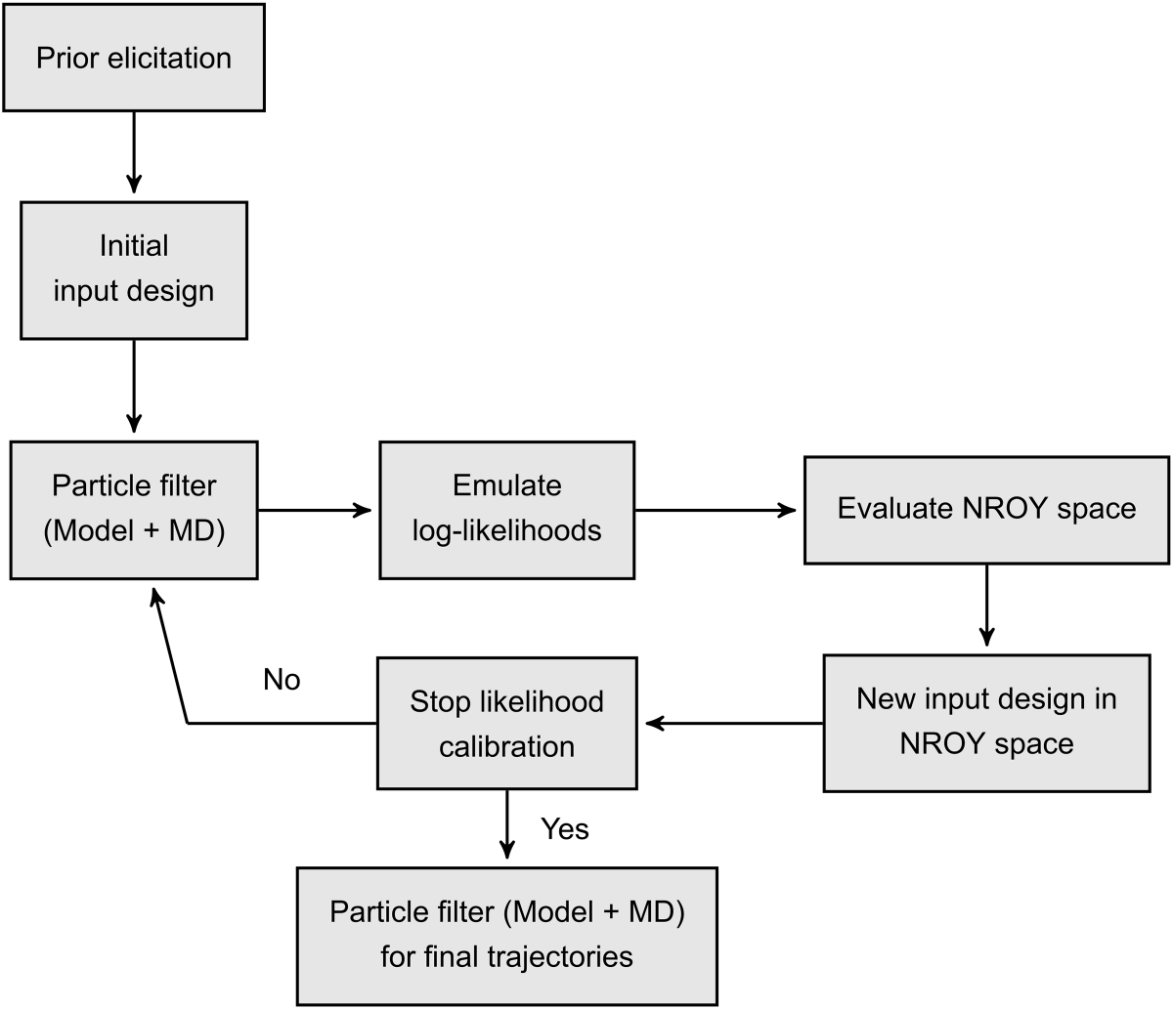
Schematic of the likelihood calibration process. We start with the prior elicitation and initial input design, followed by iterations of (a) particle filtering, utilising both the simulation model and the embedded model discrepancy (MD), and returning log-likelihood estimates; (b) emulation of the log-likelihood surface; (c) evaluation of the Not Ruled Out Yet (NROY) space using the emulator; (d) generation of a new input design within the NROY space.

This generic framework for building models with embedded model discrepancy processes is laid out in Part 1 [17], and a schematic for the calibration process is shown in Fig 1. In this paper we illustrate how these ideas can be extended to work with complex, high-dimensional systems. We also illustrate how this framework can be exploited to help efficiently tackle some further challenges, such as helping to alleviate particle impoverishment. The latter is a particular issue with particle filters where particle weights can concentrate on a small number of particles, which upon resampling results in a lower diversity of particle trajectories [30,31]. A final key challenge with empirical filtering methods is that we can only evaluate a *finite* number of particles, and this affects both the estimates of the filtering distributions and the overall estimate of the log-likelihood (the unbiasedness property of the likelihood being an asymptotic result for infinite particles). It is not uncommon to see applied particle filtering papers use hundreds, or even thousands of particles. However, complex models can severely limit the numbers of particles that can be evaluated, due to both the length of time taken to evaluate the filters and also the memory requirements to store each particle. Only being able to run a small number of particles affects the efficiency of the particle filters, in terms of both their ability to track the observed trajectories, but also by decreasing the precision of the resulting log-likelihood estimates.

The motivating example for this work is a spatially-explicit transmission model of COVID-19 infection, defined across a meta-population of Lower Tier Local Authority (LTLA) districts in England and Wales (around 338 regions, see, e.g., Fig AB in S1 Appendix or Figs 3–5). This model was developed during the 2020–2021 pandemic because it could have provided useful insights into localised spread, and the impact of movement restrictions. However, it was not possible to calibrate this model effectively in real-time, which hindered its practical use and motivated this series of papers. For this model, the observed data on hospitalisations and deaths are given at different spatial resolutions, requiring extensions to both the model structure and filtering algorithms that we developed in Part 1 [17]. In the *Materials and Methods*, we describe the data, model structure, embedded model discrepancy, and observation processes; before describing the bootstrap particle filter. In the *Results* we discuss the results of a simulation study, as well as models fitted to the real data. We discuss the impacts of these and other challenges, as well as avenues for future work in the *Discussion*.

## Materials and methods

### Data

All data processing and visualisation was done in the R statistical language [32] principally using the tidyverse [33], patchwork [34], sf [35] and areal [36] packages. For a full list of packages used, please see the S1 Appendix. We fitted the model to publicly available data on deaths and hospitalisations, provided under the Open Government Licence v3.0. The data used in this paper are available at https://doi.org/10.5281/zenodo.19847027. Unfortunately many of the original source links for the data are no longer active, however we provide documentation detailing where the data were downloaded from through the Zenodo link above.

The *incidence* of deaths within 28 days of a positive COVID-19 test (i.e., the number of new deaths at each time point) are available either by a subset of the 338 original LTLAs (315 areas), or by age-class (<5, 5–17, 18–29, 30–39, 40–49, 50–59, 60–69, 70 + years) *and* region (‘East Midlands’, ‘East of England’, ‘London’, ‘North East’, ‘North West’, ‘South East’, ‘South West’, ‘West Midlands’ and ‘Yorkshire and the Humber’). Unfortunately, to our knowledge, there are no publicly available data stratified by age-class and LTLA. LTLAs are nested within regions. Spatial maps and lookup tables were found on https://geoportal.statistics.gov.uk from the Office for National Statistics (licensed under the Open Government Licence v.3.0), and maps contain OS data (Crown copyright and database right 2022). The data only provide total observed deaths and cannot delineate deaths in hospital from deaths in the community.

Hospitalisation data are provided at yet different aggregations. Hospital *incidence* data (i.e., the number of new patients with COVID-19 admitted to hospital) are available by age-class (0–5, 6–17, 18–64, 65 + years) and NHS-region (‘East of England’, ‘London’, ‘Midlands’, ‘North East and Yorkshire’, ‘North West’, ‘South East’ and ‘South West’). Hospital *case* data (i.e., the number of people in hospital with COVID-19 at a given time) are only available by NHS-region. For the purposes of calibration, counts at the LTLA level can be aggregated to region using the lookup tables described above, and can then be mapped to the corresponding NHS-region, assuming that both ‘East Midlands’ and ‘West Midlands’ map to the ‘Midlands’ NHS region, and that both ‘North East’ and ‘Yorkshire and the Humber’ map to ‘North East and Yorkshire’. Although not perfect, for the age to NHS-age categories we map: < 5 → 0–5; 5–17 → 6–17; (18–29, 30–39, 40–49, 50–59) → 18–64; and (60–69, 70+) → 65 + years.

The movement data at the electoral ward level are provided in the MetaWards package [13], but were mapped from the original 2011 wards to 2019 wards using areal interpolation [36]. We then aggregate movements from the electoral ward level (>8,000 areas) to the LTLA level (338 areas), which is feasible since wards are geographically nested within LTLAs. Specific movements of groups of people between each pair of spatial locations is known as a *movement cohort*. Note that the movement (commuter) data includes wards in Wales, but the death and hospitalisation data only includes LTLAs and regions in England, and so although the underlying model simulates transmission within and between LTLAs in England and Wales (338 regions), we only calibrate using simulated counts and data from England (315 regions). Since the commuter data does not delineate by age, we split the population in each LTLA into age-categories according to the proportions of people in each age-class in the overall population, and then did the same for each movement cohort. These age-breakdowns were derived from publicly available records from the 2011 census licensed under the Open Government Licence v3.0 [37]. For comparison, Fig A in S1 Appendix shows the comparative number and size of wards, LTLAs and regions in England and Wales in 2019 (for consistency we only show the areas that map to the LTLAs that we have death data available for).

### Model structure

**Fig 2:**
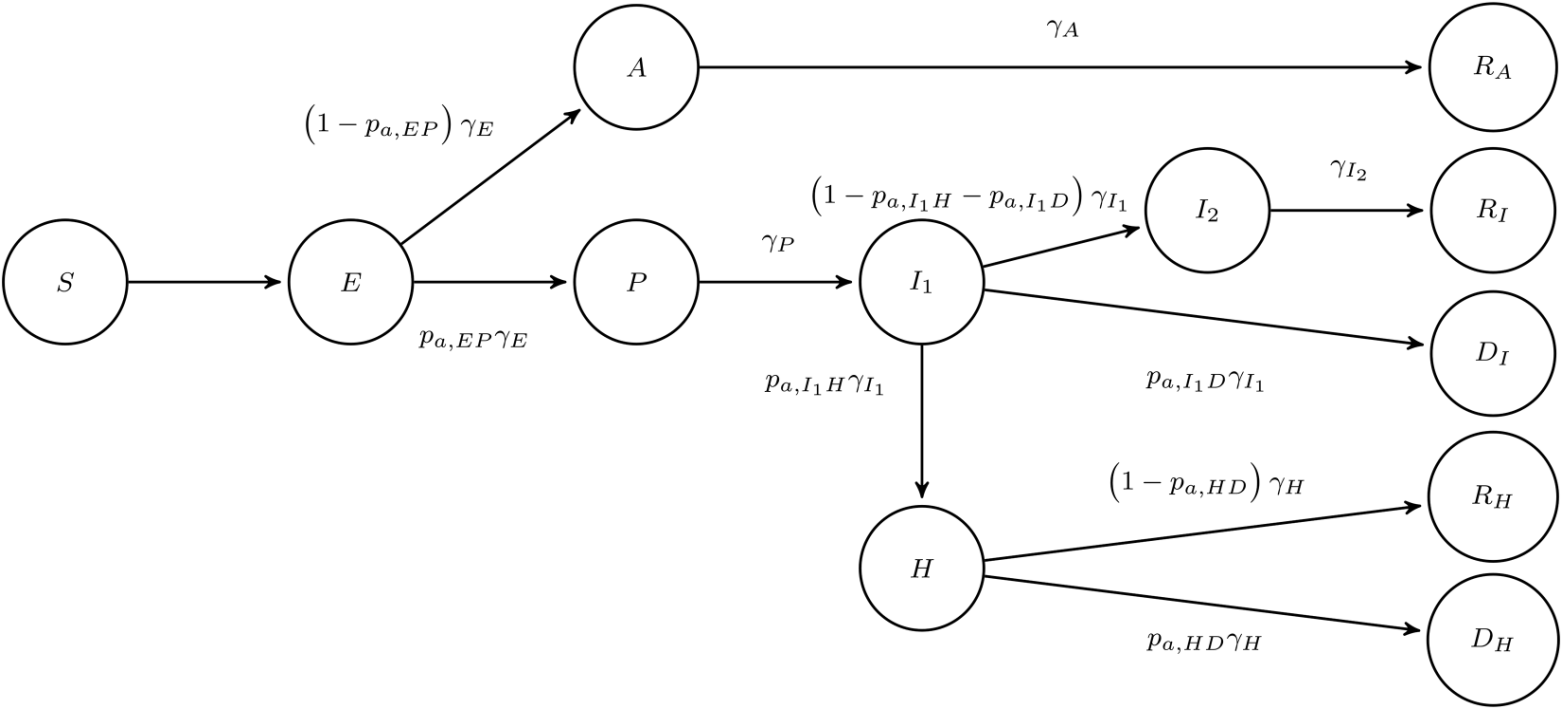
Schematic of the COVID-19 model considered in this paper and an example of the compartmental structure we consider throughout.

For ease-of-reference the specific model structure for our COVID-19 model, introduced in Part 1 [17], is reproduced in Fig 2. Briefly, all individuals begin in the *S* (susceptible) state. Transmission occurs through contact with individuals in any of the infectious states (*A*, *P*, *I*_1_ or *I*_2_), and on infection they enter the exposed (*E*) state, where they are infected but not yet infectious. Individuals will then progress through either the *asymptomatic* pathway, where they will eventually recover ($A\to R_{A}$), or they progress through the *symptomatic* pathway, passing first through a pre-symptomatic state *P*, and then onto a symptomatic state *I*_1_. Some symptomatic individuals will remain in the general population and eventually recover ($I_{2}\to R_{I}$), some will die without going to hospital (*D*_*I*_), and some will be hospitalised (*H*). Some hospitalised individuals will eventually die (*D*_*H*_), whilst some will eventually recover (*R*_*H*_). We assume that all recovered individuals have immunity to reinfection at the current time (since we are only modelling the short-term dynamics here). The model is specified in discrete-time over daily time intervals, and the probabilities of transitioning along different pathways are dependent on age, such that older individuals are more likely to transition down more severe pathways. Whenever we see forks in the pathways in Fig 2 we have multinomial transitions, otherwise we have binomial transitions. For brevity, full details of the transition probabilities and parameterisation of the model can be found in Section C in S1 Appendix.

#### Notation.

We use *X* to denote counts arising from the *transmission model*, *Y* to denote counts adjusted for the model discrepancy process, and *Z* to denote *observed* counts. We use primes to denote *incidence* (e.g., $X^{\prime }$, $Y^{\prime }$, $Z^{\prime }$), and subscripts to denote specific states, time points, age-classes and spatial areas. We assume that we have *N*_*a*_ age-classes, *N*_*s*_ spatial areas, *N*_*r*_ regions, *N*_*b*_ NHS age-classes, *N*_*n*_ NHS-regions and *N*_*t*_ time points.

At time *t*, we let $X_{t}=\left\{X_{tas};a=1,\ldots ,N_{a}\;\text{and}\;s=1,\ldots ,N_{s}\right\}$, where


$$\begin{array}{c} X_{tas}=(X_{tas,S},X_{tas,E},X_{tas,A},X_{tas,R_{A}},X_{tas,P},X_{tas,I_{1}},\\ \;X_{tas,I_{2}},X_{tas,R_{I}},X_{tas,D_{I}},X_{tas,H},X_{tas,D_{H}},X_{tas,R_{H}}), \end{array}$$

is a vector of counts from the *transmission model* at time *t* for age-class *a* in spatial area *s*. Similarly, we let $Y_{t}=\left\{Y_{tas};a=1,\ldots ,N_{a}\;\text{and}\;s=1,\ldots ,N_{s}\right\}$, where


$$\begin{array}{c} Y_{tas}=(Y_{tas,S},Y_{tas,E},Y_{tas,A},Y_{tas,R_{A}},Y_{tas,P},Y_{tas,I_{1}},\\ \;Y_{tas,I_{2}},Y_{tas,R_{I}},Y_{tas,D_{I}},Y_{tas,H},Y_{tas,D_{H}},Y_{tas,R_{H}}), \end{array}$$

is a vector of counts *adjusted for model discrepancy* (MD) at time *t* for age-class *a* in spatial area *s* (see the *Model discrepancy* section for more details).

As described above, the observed data are not available at the same spatial resolution as the simulator and model discrepancy processes, and as such we let $Z_{t}=\{Z_{tar,D}^{\prime },Z_{ts,D}^{\prime },Z_{tbn,H}^{\prime },Z_{tn,H};a=1,\ldots ,N_{a};r=1,\ldots ,N_{r};$ $s=1,\ldots ,N_{s};b=1,\ldots ,N_{b}\;\text{and}\;n=1,\ldots ,N_{n}\}$ denote the available data that we calibrate to. Here the *D* state corresponds to the sum of *D*_*I*_ and *D*_*H*_. The later *Observation distributions* section describes how we map the MD-adjusted states ***Y***_*t*_ to the observed states ***Z***_*t*_.

#### Movement cohorts.

We use the framework set out in [38] to model the impact of movements of individuals between spatial regions on the outbreak dynamics. The movement data has two components: *work* and *play* movements. Work movements consist of fixed moves of individuals between different LTLAs each day for the purposes of work. Conversely, play movements are governed by a stochastic process, where the numbers of individuals moving between specific LTLAs on a given day are governed by a series of multinomial distributions. Following the structure used in the MetaWards package [13,38], we assume that movements happen only during the day, and then all individuals return to their home LTLAs at night.

The transmission process is then applied twice at each time point, once during the day and once at night. As such the force-of-infection and the number of susceptibles in a given LTLA differs between the day and night due to movements. Furthermore we scale the FOI in each LTLA by 0.7 during the day, and 0.3 at night to reflect the relative lengths of the day and night. To ensure that individuals are moved correctly we keep track of the number of individuals in each state in each *movement cohort*, defined as a group of individuals who move between a specific pair of LTLAs. The number of movement cohorts is therefore much larger than the number of LTLAs, but from these we can reconstruct the numbers of susceptibles and infectives in each LTLA during both the day and night so that we apply the correct transmission process. Given a number of new infections in an LTLA, we can then redistribute these across the movement cohorts proportionately to the number of susceptibles in each cohort that maps to each given home LTLA (see Section F in S1 Appendix).

All other transitions are assumed to happen at the end of the day, and we can simulate these within each movement cohort directly. This is because the probabilities of transition for individuals are fixed. As such the process of using binomial/multinomial sampling in each cohort and then summing these up within each LTLA is equivalent to sampling the transitions at the LTLA-level directly and then redistributing them proportionately to the relative counts.

#### Lockdown.

To capture the impacts of lockdown we introduce additional parameters: firstly, work and play movements happen at random with a probability $p_{\text{move}}$, which represents the effect of lockdown on the probability of movements occurring; and secondly, the force-of-infection in each LTLA [eqn. (A.1) in S1 Appendix] is multiplied by a parameter $0<\beta_{s}<1$, to model the impact of interventions such as social distancing. We also swap the contact matrices in the force-of-infection terms [eqn. (A.1) in S1 Appendix] from one derived from the POLYMOD survey [39] in the early stages of the outbreak, to the one derived from the CoMix survey after the first lockdown [40]. As before, we start the modelling on 15^th^ February and fit to data up to 9^th^ April 2020, corresponding to 54 days in total, with forecasts generated for a further two-weeks.

#### Model discrepancy.

Following Part 1 [17], for *absorbing states* (*D*_*H*_, *R*_*H*_, *D*_*I*_, *R*_*I*_ and *R*_*A*_) we place the model discrepancy (MD) on the *incidence* (i.e., new cases) rather than the state counts, and for all other states we place the MD on the numbers of individuals in each state directly. The MD terms are assumed conditionally independent within each age-class and area. In Part 1 [17] we outlined a general approach that ensures the MD process produces states that are *epidemiologically valid*. For example, we can’t have more deaths than infections, or more hospitalisations than symptomatic cases and so on. This approach also ensures that the counts are truncated appropriately (e.g., we can’t have negative counts). As a result, although the MD is formulated as an *idealised* zero-mean noise process—with more weight placed around values of zero—in practice it is necessary to derive a series of ordered conditional discrete truncated distributions. To ensure validity we progress backwards through the epidemiological pathways by adding MD to the later states to begin with. As such the discrepancy added to the earlier epidemiological states is dependent on the discrepancy added to the later epidemiological states. In this way the methodology differs significantly from just adding independent Gaussian noise to the hidden states.

In Part 1 [17] we made a case that a natural choice for an MD distribution for the models/data under consideration here is the Skellam distribution, and we set up a framework for how to utilise and parameterise this distribution. The Skellam distribution can be approximated by a truncated discrete Gaussian distribution, which can be more computationally efficient, and in this paper we decided to use truncated discrete Gaussian distribution throughout. Although we originally posited this as an approximation to the Skellam, it is perfectly reasonable to use this as a direct alternative to the Skellam that has many of the same properties. Another motivation for this change was to explore ways in which the structure of the truncated discrete Gaussian might be leveraged to provide more efficient look-ahead particle filters, although we do not present any of that exploratory work here, but return to these ideas in the *Discussion*.

We denote a random variable W∈ℤ from a truncated discrete Gaussian distribution, with mean μ, variance $\sigma^{2}$, lower bound LB∈ℤ and upper bound UB∈ℤ as:


$$\begin{array}{c} W\sim tN^{D}\left(\mu ,\sigma^{2},LB,UB\right), \end{array}$$

for −∞<μ<∞, σ>0 and *LB* ≤ *W* ≤ *UB*. The probability mass function, and how to sample from this distribution efficiently, is described in S1 Appendix for Part 1.

The MD for arbitrary state *c*, $\Delta_{tas,c}$, is specified as:


$$\begin{array}{c} \Delta_{tas,c}\sim tN^{D}\left(0,2a_{\text{MD}}+2b_{\text{MD}}W_{tas,c},{\text{LB}}_{tas,c},{\text{UB}}_{tas,c}\right) \end{array}$$ (1)

where *W*_*tas*,*c*_ corresponds to either the simulator incidence or count for the given state *c* (depending on which state the MD is being applied to) and parameters $a_{\text{MD}}$ and $b_{\text{MD}}$ allow the MD variance to increase as counts increase (see Part 1 [17] for more details and discussion). The full specification and derivation of the model discrepancy terms and bounds are given in Section E in S1 Appendix. We also found it was necessary to add in some additional parameters that enabled the MD process to be phased in at different times in different spatial regions to reflect the fact that introductions of infections were not uniform across the country in the early stages of the outbreak (see Section C.2 in S1 Appendix for full details, and we return to this in the *Discussion*).

#### Observation distributions.

As discussed in the earlier *Data* section, a key methodological challenge that arose during model development is that the movement data (and thus the simulation process) is specified at the LTLA-level (by age-class), but the data that we wish to calibrate to are not available at the same resolutions. Furthermore, the catchment areas of hospitals do not map cleanly to administrative boundaries, such as LTLAs, which makes integrating hospital data with death data even more difficult. Various methods were proposed during the pandemic to try to map NHS data to different spatial scales [41,42], but this remains a considerable challenge. Although we discuss these issues in the context of our COVID-19 model in England and Wales, we believe that these kinds of model-to-data mapping issues are likely to arise in other systems, especially when simulation models are being developed at finer resolutions and when data are being pulled in from different sources, especially as data availability changes as we might expect in an emerging outbreak. In this manuscript, given that we only have age-level data for deaths at the *regional* level, we decided to use a hierarchical observation process to map the LTLA/age-level information from the model to the higher-level aggregations in the data in a sensible way, whilst allowing the observation process model to reflect additional uncertainties in the mappings and a lack-of-independence between data sources. Full details of this approach are given in Section G in S1 Appendix.

#### Bootstrap particle filter.

In this manuscript we applied a bootstrap particle filter (BPF) [28] in order to better track the spatio-temporal trajectories in the different age-classes, and provide estimates of the log-likelihood for a given set of parameters. The BPF is also straightforward to implement despite the fact that the data are recorded at different scales to the hidden states of the model. This is because the observation process only determines the particle weights, and does not play a role in the forward simulations of the hidden states outside of the particle re-sampling step [28,30]. Look-ahead filters, such as the auxiliary particle filter [43] or twisted particle filter [44], are likely to produce better estimates of the filtering distributions and log-likelihoods, but the standard versions of such filters require the data to be specified at the same scale as the hidden states. We return to this as a key area for future research in the *Discussion*.

The BPF for our model is shown in Algorithm B in S1 Appendix. We note that this has the potential to be built into general-purpose software, because software to simulate from general compartmental models in meta-population settings already exists [12,13] and we have developed a generic way to specify the model discrepancy process for a compartmental model in Part 1 [17]—see also the *Model discrepancy* section. The more difficult component to generalise is the observation process, which is likely to be situation dependent. Nonetheless, the kind of approach developed in the *Observation distributions*—detailed fully in Section G in S1 Appendix—lends itself to being more generalisable.

In Section I.1 in S1 Appendix we also incorporate some novel amendments to the standard BPF to help alleviate particle impoverishment using Markov chain Monte Carlo (MCMC; [45]) steps [30,31], specifically by leveraging the structure of the model to do this in a more computationally efficient way that reduces the number of evaluations of the complex simulator that needs to be done, relative to a naïve implementation.

#### Model implementation.

The meta-population framework described above and developed originally in [38] is implemented in the open-source MetaWards software [13] (available at https://metawards.org/). Indeed originally we coded the simulator up in MetaWards, although currently this software does not natively support particle filtering, and as such we rewrote our specific model in R using the Rcpp [46] and RcppArmadillo [47] packages, which allowed us to implement the MD process and bootstrap particle filter developed here. For the emulators we used deep Gaussian Processes (DGPs), using the approach of [48], implemented in the dgpsi package in R [49]. Likelihood calibration was performed using the hmer package in R [50]. For a full list of packages used, please see Section A in S1 Appendix. All code used to run the models in this paper can be found at https://doi.org/10.5281/zenodo.19847027. Derivations of the initial plausible regions for the parameters of our initial COVID-19 model are given in Part 1 [17] and discussed in Section K in S1 Appendix.

#### Emulation and likelihood calibration.

For Wave 1 we generated an initial input design using Latin Hypercube Sampling for the uniform ranges, and then augmented this with a space-filling design over the non-uniform ranges as described in Part 1 [17]. For each wave we generated a set of 200 design points, and a further set of 50 independent validation points.

For each design point $\theta_{i}$ we ran the BPF in Algorithm B (in S1 Appendix) and generated an estimate of the log-likelihood, $l\left(\theta_{i}\right)$. Using dgpsi, we then trained a two-layer DGP emulator with a heteroscedastic Gaussian likelihood function on these runs, and validated the emulator using the hold-out validation runs. To reduce the complexity of the emulator, we selected a set of active variables using automatic relevance determination (ARD) where we fitted an initial standard GP, and then removed any input parameters with large length-scales. We chose length-scale thresholds for each wave by comparing the estimated length-scales with each other, and then checking that the validation plots were not significantly affected by the removal of the inactive variables. Then the DGP was fitted to just the active variables identified by the ARD process. If the fitted DGP passed the out-of-sample validation checks, then we augmented the training data with the validation data to produce a final updated emulator. We got much improved validation by emulating h(θ)=log[−l(θ)] instead of l(θ) directly.

As described in Part 1 [17], we developed a novel definition of the Not Ruled Out Yet (NROY) space, such that:


$$\begin{array}{c} \theta_{NROY}=\left\{\theta :P\left(l(\theta )-\hat{l}(\theta^{**})>\log(\epsilon )\right)>1-\alpha \right\}, \end{array}$$ (2)

where $\hat{l}(\theta^{**})$ is the current *maximum* log-likelihood estimate from the simulator runs. Since here we emulate h(θ), we note that


$$\begin{array}{cc} P\left(l(\theta )-\hat{l}(\theta^{**})>\log(\epsilon )\right) & =P\left(-e^{h\left(\theta \right)}-\hat{l}(\theta^{**})>\log(\epsilon )\right)\\ & =P\left(h\left(\theta \right)<\log\left[-\hat{l}(\theta^{**})-\log(\epsilon )\right]\right), \end{array}$$

and thus we can amend (2) accordingly, such that


$$\begin{array}{c} \theta_{NROY}=\left\{\theta :P\left(h\left(\theta \right)<\log\left[-\hat{l}(\theta^{**})-\log(\epsilon )\right]\right)>1-\alpha \right\}. \end{array}$$ (3)

We set α=0.95 with ϵ=0.00001 throughout.

By definition, $\theta^{**}$ is in the tails of the emulated log-likelihood surface, and so on rare occasions the emulator will pass all usual diagnostics but rule-out $\theta^{**}$ if the emulator does not capture the tails of the log-likelihood surface well enough. To ensure that $\theta^{**}$ is always retained in the NROY space, we adapted the local Voronoi tessellation approach of [51], where we retain regions around a set of ‘doubt points’, which are points that the emulator wants to rule out, but for which there is some evidence should be retained. To generate doubt points we first select all the training and validation points that the emulator wishes to rule out (using the NROY definition based on the emulator in (3)). Then, for each *ruled-out* point we calculate a version of this measure based on the log-likelihood estimates from the particle filters directly, rather than through the emulator approximations (in some papers this would be akin to the so-called ‘simulator implausibility’—see, e.g., [50]). Doubt points ($\theta^{D}$) are then the subset of ruled-out points that would *not* be ruled out by the following measure:


$$\begin{array}{c} \theta^{D}=\left\{\theta :P\left(h\left(\theta \right)<\log\left[-\hat{l}(\theta^{**})-\log(\epsilon )\right]\right)<1-\alpha \;\text{and}\;-e^{h\left(\theta \right)}-\hat{l}(\theta^{**})>\log(\epsilon )\right\}. \end{array}$$ (4)

If $\theta^{D}$ is empty, then we can proceed with our calibration procedure as normal. If $\theta^{D}$ is not empty, then we further augment $\theta^{D}$ by including any design point which is closer to a doubt point than any other design point, where distance is measured using the emulator posterior covariance function. This final set of augmented doubt points are automatically retained in the NROY space, but furthermore any new point that lies closer to a doubt point than any other design point, is automatically retained in the NROY space, where distance is once again defined using the emulator posterior covariance function.

Once we have defined the remaining NROY space in each wave, we used the slice-sampling approach of [22]—implemented in hmer—to sample from it. To generate a space-filling design for subsequent waves, we generated a large number of samples from the NROY space before sub-sampling these using a *maximin* design. For a consistent comparison, we ran 10 waves of likelihood calibration for each example.

### Simulation and real-data studies

As a test of the methodology we simulated an outbreak where the parameters we used for the simulation are shown in Table B in S1 Appendix. To initiate the simulation infections were introduced randomly through the model discrepancy process in different spatial regions. We introduced a lockdown at day 37, and ran the simulation for 68 days in total. We applied the observation process described in the *Observation distributions* section to generate ‘observed’ data at the same levels of aggregation as seen in the real data. We fitted the model to data up to day 54, but then simulated the particle trajectories forwards to generate probabilistic forecasts for the final two-weeks so that we could assess how well we could calibrate to the observed data and how the forecasts performed. The simulation study also allows us to assess whether we are picking up the true parameter values in the NROY space. As a comparator we also fitted to the same simulated data set but assuming that the observed hospital and death data was available at the same resolution as the model.

We then fitted the model to the real data, initially up to the first lockdown, and then a second run up to day 54 (so beyond the first lockdown). We compared the particle trajectories and forecasts to the observed data to assess the model fits and forecasting performance.

## Results

The runs of the particle filters were conducted on the JASMIN High Performance Computing (HPC) facility [52] (https://jasmin.ac.uk). Emulation and likelihood calibration were conducted on standard desktop/laptop machines. Amongst all of the different scenarios below, the minimum time taken for a single particle filter to run was 1.7 hours, and the longest was 11.6 hours. To balance out HPC resource requests, we decided to run each of the 250 design/validation points in a given wave in parallel on JASMIN, over 250 independent nodes (so each design point was evaluated in serial). We note that given multiple cores on each node, runtimes could be further improved by parallelising various aspects of the particle filter, at the cost of requiring additional CPU capabilities. Nonetheless, evaluating the model, even with a small number of particles, is highly computationally expensive, and as such this further justifies the use of emulation methods.

### Simulation studies

We conducted 10 waves of likelihood calibration, using the approach described in the *Emulation and likelihood calibration* section, with 200 design points and 50 validation points at each wave (corresponding to a standard 80:20 split of training to validation samples). We fitted the model to data up to day 54, but then simulated the particle trajectories forwards to generate probabilistic forecasts for the final two-weeks. The mean time to run the particle filter across all waves was 3.9 hours, with a range of 2.7–6.1 hours.

**Fig 3:**
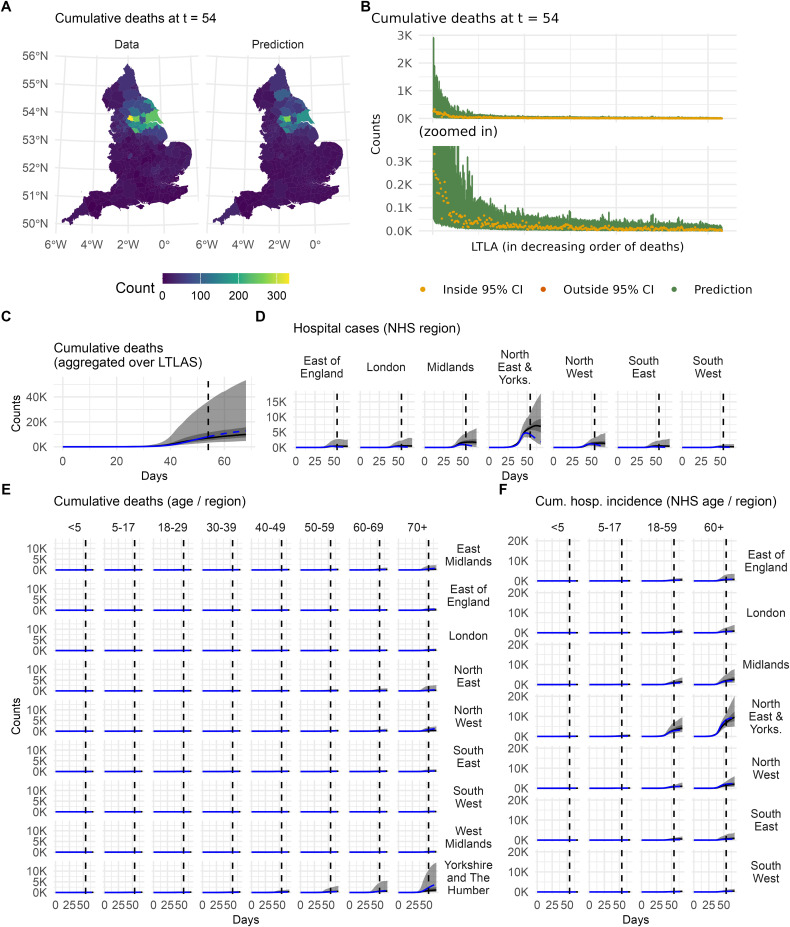
Particle trajectory plots across the ensemble of design points at Wave 10 for the simulated outbreak. **A)** Spatial (LTLA-level) plots of the mean number of deaths by day 54, for the data and the fitted model. **B)** Cumulative deaths at day 54 within each LTLA (ranked in decreasing order of predicted deaths). **C)** Cumulative deaths over time, aggregated over the 315 LTLAs. **D)** Hospital cases over time in each NHS region. **E)** Cumulative deaths over time in each age/region category. **F)** Cumulative hospital incidence over time by each NHS age/region category. In plot B) the green points are the predicted ensemble means, and the error bars are the 95% prediction intervals. For clarity we show a zoomed-in version of the plot also. The yellow and red points are the observed data coloured by whether they lie inside and outside of the prediction intervals respectively. In plots C–F, the blue dashed lines correspond to the observed data and the black solid lines to the mean trajectories from the particles taken across the ensemble. The ribbons correspond to 50% and 95% prediction intervals. A vertical dashed line corresponds to the end point of the observed data, such that trajectories before the line are generated from the particle filter, and trajectories to the right of the line are simulated forecasts from the model. Source for shapefiles: https://geoportal.statistics.gov.uk from the Office for National Statistics licensed under the Open Government Licence v.3.0. Contains OS data: Crown copyright and database right 2022.

Fig 3 summarises the particle trajectories across the ensemble of design points at Wave 10 fitted to the simulated data at day 54. We can see that the epidemic started in Yorkshire and the North East, and that the particle trajectories seem to be tracking the observed data well across the various resolutions. We note that the two-week forecasts also seem good here, with the uncertainty bounds containing the true (but unobserved) trajectories. For comparison, Fig B in S1 Appendix shows the particle trajectories over the initial Wave 1 input space, and here we can see that even with the filtering the particle trajectories have a very large variance over the initial plausible region. This illustrates the improvement in model fit through successive waves of calibration. In Fig 3 and Fig B (in S1 Appendix) we keep the *y*-axes the same across the different sub-plots to highlight the spatio-temporal heterogeneity between different regions and age-classes. For contrast, Figs D and C in S1 Appendix show the same plots but allowing the *y*-axes to vary to more easily show the model predictions relative to the data.

We reiterate that there is *no explicit seeding process* in this model, instead we simply allow the MD process to introduce infections in the early stages and then let the particle filter guide the trajectories using information in the available data. We note also that we can obtain estimates for all the underlying hidden states, which for the Wave 10 particles are shown in Fig O in S1 Appendix. We can see that these match the true (but hidden) trajectories fairly well. It is worth noting that the model is predicting fewer hospital deaths in the oldest age-category than the true trajectory, but since the model is only being fitted to total deaths (i.e., *D*_*I*_ + *D*_*H*_), there is limited information in the data to identify the true trajectories of *D*_*I*_ and *D*_*H*_ separately.

Figs S and T in S1 Appendix show the evolution of the NROY space over the different waves for the parameters of the model, with the true values of the parameters shown as red points. These show that over successive waves the NROY space becomes a small proportion (≈1.7 × 10^−9^) of the original input space, and that at Wave 10 the true values are contained within the NROY space, suggesting that the calibration process is honing in on the correct region of parameter space. We can also see that we cut more space out for some variables than others, and this is likely due to some variables remaining inactive across the different waves. We discuss this in more detail in the *Discussion*. Overall we can see that the approach is working well.

#### Simulation study assuming availability of data at the correct model aggregations.

We also ran 10 waves of likelihood calibration on the simulated data assuming that the observed hospital and death data was available at the same resolution as the model (i.e., at the age / LTLA level). The mean time to run the particle filter across all waves was 4 hours, with a range of 2.9–11.6 hours. For brevity the results are shown in the S1 Appendix, with the ensemble particle trajectories for Waves 1 and 10 shown in Figs K–L and Figs M–N respectively. Again, by Wave 10 the model is doing a good job of fitting to all spatial locations in all age-classes, except perhaps the number of hospital deaths in the oldest age-class, where the model is underpredicting the number of deaths in the LTLAs with the highest observed number of deaths at day 54. In the *Simulation studies* section we noted that the previous model underestimated the ‘true’ number of hospital deaths, most likely due to the model being fitted only to total observed deaths, and not by hospital and community deaths simultaneously (e.g., Fig O in S1 Appendix). If we look at the predicted hidden states for the model fitted to the new data—Fig R in S1 Appendix—we can see that the model is still underpredicting the *D*_*H*_ state, but not by as much as the previous model, suggesting (as expected) that this increase of information in the observed data results in better predictions of the hidden states, even if not perfect. To explore this behaviour in more detail, we can also see that once again at Wave 10 the NROY space is a small proportion (≈1.1 × 10^−8^) of the original input space (Figs Y–Z in S1 Appendix), but in particular the variables $\eta_{I}$ and $\eta_{H}$ (which allow the relative hospital and community death rates by age to vary) are not yet well-identified, even with the increased information in the data. The fits could potentially be improved by running more calibration waves, however in this case only 12% of the NROY space was removed after Wave 9, and examination of the validation plots from the emulator suggested that the variance between log-likelihood estimates across the NROY space is high enough that we are unlikely to reduce space substantially more without reducing the Monte Carlo error of the particle filter estimates. We will return to this challenge in the *Discussion*.

### Real data

For the real data we have the additional complexity that the hospitalisation data is only available from 19^th^ March 2020, whereas the death data is available from 2^nd^ March 2020. Hence the likelihood terms for the observation process need to be adjusted to target only the available data at any given time point.

#### Fitted to data up to the first lockdown.

Since we are unsure of the initial conditions, we choose to run the model from 15^th^ February 2020, up to the first lockdown on the 23^rd^ March 2020, a total of 37 days. We assume that deaths from COVID-19 are zero between 15^th^ February–2^nd^ March, but leave the hospitalisation data missing until 19^th^ March. We assume the population was fully susceptible on the 15^th^ February, and allow the MD process to introduce infections as required. We then produce forecasts for the following two-weeks.

**Fig 4:**
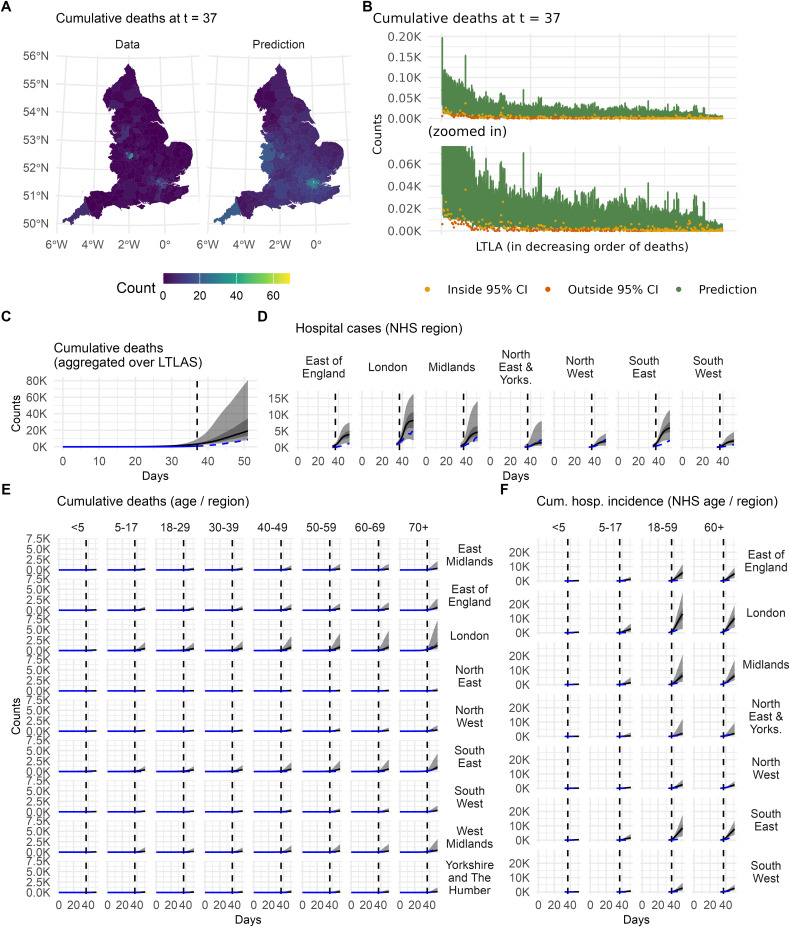
Particle trajectory plots across the ensemble of design points at Wave 10 for the real UK data up to the first lockdown. **A)** Spatial (LTLA-level) plots of the mean number of deaths by day 37, for the data and the fitted model. **B)** Cumulative deaths at day 37 within each LTLA (ranked in decreasing order of predicted deaths). **C)** Cumulative deaths over time, aggregated over the 315 LTLAs with death data available. **D)** Hospital cases over time in each NHS region. **E)** Cumulative deaths over time in each age/region category. **F)** Cumulative hospital incidence over time by each NHS age/region category. In plot B) the green points are the predicted ensemble means, and the error bars are the 95% prediction intervals. For clarity we show a zoomed-in version of the plot also. The yellow and red points are the observed data coloured by whether they lie inside and outside of the prediction intervals respectively. In plots C–F, the blue dashed lines correspond to the observed data and the black solid lines to the mean trajectories from the particles taken across the ensemble. The ribbons correspond to 50% and 95% prediction intervals. A vertical dashed line corresponds to the end point of the observed data, such that trajectories before the line are generated from the particle filter, and trajectories to the right of the line are simulated forecasts from the model. Source for shapefiles: https://geoportal.statistics.gov.uk from the Office for National Statistics licensed under the Open Government Licence v.3.0. Contains OS data: Crown copyright and database right 2022.

As before we conducted 10 waves of likelihood calibration, and the mean time to run the particle filter across all waves was 2.9 hours, with a range of 1.7–5.3 hours. The particle trajectories for Wave 10 are summarised in Fig 4, and a comparison to the Wave 1 trajectories (Fig E in S1 Appendix) shows a substantial improvement in model fit over successive waves of likelihood calibration. (Again, Figs G and F in S1 Appendix show the same plots but allowing the *y*-axes to vary to more easily show the model predictions relative to the data.)

Here we can see that the model is doing a good job of estimating the spatial heterogeneity in the observed numbers of deaths, and is generally fitting most observed trajectories well. It seems to overpredict the cumulative deaths in various LTLAs (Fig 4B), resulting in a smoother distribution over space than seen in the data (Fig 4A). This could be for various reasons, the predominant one being that there is much less information in the dataset at this point than we had in the simulated data, especially since hospitalisations are only available 4 or 5 days prior to the first lockdown. This has the knock-on effect that in the early stages of the outbreak the only data that can be used to inform the particle weights is found right at the end of the epidemiological pathways described in Fig 2. Given a finite number of particles this can lead to a lag-effect, such that by the time there is sufficient information in the data to constrain the trajectories, they are already moving away from the truth. We note that this is a generic challenge with using boostrap particle filters, but is felt more acutely here due to the lack of hospitalisation data in the early stages, and the small number of particles that we are able to run. We discuss these challenges in more detail, and possible ways they could be alleviated in future work in the *Discussion*.

Fig U in S1 Appendix shows the input spaces for the $t_{r,\text{MD}}$ terms, and we can see very clearly the spatial variation, with London requiring MD to be added earlier than the rest of the country, and the North West much later. Fig V in S1 Appendix shows the rest of the parameters, and we can see that by Wave 10 the NROY space is a very small proportion (≈1.4 × 10^−13^) of the original input space, despite the paucity of available data compared to the simulation study.

#### Fitted to data beyond the first lockdown.

As before, we start the modelling on 15^th^ February, with the first lockdown coming into place on 23^rd^ March 2020. We fit to data up to 9^th^ April 2020, corresponding to 54 days in total, with forecasts generated for a further two-weeks.

**Fig 5:**
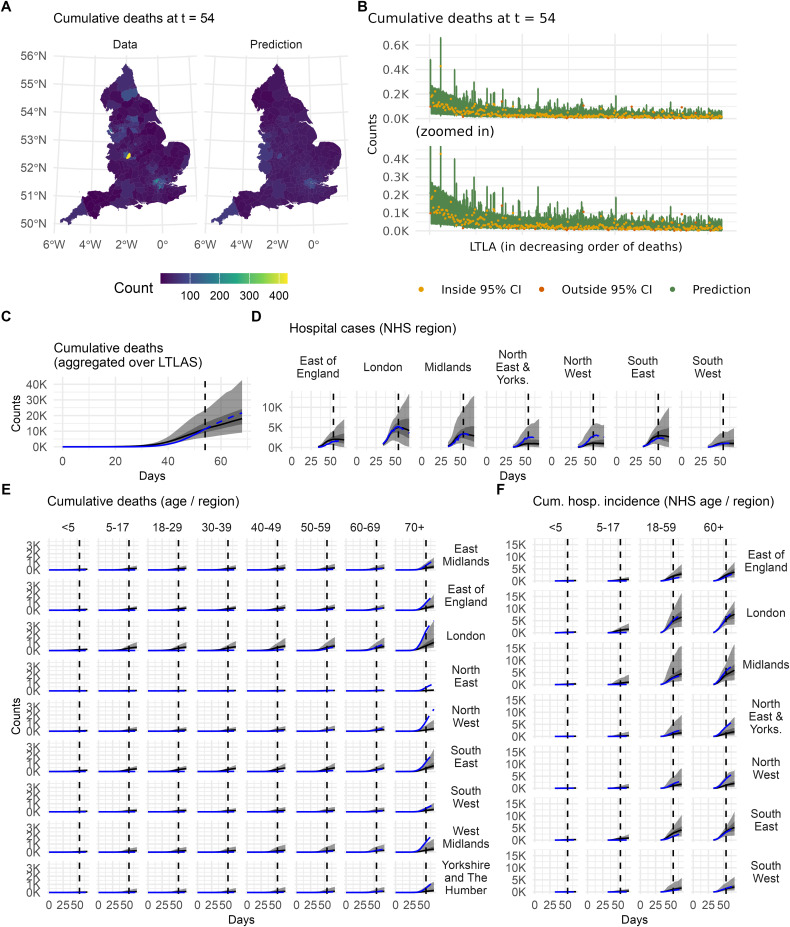
Particle trajectory plots across the ensemble of design points at Wave 10 for the real UK data beyond the first lockdown. **A)** Spatial (LTLA-level) plots of the mean number of deaths by day 54, for the data and the fitted model. **B)** Cumulative deaths at day 54 within each LTLA (ranked in decreasing order of predicted deaths). **C)** Cumulative deaths over time, aggregated over the the 315 LTLAs with death data available. **D)** Hospital cases over time in each NHS region. **E)** Cumulative deaths over time in each age/region category. **F)** Cumulative hospital incidence over time by each NHS age/region category. In plot B) the green points are the predicted ensemble means, and the error bars are the 95% prediction intervals. For clarity we show a zoomed-in version of the plot also. The yellow and red points are the observed data coloured by whether they lie inside and outside of the prediction intervals respectively. In plots C–F, the blue dashed lines correspond to the observed data and the black solid lines to the mean trajectories from the particles taken across the ensemble. The ribbons correspond to 50% and 95% prediction intervals. A vertical dashed line corresponds to the end point of the observed data, such that trajectories before the line are generated from the particle filter, and trajectories to the right of the line are simulated forecasts from the model. Source for shapefiles: https://geoportal.statistics.gov.uk from the Office for National Statistics licensed under the Open Government Licence v.3.0. Contains OS data: Crown copyright and database right 2022.

As before we conducted 10 waves of likelihood calibration, and the mean time to run the particle filter across all waves was 4.7 hours, with a range of 3–8.7 hours. The particle trajectories for Wave 10 can be seen in Fig 5, and a comparison to the Wave 1 trajectories (Fig H in S1 Appendix) again shows a substantial improvement in model fit over successive waves of likelihood calibration. (Again, Figs J and I in S1 Appendix show the same plots but allowing the *y*-axes to vary to more easily show the model predictions relative to the data.) Here we can see that the information afforded by the additional 17 days’ worth of data has substantially improved the model fits. In Fig 5B we can again see the lag-effect that was discussed in the previous section, where all the trajectories seem to overpredict in the early stages but then pull back to align with the observed data later on. This is further evidence that we need better particle filters, since it may be that we are inducing some bias in the parameter inference as a means of correcting for the early lag-effect. Figs W and X in S1 Appendix show the evolution of the NROY space over successive waves, and we can again see that the Wave 10 NROY space is a very small proportion (≈5.3 × 10^−14^) of the original input space.

## Discussion

We have developed a novel method for calibrating computationally intensive stochastic infectious disease models in high-dimensions, that utilises emulation and likelihood calibration to perform robust exploration of the parameter space to find the region of high posterior density. Using this approach we have successfully calibrated an age-structured model (8 age-classes) across a meta-population of 338 connected spatial regions (calibrating to 315 of these regions), where the underlying simulation model also includes patterns of movement driven by commuter data and a transmission process governed by contact matrices derived from social mixing surveys. Traditional approaches to this problem require emulators to be built for each output-of-interest, which would be prohibitive here since we want to fit to time-series data across 315 spatial areas and multiple time points simultaneously. We circumvent this problem by emulating a transformation of the log-likelihood surface instead, which is a one-dimensional output and thus only requires a single emulator (see also [53,54]). Alternatively one could fit to aggregated data [20], but this would lose the ability to accurately track epidemic trajectories in high-dimensions.

However, as with almost all infectious disease systems the likelihood function is analytically intractable, since it’s evaluation relies on inferring a (often large) set of hidden states [55–59]. A scalable data augmented/reversible-jump MCMC method was developed during the pandemic and applied to a highly spatio-temporally resolved model of COVID-19 in the UK to directly inform the control efforts [59]. These methods are very powerful, but require that the model and inference algorithm to be intrinsically linked, and thus each new model requires a bespoke MCMC algorithm, which are challenging to code, optimise and run at-scale, and require significant expertise in MCMC methods to develop. Our approach provides an alternative framework, in which the problem of integrating over large (and unknown) numbers of hidden states is done using particle filtering, which generates estimates of the likelihood that rely only on being able to simulate directly from the underlying complex model [28]. This is appealing in practice because it is often far more straightforward to write simulation code than MCMC code for these kinds of models. We therefore view these approaches as being complementary, and both are active areas of ongoing research. In our proposed methodology we also develop a novel model structure where we embed a model discrepancy process into the simulation pipeline, which, alongside the use of particle filtering tackles various challenges, particularly how to deal with introductions of infections from processes that are not explicitly captured in the simulation model (including imports from outside of the UK, which is vital in the early stages of the outbreak to generate plausible seeding in time and space and for which a suitable mechanistic model does not exist).

Particle filtering (and other simulation-based) methods have been used successfully in the past for calibrating infectious disease models, usually by embedding within MCMC [60–65] or SMC^2^ [66] algorithms. However, these methods can be extremely computationally challenging, often requiring large numbers of particles to be run, even in relatively small-scale systems. In the examples explored here, the minimum runtime for a single particle filter evaluation was 1.7 hours, simulating across a 37 day period, but the longest was 11.6 hours simulating across a 54 day period. In future work we would aim to fit models to even longer time periods, and all of this together make it infeasible to embed these kinds of filters in standard MCMC or SMC algorithms.

As a result, we instead use emulation as a fast way to comprehensively explore the input space, and combine this with iterative likelihood calibration to efficiently converge towards the area of high posterior density through a series of waves (since under the uniform priors used here the posterior density is proportional to the likelihood). An emulator can be trained from a relatively small set of training runs of the model, and then used to systematically explore the parameter space to remove areas of the input space where the probability of the true likelihood being within a specified region around the posterior mode is low (the ruled out region). These training runs are can be easily parallelised across design points, and/or within the particle filters as required. The number of training points can be fixed in advance, meaning that the computational burden can be controlled more effectively on HPC clusters. Once trained, the emulators are quick to evaluate, and so large numbers of predictions can be made quickly and efficiently, meaning that the emulator can comprehensively explore the input space in a way that can’t be achieved if we were to rely on the particle filters alone. Log-likelihood surfaces can be challenging to emulate, so we exploit recent advances for efficiently and robustly fitting Deep Gaussian Processes to do this [48]. Of course this still requires multiple waves of likelihood calibration to be done, which adds to the overall computational time, and this means that ways in which we can reduce the number of waves that need to be evaluated will further enhance this methodology. One possiblity in this direction is the development of better look-ahead filters, since this means that log-likelihood estimates will have less uncertainty and thus we should reduce space much more efficiently from wave-to-wave. We fit to publicly available, but incomplete hospitalisation and death data that are available at different degrees of spatial/age-structured hierarchies. Overall we used only 2,500 evauations of the particle filters for each scenario we explored, with the emulators being built and run on standard machines. Although not perfect, the fitted models show good calibration in space-and-time, although with key areas for improvement in future work, as discussed in detail below.

A key novelty we introduce is to embed a structured model discrepancy process into the system. We set this up as an idealised zero-mean noise process that operates across all epidemiological states and allows a mechanism for amending the hidden states based on processes that are not explicitly modelled, such as the introduction of infection in the early stages of the outbreak. *This latter consideration was a major challenge to overcome.* Running the full model in the prior space produced some runs that were consistent with the data at the national level, but not at finer spatial resolutions (not even at the regional-level let alone the LTLA-level). One way to approach this issue is to introduce some spatio-temporal seeding process, such as to have some spatially-explicit background rate-of-infection parameters that exert a constant infectious pressure over time. There are various problems with this approach: firstly, there is little evidence to inform the functional form of these terms (there certainly isn’t a constant pressure in reality), and secondly, this introduces a large number of additional parameters to estimate which increases the volume of the input space dramatically. We tried a range of other approaches for generating seeding events heterogeneously across spatial regions, including a method based on using the first few weeks worth of data to generate empirical priors for the likely states of the system at a fixed point in time by running simpler MCMC models in each spatial region independently. None of approaches we tried worked well, but the embedded MD process, coupled with the use of particle filtering, provided a flexible approach to dealing with this problem, and we have shown in this paper that the method can produce good model fits even without any explicit seeding mechanism in the model. This differs from other approaches to dealing with unknown mechanisms because it allows for model states to be increased *or* decreased with respect to information in the observed data. It also ensures that the added noise results in epidemiologically consistent hidden states. Furthermore, the use of MD also introduces additional uncertainty that represents the fact that the underlying transmission model structure is itself an approximation of reality. The ability to track trajectories in space and time is of vital importance if we want to use models to produce spatio-temporal predictions/forecasts, and/or to explore control policies such as localised lockdowns.

Other recently developed simulation-based calibration approaches include Physics-inspired neural networks (e.g., [67]), which provide an efficient calibration approach for compartmental models, but currently have only been applied to deterministic models. Interestingly, [67] also stated that they had challenges inferring the initial conditions of their model, which our embedded MD process helped us to overcome.

Other recent approaches are aligned with the ideas of *amortised inference* [68], in which complex emulators (such as neural networks) are trained on large numbers of initial runs from the complex simulator, and then leveraged to perform efficient inference. The challenge here is that in high-dimensional input spaces a very large number of simulator runs would be required in order to get a good emulator that works sufficiently well across the whole input space. One advantage of history matching (and our analogous approach) is that it does not require perfect emulators to rule out space, and as such the progression through multiple waves can be efficient in terms of the numbers of simulator runs required (e.g., [15,69]). We use many less simulator runs than [70] in their examples. We also found that if you want good prediction and forecasting power in high-dimensions, then data assimilation is necessary, and this plus the embedded MD process helps to deal with the initial condition problem. Similar challenges can be found in other approaches that rely on large numbers of simulator runs, such as ABC with random forests [71]. These methods are being actively developed, and so we look forward to future developments in these areas.

Despite the utility of our methodology, there are still key challenges to overcome. The main one relates to the effectiveness of the particle filter to build in information across the whole time-series when simulating particles. For example, in Section C.2 in S1 Appendix we discuss the introduction of a set of region-specific parameters controlling the amount of MD error introduced in the early stages of the outbreak. We found that without these parameters the particle trajectories would systematically overestimate the observed curves, despite, in theory, the MD process being able to increase or decrease the numbers of individuals in each state over time. So, what is happening here? The first thing to note is that although the MD has an idealised zero-mean, in order to ensure that the MD adjusted states are epidemiologically valid we require truncation of the MD distributions (see the *Model discrepancy* section). When counts are low, and truncated below by zero, this necessarily means that the mean of the truncated MD process is > 0, which makes epidemiological sense, since the counts can’t be less than zero. However, it also means that in the early stages there is a higher likelihood of the MD increasing the counts rather than decreasing them (i.e., the necessary truncation induces some skewness). As such, in the initial stages particles are more likely to increase on average. The second thing to note is that in the early stages of the outbreak there are negligible control measures in place, and in that situation *R*_0_ is larger than 1 (in fact *R*_0_ > 2 in our model). This means that once seeds are introduced the simulation model will kick in and drive infections upwards unless there is information in the data to put preferential weight on lower counts. This leads to the third consideration, which is that the observed data relate to epidemiological states found towards the *ends* of the various pathways described in Fig 2 (i.e. hospitalisations and deaths). This induces a *lag-effect*, which the standard BPF cannot overcome since it has no way of adjusting states at earlier time points based on data at much later time points. This is a well-known problem in particle filtering, but is acutely felt here because we are trying to track trajectories in high dimensions, in a highly stochastic model with a lag-effect induced by the observable data. We note that this issue is a practical consideration that would be unlikely to happen if we were able to run very large numbers of particles, since there would be a non-negligible probability that at least some of the particles were on trajectories more consistent with later time points.

We deal with this issue in part by introducing the parameters described in the Section C.2 in S1 Appendix, which slows the introduction of MD in different regions. In practice this controlled things better at the regional level (as seen in the various posterior predictive plots such as Figs 3–5), but it is likely that some of these issues were still felt at the LTLA-level, where individual LTLAs were not always mapped precisely, despite the regional-level predictions being good. This could of course be alleviated by having a finer spatial resolution for when the MD process begins to kick in, but this comes at the cost of increasing the size of the parameter space, which is one of the key things we wish to avoid having to do.

All of this motivates future work to explore the use of more sophisticated look-ahead particle filters. A key consideration is that although it is straightforward to simulate forwards from a stochastic compartmental transmission model, it is hard to condition those simulations on observed data without completely rewriting the code base (this is especially challenging given, e.g., stochastic movements of individuals between regions). In simulation work we were able to design a one-step ahead auxiliary particle filter [43] that helped alleviate this problem a bit by simulating the ***X***_*t*_ terms directly from the transmission model, and then using the observed data ***Z***_*t*_ to inform the simulation of the MD adjusted states, ***Y***_*t*_, given ***X***_*t*_ and ***Z***_*t*_. Since the $Y_{t}|X_{t}$ are independent discrete truncated Gaussian random variables, it is easier to build information from ***Z***_*t*_ into the simulation of $Y_{t}|X_{t}$ than it is to build it into the simulation of $X_{t}|Y_{t}$ as would be the usual approach. However, it was challenging to get this working for the real data due to the fact that the observed data were also specified on a different scale to the hidden states (since the one-step ahead filters use the future data to inform simulation of earlier hidden states, this requires a way to map information in observed data at one level of aggregation down to the hidden states at another level of aggregation, which is highly challenging for a model of this complexity). As such, we didn’t progress much further with this here.

Other recent advances in the field of look-ahead filters include twisted particle filters (TPFs; [44]), which are designed explicitly to deal with this exact issue, where information at later time points can be fed back to inform simulations at earlier time points through the introduction of twisting functions that amend the filtering distributions accordingly. These approaches maintain the unbiasedness properties of the likelihood estimate but can reduce the Monte Carlo variance of these estimates hugely. The cost is that the twisting functions have to be learned, and so approaches such as that of [44] iterate through each particle filter multiple times in order to learn these twisting functions appropriately. For a system such as ours, where a single evaluation of a particle filter is highly time consuming, then this could be computationally prohibitive. However, using emulation helps to alleviate some of these computational challenges by reducing the number of filters that have to be evaluated, certainly compared to the alternative of embedding a TPF into an MCMC or SMC^2^ algorithm. By reducing the variance in the likelihood estimates it may be possible to cut out substantially more space at each wave of likelihood calibration, thus requiring less training runs of the model to calibrate effectively. We also think that it is likely that with a good look-ahead filter it would not be necessary to include additional parameters controlling when MD is added, such as those developed in the Section C.2 in S1 Appendix, thus making the calibration challenge far easier.

This is a key area of interest for future research, because an efficient look-ahead filter, coupled with the embedded MD process we have developed, would provide a powerful way to deal with many practical issues associated with calibrating complex spatio-temporal infectious disease models that we have discussed in this paper. We note that key challenges would also have to be overcome in terms of mapping the observed data at low spatial resolutions to the hidden states at high spatial resolutions, as previously discussed for the auxiliary particle filter. Additional methodological work would be required to figure out how to twist the complex transmission model, which is defined in discrete-time and space, and further compounded by the additional MD process. It may be possible to employ similar ideas to those discussed above for the APF, or leverage ideas around conditional sampling similar to those developed in Section J of S1 Appendix. Another interesting approach would be to explore whether it would be possible to target an approximate likelihood surface that can be evaluated using more tractable (but approximate) models [72]. Another area-of-interest would be how to perform *online* inference, since the current approach requires simulating the complete time-series each time one wishes to update the calibration with new data, which is likely to become very time-consuming for long outbreaks.

Overall we think that the broad approach developed here and in Part 1 [17] show great potential in terms of being a generalisable way to calibrate complex infectious disease models, and if efficient look-ahead filtering approaches could be developed then this may well move this into the realms of being able to perform robust inference in real-time.

## Supporting information

S1 Appendix Supporting information and plots. (PDF)

## References

[1] Ferguson NM, Laydon D, Nedjati-Gilani G, Imai N, Ainslie K, Baguelin M, et al. Report 9—Impact of non-pharmaceutical interventions (NPIs) to reduce COVID-19 mortality and healthcare demand. Imperial College; 2020. 10.1007/s11538-020-00726-x PMC7140590 32270376

[2] Keeling MJ, Hill EM, Gorsich EE, Penman B, Guyver-Fletcher G, Holmes A, et al. Predictions of COVID-19 dynamics in the UK: short-term forecasting and analysis of potential exit strategies. PLoS Comput Biol. 2021;17(1):e1008619. doi: 10.1371/journal.pcbi.1008619 33481773 PMC7857604

[3] Birrell P, Blake J, van Leeuwen E, Gent N, De Angelis D. Real-time nowcasting and forecasting of COVID-19 dynamics in England: the first wave. Philos Trans R Soc Lond B Biol Sci. 2021;376(1829):20200279. doi: 10.1098/rstb.2020.0279 34053254 PMC8165585

[4] Danon L, Brooks-Pollock E, Bailey M, Keeling M. A spatial model of COVID-19 transmission in England and Wales: early spread, peak timing and the impact of seasonality. Philos Trans R Soc Lond B Biol Sci. 2021;376(1829):20200272. doi: 10.1098/rstb.2020.0272 34053261 PMC8165591

[5] Hill EM, Atkins BD, Keeling MJ, Dyson L, Tildesley MJ. A network modelling approach to assess non-pharmaceutical disease controls in a worker population: an application to SARS-CoV-2. PLoS Comput Biol. 2021;17(6):e1009058. doi: 10.1371/journal.pcbi.1009058 34133427 PMC8208574

[6] Moore S, Hill EM, Tildesley MJ, Dyson L, Keeling MJ. Vaccination and non-pharmaceutical interventions for COVID-19: a mathematical modelling study. Lancet Infect Dis. 2021;21(6):793–802. doi: 10.1016/S1473-3099(21)00143-2 33743847 PMC7972312

[7] Manley H, Bayley T, Danelian G, Burton L, Finnie T, Charlett A. Combining models to generate consensus medium-term projections of hospital admissions, occupancy and deaths relating to COVID-19 in England. Royal Society Open Science. 2021;1:231832. 10.1098/rsos.231832 PMC11285879 39076350

[8] Silk DS, Bowman VE, Semochkina D, Dalrymple U, Woods DC. Uncertainty quantification for epidemiological forecasts of COVID-19 through combinations of model predictions. Stat Methods Med Res. 2022;31(9):1778–89. doi: 10.1177/09622802221109523 35799481 PMC9272045

[9] Bowman VE. The Good of the Many. 2022. https://ima.org.uk/20358/the-good-of-the-many

[10] Kretzschmar ME, Ashby B, Fearon E, Overton CE, Panovska-Griffiths J, Pellis L, et al. Challenges for modelling interventions for future pandemics. Epidemics. 2022;38:100546. doi: 10.1016/j.epidem.2022.100546 35183834 PMC8830929

[11] King AA, Nguyen D, Ionides EL. Statistical inference for partially observed markov processes via theRPackagepomp. J Stat Soft. 2016;69(12). doi: 10.18637/jss.v069.i12

[12] Widgren S, Bauer P, Eriksson R, Engblom S. SimInf: an R package for data-driven stochastic disease spread simulations. J Stat Soft. 2019;91(12). doi: 10.18637/jss.v091.i12

[13] Woods C, Hedges L, Edsall C, Brooks-Pollock E, Parton-Fenton C, McKinley T, et al. MetaWards: a flexible metapopulation framework for modelling disease spread. JOSS. 2022;7(70):3914. doi: 10.21105/joss.03914

[14] McKinley T, Cook AR, Deardon R. Inference in epidemic models without likelihoods. The International Journal of Biostatistics. 2009;5(1). doi: 10.2202/1557-4679.1171

[15] McKinley TJ, Vernon I, Andrianakis I, McCreesh N, Oakley JE, Nsubuga RN, et al. Approximate Bayesian computation and simulation-based inference for complex stochastic epidemic models. Statist Sci. 2018;33(1). doi: 10.1214/17-sts618

[16] Wilkinson RD. Approximate Bayesian computation (ABC) gives exact results under the assumption of model error. Stat Appl Genet Mol Biol. 2013;12(2):129–41. doi: 10.1515/sagmb-2013-0010 23652634

[17] Williamson DB, McKinley TJ, Xiong X, Salter JM, Challen R, Danon L. On real-time calibrated prediction for complex model-based decision support in pandemics: Part 1. 2025. https://www.medrxiv.org/content/10.1101/2025.05.16.25327688v1 10.1371/journal.pcbi.1014299 PMC13271513 42224360

[18] Kennedy MC, O’Hagan A. Bayesian calibration of computer models. Journal of the Royal Statistical Society Series B: Statistical Methodology. 2001;63(3):425–64. doi: 10.1111/1467-9868.00294

[19] Higdon D, Nakhleh C, Gattiker J, Williams B. A Bayesian calibration approach to the thermal problem. Computer Methods in Applied Mechanics and Engineering. 2008;197(29–32):2431–41. doi: 10.1016/j.cma.2007.05.031

[20] Vernon I, Owen J, Aylett-Bullock J, Cuesta-Lazaro C, Frawley J, Quera-Bofarull A. Bayesian emulation and history matching of JUNE. Philosophical Transactions of the Royal Society A. 2022;380(2233):20220039. 10.1098/rsta.2022.0039 PMC9376712 35965471

[21] Andrianakis I, Vernon IR, McCreesh N, McKinley TJ, Oakley JE, Nsubuga RN, et al. Bayesian history matching of complex infectious disease models using emulation: a tutorial and a case study on HIV in Uganda. PLoS Comput Biol. 2015;11(1):e1003968. doi: 10.1371/journal.pcbi.1003968 25569850 PMC4288726

[22] Andrianakis I, McCreesh N, Vernon I, McKinley TJ, Oakley JE, Nsubuga RN, et al. Efficient History Matching of a High Dimensional Individual-Based HIV Transmission Model. SIAM/ASA J Uncertainty Quantification. 2017;5(1):694–719. doi: 10.1137/16m1093008

[23] Fadikar A, Higdon D, Chen J, Lewis B, Venkatramanan S, Marathe M. Calibrating a stochastic, agent-based model using quantile-based emulation. SIAM/ASA J Uncertainty Quantification. 2018;6(4):1685–706. doi: 10.1137/17m1161233

[24] Dunne M, Mohammadi H, Challenor P, Borgo R, Porphyre T, Vernon I, et al. Complex model calibration through emulation, a worked example for a stochastic epidemic model. Epidemics. 2022;39:100574. doi: 10.1016/j.epidem.2022.100574 35617882 PMC9109972

[25] Hourdin F, Ferster B, Deshayes J, Mignot J, Musat I, Williamson D. Toward machine-assisted tuning avoiding the underestimation of uncertainty in climate change projections. Sci Adv. 2023;9(29):eadf2758. doi: 10.1126/sciadv.adf2758 37467323 PMC10355829

[26] Swallow B, Birrell P, Blake J, Burgman M, Challenor P, Coffeng LE, et al. Challenges in estimation, uncertainty quantification and elicitation for pandemic modelling. Epidemics. 2022;38:100547. doi: 10.1016/j.epidem.2022.100547 35180542 PMC7612598

[27] Aylett-Bullock J, Cuesta-Lazaro C, Quera-Bofarull A, Icaza-Lizaola M, Sedgewick A, Truong H, et al. June: open-source individual-based epidemiology simulation. R Soc Open Sci. 2021;8(7):210506. doi: 10.1098/rsos.210506 34295529 PMC8261230

[28] Gordon NJ, Salmond DJ, Smith AFM. Novel approach to nonlinear/non-Gaussian Bayesian state estimation. IEE Proc F Radar Signal Process UK. 1993;140(2):107. doi: 10.1049/ip-f-2.1993.0015

[29] Doucet A, de Freitas N, Gordon N. Sequential Monte Carlo methods in practice. Springer; 2001.

[30] Doucet A, Johansen AM. A Tutorial on Particle Filtering and Smoothing: Fifteen years later. In: Crisan D, Rozovskii B, editors. The Oxford Handbook of Nonlinear Filtering. Oxford University Press; 2011.

[31] Gilks WR, Berzuini C. Following a moving target—Monte Carlo inference for dynamic Bayesian models. Journal of the Royal Statistical Society Series B: Statistical Methodology. 2001;63(1):127–46. doi: 10.1111/1467-9868.00280

[32] R Core Team. R: A Language and Environment for Statistical Computing. 2022.

[33] Wickham H, Averick M, Bryan J, Chang W, McGowan L, François R, et al. Welcome to the Tidyverse. JOSS. 2019;4(43):1686. doi: 10.21105/joss.01686

[34] Pedersen TL. Patchwork: The Composer of Plots. 2022.

[35] Pebesma E. Simple features for R: standardized support for spatial vector data. The R Journal. 2018;10(1):439–46. doi: 10.32614/RJ-2018-009

[36] Prener C, Revord C. areal: An R package for areal weighted interpolation. JOSS. 2019;4(37):1221. doi: 10.21105/joss.01221

[37] Office for National Statistics, National Records of Scotland, Northern Ireland Statistics and Research Agency. 2011 Census Aggregate Data. UK Data Service. 2016. 10.5257/census/aggregate-2011-1

[38] Danon L, House T, Keeling MJ. The role of routine versus random movements on the spread of disease in Great Britain. Epidemics. 2009;1(4):250–8. doi: 10.1016/j.epidem.2009.11.002 21352771 PMC7185876

[39] Mossong J, Hens N, Jit M, Beutels P, Auranen K, Mikolajczyk R, et al. Social contacts and mixing patterns relevant to the spread of infectious diseases. PLoS Med. 2008;5(3):e74. doi: 10.1371/journal.pmed.0050074 18366252 PMC2270306

[40] Jarvis CI, Van Zandvoort K, Gimma A, Prem K, CMMID COVID-19 working group, Klepac P, et al. Quantifying the impact of physical distance measures on the transmission of COVID-19 in the UK. BMC Med. 2020;18(1):124. doi: 10.1186/s12916-020-01597-8 32375776 PMC7202922

[41] Meakin S, Abbott S, Funk S. NHS trust level Covid-19 data aggregated to a range of spatial scales. 2021. 10.5281/zenodo.4447465

[42] Challen RJ, Griffith GJ, Lacasa L, Tsaneva-Atanasova K. Algorithmic hospital catchment area estimation using label propagation. BMC Health Serv Res. 2022;22(1):828. doi: 10.1186/s12913-022-08127-7 35761225 PMC9235278

[43] Pitt MK, Shephard N. Filtering via simulation: auxiliary particle filters. Journal of the American Statistical Association. 1999;94(446):590–9. doi: 10.1080/01621459.1999.10474153

[44] Guarniero P, Johansen AM, Lee A. The iterated auxiliary particle filter. Journal of the American Statistical Association. 2017;112(520):1636–47. doi: 10.1080/01621459.2016.1222291

[45] Gilks WR, Richardson S, Spiegelhalter DJ. Markov Chain Monte Carlo in Practice. Chapman and Hall; 1996.

[46] Eddelbuettel D, François R. Rcpp: seamless R and C integration. Journal of Statistical Software. 2011;40(8):1–18. doi: 10.18637/jss.v040.i08

[47] Eddelbuettel D, Sanderson C. RcppArmadillo: accelerating R with high-performance C++ linear algebra. Computational Statistics and Data Analysis. 2014;71:1054–63. doi: 10.1016/j.csda.2013.02.005

[48] Ming D, Williamson D, Guillas S. Deep Gaussian process emulation using stochastic imputation. Technometrics. 2023;65(2):150–61.

[49] Ming D, Williamson D. dgpsi: An R package powered by Python for modelling linked deep Gaussian processes. https://CRAN.R-project.org/package=dgpsi

[50] Iskauskas A, Vernon I, Goldstein M, Scarponi D, McCreesh N, McKinley TJ, et al. Emulation and history matching using the hmer package. J Stat Soft. 2024;109(10). doi: 10.18637/jss.v109.i10

[51] Xu W, Williamson DB, Challenor P. local voronoi tessellations for robust multiwave calibration of computer models. Int J UncertaintyQuantification. 2021;11(5):1–17. doi: 10.1615/int.j.uncertaintyquantification.2021034779

[52] Lawrence BN, Bennett VL, Churchill J, Juckes M, Kershaw P, Pascoe S, et al. Storing and manipulating environmental big data with JASMIN. In: 2013 IEEE International Conference on Big Data. 2013. p. 68–75. 10.1109/bigdata.2013.6691556

[53] Oakley JE, Youngman BD. Calibration of stochastic computer simulators using likelihood emulation. Technometrics. 2017;59(1):80–92. doi: 10.1080/00401706.2015.1125391

[54] Wilkinson RD. Proceedings of the 17th International Conference on Artificial Intelligence and Statistics (AISTATS). 2014. p. 1015–23.

[55] Gibson G. Estimating parameters in stochastic compartmental models using Markov chain methods. Mathematical Medicine and Biology. 1998;15(1):19–40. doi: 10.1093/imammb/15.1.19

[56] O’Neill PD, Roberts GO. Bayesian inference for partially observed stochastic epidemics. Journal of the Royal Statistical Society Series A: Statistics in Society. 1999;162(1):121–9. doi: 10.1111/1467-985x.00125

[57] Jewell CP, Kypraios T, Neal P, Roberts GO. Bayesian analysis for emerging infectious diseases. Bayesian Anal. 2009;4(3). doi: 10.1214/09-ba417

[58] Deardon R, Brooks SP, Grenfell BT, Keeling MJ, Tildesley MJ, Savill NJ, et al. inference for individual-level models of infectious diseases in large populations. Stat Sin. 2010;20(1):239–61. 26405426 PMC4578172

[59] Jewell CP, Hale AC, Rowlingson BS, Suter C, Read JM, Roberts G. Bayesian inference for high-dimensional discrete-time epidemic models: spatial dynamics of the UK COVID-19 outbreak. arXiv preprint. 2023. https://arxiv.org/abs/2306.07987

[60] Andrieu C, Roberts GO. The pseudo-marginal approach for efficient Monte Carlo computations. Ann Statist. 2009;37(2). doi: 10.1214/07-aos574

[61] Andrieu C, Doucet A, Holenstein R. Particle Markov Chain Monte Carlo methods. Journal of the Royal Statistical Society Series B: Statistical Methodology. 2010;72(3):269–342. doi: 10.1111/j.1467-9868.2009.00736.x

[62] McKinley TJ, Ross JV, Deardon R, Cook AR. Simulation-based Bayesian inference for epidemic models. Computational Statistics & Data Analysis. 2014;71:434–47. doi: 10.1016/j.csda.2012.12.012

[63] Moral PD, Jasra A, Lee A, Yau C, Zhang X. The alive particle filter and its use in particle Markov Chain Monte Carlo. Stochastic Analysis and Applications. 2015;33(6):943–74. doi: 10.1080/07362994.2015.1060892

[64] Drovandi CC, Pettitt AN, McCutchan RA. Exact and approximate Bayesian inference for low integer-valued time series models with intractable likelihoods. Bayesian Anal. 2016;11(2). doi: 10.1214/15-ba950 26584211

[65] McKinley TJ, Neal P, Spencer SEF, Conlan AJK, Tiley L. Efficient Bayesian model choice for partially observed processes: with application to an experimental transmission study of an infectious disease. Bayesian Anal. 2020;15(3). doi: 10.1214/19-ba1174

[66] Chopin N, Jacob PE, Papaspiliopoulos O. SMC2: an efficient algorithm for sequential analysis of state space models. Journal of the Royal Statistical Society Series B: Statistical Methodology. 2012;75(3):397–426. doi: 10.1111/j.1467-9868.2012.01046.x

[67] Millevoi C, Pasetto D, Ferronato M. A physics-informed neural network approach for compartmental epidemiological models. PLoS Comput Biol. 2024;20(9):e1012387. doi: 10.1371/journal.pcbi.1012387 39236067 PMC11407682

[68] Sainsbury-Dale M, Zammit-Mangion A, Huser R. Likelihood-free parameter estimation with neural Bayes estimators. The American Statistician. 2023;78(1):1–14. doi: 10.1080/00031305.2023.2249522

[69] Vernon I, Goldstein M, Bower RG. Galaxy formation: a Bayesian uncertainty analysis. Bayesian Anal. 2010;5(4). doi: 10.1214/10-ba524

[70] Wu D, Niu R, Chinazzi M, Vespignani A, Ma Y-A, Yu R. Deep Bayesian active learning for accelerating stochastic simulation. In: Proceedings of the 29th ACM SIGKDD Conference on Knowledge Discovery and Data Mining. 2023. p. 2559–69. 10.1145/3580305.3599300

[71] Raynal L, Marin J-M, Pudlo P, Ribatet M, Robert CP, Estoup A. ABC random forests for Bayesian parameter inference. Bioinformatics. 2019;35(10):1720–8. doi: 10.1093/bioinformatics/bty867 30321307

[72] Whiteley N, Rimella L. Inference in stochastic epidemic models via multinomialapproximations. In: International Conference on Artificial Intelligence and Statistics. PMLR; 2021. p. 1297–305.

