# Supplementary material for: On real-time calibrated prediction for complex model-based decision support in pandemics: Part 2
Source: PLoS Comput Biol. 2026 Jun 1;22(6):e1014299. doi: 10.1371/journal.pcbi.1014299 (PMC13271513; doi:10.1371/journal.pcbi.1014299)
Supplement: S1 Appendix — (PDF) [file pcbi.1014299.s001.pdf]

# Supporting Information: On real-time calibrated prediction for complex model-based decision support in pandemics: Part 2

Trevelyan J. McKinley, Daniel B. Williamson, Xiaoyu Xiong, James M. Salter, Robert Challen, Leon Danon, Benjamin D. Youngman and Doug McNeall

## A Packages used

R [R Core Team, 2022] packages used in this work include: `mclust` [Scrucca et al., 2023], `tidyverse` [Wickham et al., 2019], `lubridate` [Grolemund and Wickham, 2011], `GGally` [Schloerke et al., 2024], `truncnorm` [Mersmann et al., 2023], `patchwork` [Pedersen, 2022], `Rcpp` [Eddelbuettel and François, 2011], `RcppArmadillo` [Eddelbuettel and Sanderson, 2014], `abind` [Plate and Heiberger, 2024], `sitmo` [Balamuta et al., 2021], `sf` [Pebesma, 2018], `lhs` [Carnell, 2024], `areal` [Prener and Revord, 2019], `hmer` [Iskauskas et al., 2024], `MASS` [Venables and Ripley, 2002], `fields` [Nychka et al., 2021], `dgpsi` [Ming and Williamson, 2024], `data.table` [Barrett et al., 2025], `NIMBLE` [de Valpine et al., 2017] and `R.utils` [Bengtsson, 2025].

## B Comparative spatial regions

Figure A shows the comparative number and size of regions at different spatial hierarchies in England and Wales in 2019. For the purposes of this expository figure, we have extracted all areas that correspond to the 315 Lower Tier Local Authority (LTLAs) areas with death data available. Figure AA shows the 9 main regions in England and Wales, Figure AB shows the 315 LTLAs with death data available, and the 7,189 electoral wards that map to these LTLAs are shown in Figure AC.

## C Transmission model

Whenever we see forks in the pathways in Figure 2 (of the main text) we have multinomial transitions, otherwise we have binomial transitions. Hence the transitions between states in the period $[t-1, t)$ can be written (using $\cdot$ to denote the number of individuals who *do not transition* in the multinomial draws) as:

$$
\begin{aligned}
\left(X'_{tas,E} \mid \boldsymbol{Y}_{(t-1)as}\right) &\sim \text{Bin}\left(Y_{(t-1)as,S}, q_{(t-1)as,SE}\right), \\
\left(X'_{tas,P}, X'_{tas,A}, \cdot \mid \boldsymbol{Y}_{(t-1)as}\right) &\sim \text{Multinomial}\left(Y_{(t-1)as,E}, \boldsymbol{q}_{a,E}\right), \\
\left(X'_{tas,R_A} \mid \boldsymbol{Y}_{(t-1)as}\right) &\sim \text{Bin}\left(Y_{(t-1)as,A}, 1-e^{-\gamma_A}\right), \\
\left(X'_{tas,I_1} \mid \boldsymbol{Y}_{(t-1)as}\right) &\sim \text{Bin}\left(Y_{(t-1)as,P}, 1-e^{-\gamma_P}\right), \\
\left(X'_{tas,H}, X'_{tas,D_I}, X'_{tas,I_2}, \cdot \mid \boldsymbol{Y}_{(t-1)as}\right) &\sim \text{Multinomial}\left(Y_{(t-1)as,I_1}, \boldsymbol{q}_{a,I_1}\right), \\
\left(X'_{tas,R_I} \mid \boldsymbol{Y}_{(t-1)as}\right) &\sim \text{Bin}\left(Y_{(t-1)as,I_2}, 1-e^{-\gamma_{I_2}}\right), \\
\left(X'_{tas,D_H}, X'_{tas,R_H}, \cdot \mid \boldsymbol{Y}_{(t-1)as}\right) &\sim \text{Multinomial}\left(Y_{(t-1)as,H}, \boldsymbol{q}_{a,H}\right).
\end{aligned}
$$

Here:

$$
\begin{aligned}
\boldsymbol{q}_{a,E} &= \left(p_{a,EP}\left[1-e^{-\gamma_E}\right], \left[1-p_{a,EP}\right]\left[1-e^{-\gamma_E}\right], e^{-\gamma_E}\right), \\
\boldsymbol{q}_{a,I_1} &= \left(p_{a,I_1H}\left[1-e^{-\gamma_{I_1}}\right], p_{a,I_1D}\left[1-e^{-\gamma_{I_1}}\right], \left[1-p_{a,I_1H}-p_{a,I_1D}\right]\left[1-e^{-\gamma_{I_1}}\right], e^{-\gamma_{I_1}}\right), \\
\boldsymbol{q}_{a,H} &= \left(p_{a,HD}\left[1-e^{-\gamma_{a,H}}\right], \left[1-p_{a,HD}\right]\left[1-e^{-\gamma_{a,H}}\right], e^{-\gamma_{a,H}}\right),
\end{aligned}
$$

where

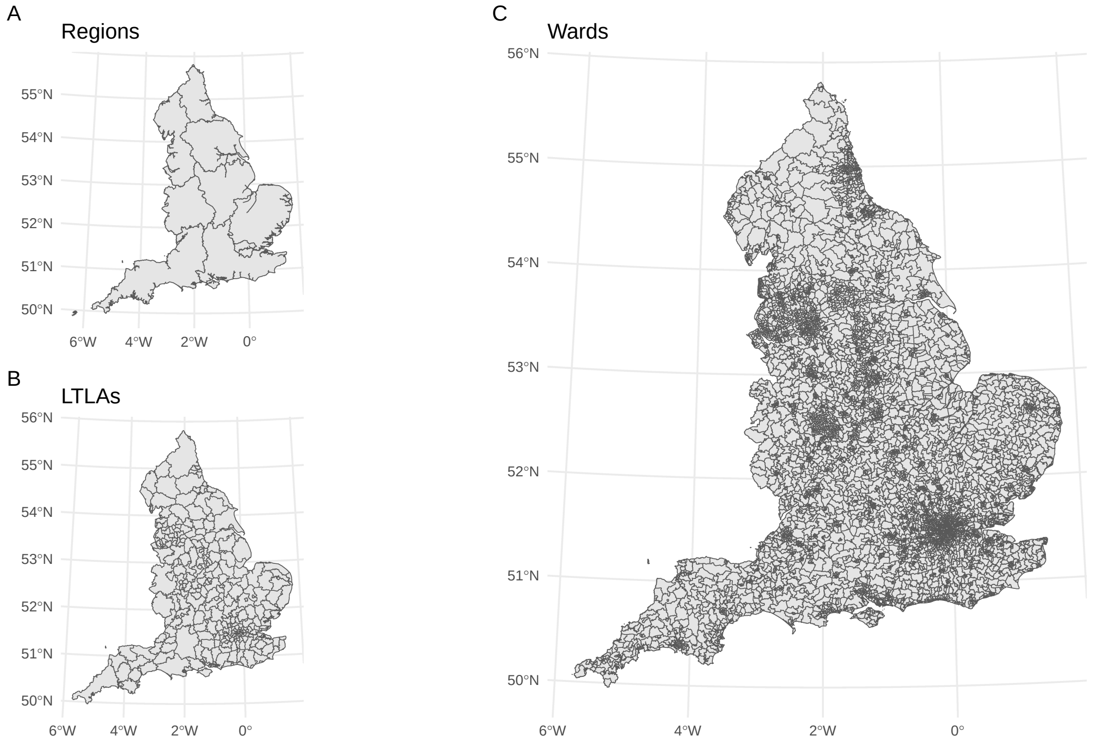


Figure A: Comparative plots of different spatial aggregations. A) Regions (9 shown); B) Lower Tier Local Authority (LTLAs; 315 shown); C) electoral wards (7,189 wards shown). LTLAs shown are those with death data available. Wards shown are those that map to the corresponding LTLAs. Source for shapefiles: https://geoportal.statistics.gov.uk from the Office for National Statistics licensed under the Open Government Licence v.3.0. Contains OS data: Crown copyright and database right 2022.

- $q_{(t-1)as,SE}$ is driven by the number of infectious individuals in age-group $a$, area $s$ at time $t-1$, and a probability of transmission per infectious contact, $\nu\left(R_0\right)$, which is defined as a function of the basic reproduction number, $R_0$, and the next-generation matrix (NGM). These are discussed in more detail in Section C.1 section below.
- The transition rate $\gamma_E = \frac{1}{T_E}$ with $T_E$ the mean latent period.
- The quantity $p_{a,EP}$ is the probability that an individual in age-class $a$ progresses through the *asymptomatic* pathway. We model $\log\left(p_{a,EP}\right) = \alpha_{EP} + \eta \text{ age}_a$, where $\text{age}_a$ is the mid-point of age-class $a$.
- The transition rate $\gamma_A = \frac{1}{T_P+T_{I_1}+T_{I_2}}$ with $T_P + T_{I_1} + T_{I_2}$ the mean infectious period.
- The transition rate $\gamma_P = \frac{1}{T_P}$ with $T_P$ the mean pre-symptomatic infectious period.
- The transition rate $\gamma_{I_1} = \frac{1}{T_{I_1}}$ with $T_{I_1}$ the mean pre-hospitalisation symptomatic infectious period.
- The probability that an individual in age-class $a$ that is currently in the $I_1$ state transitions down the hospitalisation pathway is given by $p_{a,I_1H}$, and the probability that they transition along the death pathway is given by $p_{a,I_1D}$. Similarly to before we model: $\log\left(p_{a,I_1H}\right) = \alpha_{I_1H} + \eta \text{ age}_a$ and $\log\left(p_{a,I_1D}\right) = \alpha_{I_1D} + \eta\ \eta_I \text{ age}_a$, where $\eta_I$ scales the age-relationship for $I_1 \to D_I$ transitions.
- The transition rate $\gamma_{I_2} = \frac{1}{T_{I_2}}$ where $T_{I_2}$ is the additional mean symptomatic infectious period for non-hospitalised individuals.
- The transition rate $\gamma_{a,H} = \frac{1}{T_{a,H}}$ with $T_{a,H}$ the mean length of hospital stay for an individual in age-class $a$. The length of hospital stays is age-specific, and is governed by the model $\log\left(T_{a,H}\right) = \alpha_{T_H} + \eta_{T_H} \text{ age}_a$.
- The probability that an individual in age-class $a$ that is currently in the $H$ state transitions along the death pathway is given by $p_{a,HD}$. Similarly to before we model: $\log\left(p_{a,HD}\right) = \alpha_{HD} + \eta\ \eta_H \text{ age}_a$, where $\eta_H$ scales the age-relationship for $H \to D_H$ transitions.

## C.1 Transmission terms

Transmission is governed by the basic reproduction number, $R_0$, which is defined as the expected number of secondary cases caused by a single infectious case introduced into a fully susceptible population. We also introduce a parameter, $\nu_A$, which allows asymptomatic individuals to have a different transmission potential to symptomatic individuals. The *force-of-infection* (FOI) acting on individuals in age-class $a$ in area $s$ at time $t$, $\lambda_{tas}$, is therefore defined as:

$$\lambda_{tas} = \sum_{k=1}^{N_a} \frac{\beta_{ka}}{M_{tks}} \left(Y_{tks,P} + Y_{tks,I_1} + Y_{tks,I_2} + \nu_A Y_{tks,A}\right) \tag{A.1}$$

where $M_{tks}$ is the population size of age-class $k$ in area $s$ at time $t$. Hence

$$q_{tas,SE} = 1 - e^{-\lambda_{tas}}.$$

We can specify:

$$\beta_{ka} = \nu C_{ka},$$

where $0 < \nu < 1$ is a probability of infection per contact, and $C_{ka}$ is the *population contact rate* that an individual in state $a$ has with individuals in state $k$. We use the population contact matrix $C = \{C_{ka}; k = 1, \ldots, N_a; a = 1, \ldots, N_a\}$ from the POLYMOD survey [Mossong et al., 2008] in the early stages of the outbreak, and then the CoMix survey after the first lockdown [Jarvis et al., 2020].

A key challenge is that since we parameterise our model in terms of $R_0$, not $\nu$, we thus need a way to derive $\nu$ for any given set of parameters. To this end, $R_0$ can be derived as the *maximum eigenvalue* of the NGM [Diekmann et al., 1990, van den Driessche and Watmough, 2002, Diekmann et al., 2010, van den Driessche, 2017], and we can exploit this relationship to derive $\nu$ as a function of $R_0$, which we denote as $\nu(R_0)$ here. For full details please see Section D. We compute $\nu(R_0)$ assuming the NGM defined in a single population of size 56,082,077 (matching the number of people in the commuter data) with 100 initial introductions at time 0. We note that the output is not sensitive to the exact number of susceptibles and infectives at time 0 as long as the number of susceptibles is large and the number of infectives is small. We also note that this mapping of $R_0$ to $\nu$ is based on large population deterministic approximations that ignore the stochastic meta-population dynamics. As such the value of $R_0$ that we calibrate to might not be a perfect representation of the $R_0$ of our actual model, but it gives us a place to start (and it remains unclear how one would calculate $R_0$ for a spatio-temporal model of this kind without making some of these simplifying assumptions).

## C.2 Spatially-varying model discrepancy initiation

We found it was necessary to add in some additional parameters that enabled the MD process to be phased in at different times in different spatial regions to reflect the fact that introductions of infections were not uniform across the country in the early stages of the outbreak. In each region $r$ we allowed the MD parameters to vary over time, such that

$$a_{tr,\text{MD}} = a_{\text{MD}} \exp\left(-\alpha_{\text{MD}} \left[t_{r,\text{MD}} - t\right] \mathbb{1}\left[t < t_{r,\text{MD}}\right]\right), \tag{A.2}$$

where $0 < t_{r,\text{MD}} < t^*$, $0 < \alpha_{\text{MD}} < 1$ for $r = 1, \ldots, N_r$, and

$$\mathbb{1}(x) = \begin{cases} 1 & \text{if } x \text{ true,} \\ 0 & \text{otherwise.} \end{cases}$$

Hence, for $t > t_{r,\text{MD}}$ then $a_{tr,\text{MD}} = a_{\text{MD}}$, but for $t < t_{r,\text{MD}}$ there is an exponential decay the further back in time you go from $t_{r,\text{MD}}$, dependent on some rate parameter $\alpha_{\text{MD}}$. We also set

$$b_{tr,\text{MD}} = b_{\text{MD}} \exp\left(-\alpha_{\text{MD}} \left[t_{r,\text{MD}} - t\right] \mathbb{1}\left[t < t_{r,\text{MD}}\right]\right). \tag{A.3}$$

For all the examples in this paper, we chose $t^*$ to be equal to the time-of-first-lockdown, with $a_{\text{MD}} = 0.05$ and $b_{\text{MD}} = 0.01$. We decided to allow for greater model discrepancy variance within the oldest age-class, to represent additional key sources of uncertainty that were not included in the model, such as transmission in care homes. As such we set $b_{\text{MD}} = 0.5$ for the oldest age-class.

# D Mapping $R_0$ to $\nu$ using the next-generation matrix (NGM)

We refer the reader to e.g. Diekmann et al. [1990], van den Driessche and Watmough [2002], Diekmann et al. [2010], van den Driessche [2017] for more details of the method of constructing NGMs for compartmental models, and using them to derive equations for $R_0$. Here we will use the construction of van den Driessche [2017].

Firstly, let $F_i(x)$ be the rate of *new* infections into *infected* class $i$, and $V_i(x)$ be the rate of transitions from *infected* class $i$. Then define matrices:

$$\mathbf{F} = \left[\frac{\partial F_i\left(x_0\right)}{\partial x_j}\right] \quad \text{and} \quad \mathbf{V} = \left[\frac{\partial V_i\left(x_0\right)}{\partial x_j}\right] \quad \text{for } 1 \leq i, j, \leq N_{\text{inf}},$$

where $x_0$ corresponds to the states at the disease-free equilibrium and $N_{\text{inf}}$ is the number of infected classes. Then, the NGM, $\mathbf{K}$, is:

$$\mathbf{K} = \mathbf{F}\mathbf{V}^{-1},$$

and $R_0$ is the maximum eigenvalue of $\mathbf{K}$.

From the model structure above, in a single population with age-structure, the **infected classes** are $X_{a,E}$, $X_{a,A}$, $X_{a,P}$, $X_{a,I_1}$ and $X_{a,I_2}$ for age-classes $a = 1, \ldots, N_a$. Note that from a *biological* perspective individuals in hospital are also infected, but in our model they play no further role in the transmission process, and so we do not count $X_{a,H}$ as an infected state for the purposes of constructing the NGM. In a *deterministic* model the infected states above would have rates-of-change of:

$$\begin{aligned}
\frac{dX_{a,E}}{dt} &= X_{a,S}\left[\sum_{k=1}^{N_a}\frac{\beta_{ka}}{M_k}\left(X_{k,P} + X_{k,I_1} + X_{k,I_2} + \nu_A X_{k,A}\right)\right] - \gamma_E X_{a,E}\\
\frac{dX_{a,A}}{dt} &= \left(1 - p_{a,EP}\right)\gamma_E X_{a,E} - \gamma_A X_{a,A}\\
\frac{dX_{a,P}}{dt} &= p_{a,EP}\gamma_E X_{a,E} - \gamma_P X_{a,P}\\
\frac{dX_{a,I_1}}{dt} &= \gamma_P X_{a,P} - \gamma_{I_1} X_{a,I_1}\\
\frac{dX_{a,I_2}}{dt} &= \left(1 - p_{a,I_1H} - p_{a,I_1D}\right)\gamma_{I_1} X_{a,I_1} - \gamma_{I_2} X_{a,I_2}
\end{aligned}$$

Here $\beta_{ka}$ is the transmission rate from class $k$ to class $a$ and $M_k$ is the total number of individuals in class $k$. The parameter $\nu_A \in (0, 1)$ scales the force-of-infection from the $A$ class relative to the other infectious classes.

Letting $i = 1, \ldots, N_a$ and $j = 1, \ldots, N_a$, then for brevity we only show the non-zero components needed to derive the NGM, which are:

$$\begin{aligned}
\frac{\partial F_{X_{i,E}}\left(x_0\right)}{\partial X_{j,A}} &= \frac{\beta_{ji}\nu_A X_{i,S}}{M_j} && \forall i, j\\
\frac{\partial F_{X_{i,E}}\left(x_0\right)}{\partial X_{j,P}} &= \frac{\beta_{ji} X_{i,S}}{M_j} && \forall i, j\\
\frac{\partial F_{X_{i,E}}\left(x_0\right)}{\partial X_{j,I_1}} &= \frac{\beta_{ji} X_{i,S}}{M_j} && \forall i, j\\
\frac{\partial F_{X_{i,E}}\left(x_0\right)}{\partial X_{j,I_2}} &= \frac{\beta_{ji} X_{i,S}}{M_j} && \forall i, j
\end{aligned}$$

and

$$\frac{\partial V_{X_{i,E}}(x_0)}{\partial X_{j,E}} = \begin{cases} \gamma_E & \text{for } i=j, \\ 0 & \text{otherwise,} \end{cases}$$
$$\frac{\partial V_{X_{i,A}}(x_0)}{\partial X_{j,E}} = \begin{cases} -(1-p_{i,EP})\gamma_E & \text{for } i=j, \\ 0 & \text{otherwise,} \end{cases}$$
$$\frac{\partial V_{X_{i,A}}(x_0)}{\partial X_{j,A}} = \begin{cases} \gamma_A & \text{for } i=j, \\ 0 & \text{otherwise,} \end{cases}$$
$$\frac{\partial V_{X_{i,P}}(x_0)}{\partial X_{j,E}} = \begin{cases} -p_{i,EP}\gamma_E & \text{for } i=j, \\ 0 & \text{otherwise,} \end{cases}$$
$$\frac{\partial V_{X_{i,P}}(x_0)}{\partial X_{j,P}} = \begin{cases} \gamma_P & \text{for } i=j, \\ 0 & \text{otherwise,} \end{cases}$$
$$\frac{\partial V_{X_{i,I_1}}(x_0)}{\partial X_{j,P}} = \begin{cases} -\gamma_P & \text{for } i=j, \\ 0 & \text{otherwise,} \end{cases}$$
$$\frac{\partial V_{X_{i,I_1}}(x_0)}{\partial X_{j,I_1}} = \begin{cases} \gamma_{I_1} & \text{for } i=j, \\ 0 & \text{otherwise,} \end{cases}$$
$$\frac{\partial V_{X_{i,I_2}}(x_0)}{\partial X_{j,I_1}} = \begin{cases} -(1-p_{i,I_1H}-p_{i,I_1D})\gamma_{I_1} & \text{for } i=j, \\ 0 & \text{otherwise,} \end{cases}$$
$$\frac{\partial V_{X_{i,I_2}}(x_0)}{\partial X_{j,I_2}} = \begin{cases} \gamma_{I_2} & \text{for } i=j, \\ 0 & \text{otherwise.} \end{cases}$$

Since the non-zero components of $\mathbf{F}$ all contain $\beta_{ji}$, where

$$\beta_{ji} = \nu c_{ji},$$

we can therefore write

$$\mathbf{K} = \nu \mathbf{G}\mathbf{V}^{-1}$$

where $\mathbf{G}$ is equivalent to replacing $\beta_{ji}$ by $c_{ji}$ in $\mathbf{F}$. As such:

$$\begin{aligned} R_0 &= \nu \ \mathrm{eig}_M\left(\mathbf{G}\mathbf{V}^{-1}\right) \\ \Rightarrow \nu &= \frac{R_0}{\mathrm{eig}_M\left(\mathbf{G}\mathbf{V}^{-1}\right)} \end{aligned}$$

where $\mathrm{eig}_M(\mathbf{K})$ denotes the maximum eigenvalue of $\mathbf{K}$.

When using a mixture of contact matrices, we parameterise $\nu$ using the NGM evaluated at the initial contact structure, which represents the contact matrix expected in a completely susceptible population at the start of the outbreak.

# E Model discrepancy constraints and updates

Starting with the absorbing states we have:

$$\begin{aligned} \left(\Delta'_{tas,D_H} \mid \boldsymbol{X}_{tas}, \mathbf{Y}_{(t-1)as}\right) &\sim N^D\left(0, \sigma^2_{tas,D_H}\right) I\left(-X'_{tas,D_H}, Y_{(t-1)as,H} - X'_{tas,D_H}\right), \\ \sigma^2_{tas,D_H} &= 2a_{\mathrm{MD}} + 2b_{\mathrm{MD}} X'_{tas,D_H}, \\ Y'_{tas,D_H} &= X'_{tas,D_H} + \Delta'_{tas,D_H}, \\ Y_{tas,D_H} &= X_{tas,D_H} + \Delta'_{tas,D_H}. \end{aligned} \quad (A.4)$$

$$\begin{aligned} \left(\Delta'_{tas,D_I} \mid \boldsymbol{X}_{tas}, \mathbf{Y}_{(t-1)as}\right) &\sim N^D\left(0, \sigma^2_{tas,D_I}\right) I\left(-X'_{tas,D_I}, Y_{(t-1)as,I_1} - X'_{tas,D_I}\right), \\ \sigma^2_{tas,D_I} &= 2a_{\mathrm{MD}} + 2b_{\mathrm{MD}} X'_{tas,D_I}, \\ Y'_{tas,D_I} &= X'_{tas,D_I} + \Delta'_{tas,D_I}, \\ Y_{tas,D_I} &= X_{tas,D_I} + \Delta'_{tas,D_I}. \end{aligned} \quad (A.5)$$

$$
\begin{aligned}
\left(\Delta'_{tas,R_H} \mid Y'_{tas,D_H}, \boldsymbol{X}_{tas}, \boldsymbol{Y}_{(t-1)as}\right) \sim N^D\left(0, \sigma^2_{tas,R_H}\right) I\left(-X'_{tas,R_H}, Y_{(t-1)as,H} - Y'_{tas,D_H} - X'_{tas,R_H}\right),\\
\sigma^2_{tas,R_H} &= 2a_{\mathrm{MD}} + 2b_{\mathrm{MD}} X'_{tas,R_H},\\
Y'_{tas,R_H} &= X'_{tas,R_H} + \Delta'_{tas,R_H},\\
Y_{tas,R_H} &= X_{tas,R_H} + \Delta'_{tas,R_H}.
\end{aligned}
\tag{A.6}
$$

$$
\begin{aligned}
\left(\Delta'_{tas,R_I} \mid \boldsymbol{X}_{tas}, \boldsymbol{Y}_{(t-1)as}\right) \sim N^D\left(0, \sigma^2_{tas,R_I}\right) I\left(-X'_{tas,R_I}, Y_{(t-1)as,I_2} - X'_{tas,R_I}\right),\\
\sigma^2_{tas,R_I} &= 2a_{\mathrm{MD}} + 2b_{\mathrm{MD}} X'_{tas,R_I},\\
Y'_{tas,R_I} &= X'_{tas,R_I} + \Delta'_{tas,R_I},\\
Y_{tas,R_I} &= X_{tas,R_I} + \Delta'_{tas,R_I}.
\end{aligned}
\tag{A.7}
$$

$$
\begin{aligned}
\left(\Delta'_{tas,R_A} \mid \boldsymbol{X}_{tas}, \boldsymbol{Y}_{(t-1)as}\right) \sim N^D\left(0, \sigma^2_{tas,R_A}\right) I\left(-X'_{tas,R_A}, Y_{(t-1)as,A} - X'_{tas,R_A}\right),\\
\sigma^2_{tas,R_A} &= 2a_{\mathrm{MD}} + 2b_{\mathrm{MD}} X'_{tas,R_A},\\
Y'_{tas,R_A} &= X'_{tas,R_A} + \Delta'_{tas,R_A},\\
Y_{tas,R_A} &= X_{tas,R_A} + \Delta'_{tas,R_A}.
\end{aligned}
\tag{A.8}
$$

Now moving on to the non-absorbing states, we have:

$$
\begin{aligned}
\left(\Delta_{tas,H} \mid, Y_{tas,D_H}, Y_{tas,R_H}, \boldsymbol{X}_{tas}, \boldsymbol{Y}_{(t-1)as}\right) \sim N^D\left(0, \sigma^2_{tas,H}\right)\\
I\left(-X_{tas,H} + Y_{(t-1)as,H} - Y'_{tas,D_H} - Y'_{tas,R_H},\right.\\
Y_{(t-1)as,I_1} - Y'_{tas,D_I} - X_{tas,H} + Y_{(t-1)as,H}\\
\left. - Y'_{tas,D_H} - Y'_{tas,R_H}\right),\\
\sigma^2_{tas,H} &= 2a_{\mathrm{MD}} + 2b_{\mathrm{MD}} X_{tas,H},\\
Y_{tas,H} &= X_{tas,H} + \Delta_{tas,H},\\
Y'_{tas,H} &= X_{tas,H} + \Delta_{tas,H} - Y_{(t-1)as,H} + Y'_{tas,D_H} + Y'_{tas,R_H}.
\end{aligned}
\tag{A.9}
$$

$$
\begin{aligned}
\left(\Delta_{tas,I_2} \mid Y'_{tas,H}, Y'_{tas,D_I}, Y'_{tas,R_I}, \boldsymbol{X}_{tas}, \boldsymbol{Y}_{(t-1)as}\right) \sim N^D\left(0, \sigma^2_{tas,I_2}\right)\\
I\left(-X_{tas,I_2} + Y_{(t-1)as,I_2} - Y'_{tas,R_I},\right.\\
Y_{(t-1)as,I_1} - Y'_{tas,D_I} - Y'_{tas,H}\\
\left. - X_{tas,I_2} + Y_{(t-1)as,I_2} - Y'_{tas,R_I}\right),\\
\sigma^2_{tas,I_2} &= 2a_{\mathrm{MD}} + 2b_{\mathrm{MD}} X_{tas,I_2},\\
Y_{tas,I_2} &= X_{tas,I_2} + \Delta_{tas,I_2},\\
Y'_{tas,I_2} &= X_{tas,I_2} + \Delta_{tas,I_2} - Y_{(t-1)as,I_2} + Y'_{tas,R_I}.
\end{aligned}
\tag{A.10}
$$

$$
\begin{aligned}
\left(\Delta_{tas,I_1} \mid Y'_{tas,I_2}, Y'_{tas,D_I}, Y'_{tas,H}, \boldsymbol{X}_{tas}, \boldsymbol{Y}_{(t-1)as}\right) \sim N^D\left(0, \sigma^2_{tas,I_1}\right)\\
I\left(-X_{tas,I_1} + Y_{(t-1)as,I_1}\right.\\
- Y'_{tas,I_2} - Y'_{tas,D_I} - Y'_{tas,H},\\
Y_{(t-1)as,P} - X_{tas,I_1} + Y_{(t-1)as,I_1} - Y'_{tas,I_2}\\
\left. - Y'_{tas,D_I} - Y'_{tas,H}\right),\\
\sigma^2_{tas,I_1} &= 2a_{\mathrm{MD}} + 2b_{\mathrm{MD}} X_{tas,I_1},\\
Y_{tas,I_1} &= X_{tas,I_1} + \Delta_{tas,I_1},\\
Y'_{tas,I_1} &= X_{tas,I_1} + \Delta_{tas,I_1} - Y_{(t-1)as,I_1} + Y'_{tas,I_2}\\
&\quad + Y'_{tas,D_I} + Y'_{tas,H}.
\end{aligned}
\tag{A.11}
$$

$$
\begin{aligned}
\left(\Delta_{tas,P} \mid Y'_{tas,I_1}, \boldsymbol{X}_{tas}, \boldsymbol{Y}_{(t-1)as}\right) \sim N^D\left(0, \sigma^2_{tas,P}\right) &\\
I\left(-X_{tas,P} + Y_{(t-1)as,P} - Y'_{tas,I_1},\right. &\\
\left. Y_{(t-1)as,E} - X_{tas,P} + Y_{(t-1)as,P} - Y'_{tas,I_1}\right), &\\
\sigma^2_{tas,P} = 2a_{\text{MD}} + 2b_{\text{MD}} X_{tas,P}, &\\
Y_{tas,P} = X_{tas,P} + \Delta_{tas,P}, &\\
Y'_{tas,P} = X_{tas,P} + \Delta_{tas,P} - Y_{(t-1)as,P} + Y'_{tas,I_1}. &
\end{aligned} \tag{A.12}
$$

$$
\begin{aligned}
\left(\Delta_{tas,A} \mid Y'_{tas,R_A}, Y'_{tas,P}, \boldsymbol{X}_{tas}, \boldsymbol{Y}_{(t-1)as}\right) \sim N^D\left(0, \sigma^2_{tas,A}\right) &\\
I\left(-X_{tas,A} + Y_{(t-1)as,A} - Y'_{tas,R_A},\right. &\\
Y_{(t-1)as,E} - Y'_{tas,P} - X_{tas,A} &\\
\left. + Y_{(t-1)as,A} - Y'_{tas,R_A}\right), &\\
\sigma^2_{tas,A} = 2a_{\text{MD}} + 2b_{\text{MD}} X_{tas,A}, &\\
Y_{tas,A} = X_{tas,A} + \Delta_{tas,A}, &\\
Y'_{tas,A} = X_{tas,A} + \Delta_{tas,A} - Y_{(t-1)as,A} + Y'_{tas,R_A}. &
\end{aligned} \tag{A.13}
$$

$$
\begin{aligned}
\left(\Delta_{tas,E} \mid Y'_{tas,P}, Y'_{tas,A}, \boldsymbol{X}_{tas}, \boldsymbol{Y}_{(t-1)as}\right) \sim N^D\left(0, \sigma^2_{tas,E}\right) &\\
I\left(-X_{tas,E} + Y_{(t-1)as,E} - Y'_{tas,P} - Y'_{tas,A},\right. &\\
\left. Y_{(t-1)as,S} - X_{tas,E} + Y_{(t-1)as,E} - Y'_{tas,P} - Y'_{tas,A}\right), &\\
\sigma^2_{tas,E} = 2a_{\text{MD}} + 2b_{\text{MD}} X_{tas,E}, &\\
Y_{tas,E} = X_{tas,E} + \Delta_{tas,E}, &\\
Y'_{tas,E} = X_{tas,E} + \Delta_{tas,E} - Y_{(t-1)as,E} + Y'_{tas,P} + Y'_{tas,A} &\\
Y_{tas,S} = Y_{(t-1)as,S} - Y'_{tas,E}. &
\end{aligned} \tag{A.14}
$$

Since the model discrepancy terms are assumed conditionally independent within each age-class and region, for brevity we drop the *as* subscripts in the derivations below. Note that in all derivations we leverage the fact that all counts must be $\geq 0$. The parameters of each MD distribution are given in the *Model discrepancy* section in the main text.

## E.1 Full derivations of bounds

The *Factorising the joint discrepancy* section in the main text of Part 1 [Williamson et al., 2025] describes a generic mechanism for deriving appropriate bounds on the embedded model discrepancy process for compartmental models. For illustration, here we follow that process through explicitly for all compartments in our model.

Starting with the absorbing states, consider first $Y_{t,D_H}$. We have that $Y_{t,D_H} = Y_{t-1,D_H} + Y'_{t,D_H}$, and from the model structure we can derive bounds

$$0 \leq Y'_{t,D_H} \leq Y_{t-1,H}.$$

Since the adjusted incidence $Y'_{t,D_H} = X'_{t,D_H} + \Delta'_{t,D_H}$, we can derive constraints on $\Delta'_{t,D_H}$ such that

$$-X'_{t,D_H} \leq \Delta'_{t,D_H} \leq Y_{t-1,H} - X'_{t,D_H}.$$

The adjusted count is therefore given by

$$Y_{t,D_H} = X_{t,D_H} + \Delta'_{t,D_H},$$

since

$$
\begin{aligned}
Y_{t,D_H} &= Y_{t-1,D_H} + Y'_{t,D_H}, \\
&= Y_{t-1,D_H} + X'_{t,D_H} + \Delta'_{t,D_H}, \\
&= X_{t,D_H} + \Delta'_{t,D_H} \qquad \text{since } X_{t,D_H} = Y_{t-1,D_H} + X'_{t,D_H}.
\end{aligned}
$$

This leads to the the form given in (A.4).

Now consider $Y_{t,D_I}$. From the model structure we can derive bounds for the MD as:

$$\begin{aligned} 0 \leq Y'_{t,D_I} &\leq Y_{t-1,I_1}, \\ 0 \leq X'_{t,D_I} + \Delta'_{t,D_I} &\leq Y_{t-1,I_1}, \\ -X'_{t,D_I} \leq \Delta'_{t,D_I} &\leq Y_{t-1,I_1} - X'_{t,D_I}. \end{aligned}$$

The adjusted counts are derived similarly to $Y_{t,D_H}$, leading to the the form given in (A.5).

Now consider $Y_{t,R_H}$. From the model structure we know that

$$\begin{aligned} Y'_{t,D_H} + Y'_{t,R_H} &\leq Y_{t-1,H}, \\ \Rightarrow 0 \leq Y'_{t,R_H} &\leq Y_{t-1,H} - Y'_{t,D_H}, \end{aligned}$$

and since $Y'_{t,R_H} = X'_{t,R_H} + \Delta'_{t,R_H}$ we can derive a constraint for $\Delta'_{t,R_H}$ conditional on the adjusted $Y'_{t,D_H}$, such that

$$-X'_{t,R_H} \leq \Delta'_{t,R_H} \leq Y_{t-1,H} - Y'_{t,D_H} - X'_{t,R_H}.$$

The adjusted counts are derived similarly to above, leading to the the form given in (A.6).

Now consider $Y_{t,R_I}$. From the model structure we can derive bounds for the MD as:

$$\begin{aligned} 0 \leq Y'_{t,R_I} &\leq Y_{t-1,I_2}, \\ 0 \leq X'_{t,R_I} + \Delta'_{t,R_I} &\leq Y_{t-1,I_2}, \\ -X'_{t,R_I} \leq \Delta'_{t,R_I} &\leq Y_{t-1,I_2} - X'_{t,R_I}. \end{aligned}$$

The adjusted counts are derived similarly to above, leading to the the form given in (A.7).

Now consider $Y_{t,R_A}$. From the model structure we can derive bounds for the MD as:

$$\begin{aligned} 0 \leq Y'_{t,R_A} &\leq Y_{t-1,A}, \\ 0 \leq X'_{t,R_A} + \Delta'_{t,R_A} &\leq Y_{t-1,A}, \\ -X'_{t,R_A} \leq \Delta'_{t,R_A} &\leq Y_{t-1,A} - X'_{t,R_A}. \end{aligned}$$

The adjusted counts are derived similarly to above, leading to the the form given in (A.8).

Now consider the non-absorbing states, starting with $Y_{t,H}$. We know from the model structure that

$$Y_{t,H} = Y_{t-1,H} + Y'_{t,H} - Y'_{t,D_H} - Y'_{t,R_H},$$

and we now place the model discrepancy on the state and *not* the incidence. Therefore,

$$Y_{t,H} = X_{t,H} + \Delta_{t,H}.$$

Hence

$$\begin{aligned} Y_{t,H} &= Y_{t-1,H} + Y'_{t,H} - Y'_{t,D_H} - Y'_{t,R_H} \\ \Rightarrow Y'_{t,H} &= Y_{t,H} - Y_{t-1,H} + Y'_{t,D_H} + Y'_{t,R_H} \\ \Rightarrow Y'_{t,H} &= X_{t,H} + \Delta_{t,H} - Y_{t-1,H} + Y'_{t,D_H} + Y'_{t,R_H}. \end{aligned}$$

Conditional on the adjusted counts simulated so far, we have that $0 \leq Y'_{t,H} \leq Y_{t-1,I_1} - Y'_{t,D_I}$, and as such we can derive bounds:

$$\begin{aligned} 0 \leq X_{t,H} + \Delta_{t,H} - Y_{t-1,H} + Y'_{t,D_H} + Y'_{t,R_H} &\leq Y_{t-1,I_1} - Y'_{t,D_I} \\ \Rightarrow -X_{t,H} + Y_{t-1,H} - Y'_{t,D_H} - Y'_{t,R_H} \leq \Delta_{t,H} &\leq Y_{t-1,I_1} - Y'_{t,D_I} - X_{t,H} + Y_{t-1,H} - Y'_{t,D_H} - Y'_{t,R_H}, \end{aligned}$$

leading to the the form given in (A.9).

Now consider $Y_{t,I_2}$. From the model structure we have that

$$Y_{t,I_2} = Y_{t-1,I_2} + Y'_{t,I_2} - Y'_{t,R_I},$$

and we place MD on the *counts*, such that:

$$Y_{t,I_2} = X_{t,I_2} + \Delta_{t,I_2}.$$

Hence:

$$\begin{aligned} Y_{t,I_2} &= Y_{t-1,I_2} + Y'_{t,I_2} - Y'_{t,R_I} \\ \Rightarrow Y'_{t,I_2} &= Y_{t,I_2} - Y_{t-1,I_2} + Y'_{t,R_I} \\ \Rightarrow Y'_{t,I_2} &= X_{t,I_2} + \Delta_{t,I_2} - Y_{t-1,I_2} + Y'_{t,R_I} \end{aligned}$$

Conditional on the adjusted counts simulated so far, we have that $0 \leq Y'_{t,I_2} \leq Y_{t-1,I_1} - Y'_{t,D_I} - Y'_{t,H}$, and as such we can derive bounds:

$$\begin{aligned} 0 \leq Y'_{t,I_2} &\leq Y_{t-1,I_1} - Y'_{t,D_I} - Y'_{t,H} \\ \Rightarrow 0 \leq X_{t,I_2} + \Delta_{t,I_2} - Y_{t-1,I_2} + Y'_{t,R_I} &\leq Y_{t-1,I_1} - Y'_{t,D_I} - Y'_{t,H} \\ \Rightarrow -X_{t,I_2} + Y_{t-1,I_2} - Y'_{t,R_I} \leq \Delta_{t,I_2} &\leq Y_{t-1,I_1} - Y'_{t,D_I} - Y'_{t,H} - X_{t,I_2} + Y_{t-1,I_2} - Y'_{t,R_I}, \end{aligned}$$

leading to the the form given in (A.10).

Now consider $Y_{t,I_1}$. From the model structure we have that

$$Y_{t,I_1} = Y_{t-1,I_1} + Y'_{t,I_1} - Y'_{t,I_2} - Y'_{t,D_I} - Y'_{t,H},$$

and we place MD on the *counts*, such that:

$$Y_{t,I_1} = X_{t,I_1} + \Delta_{t,I_1}$$

Hence:

$$\begin{aligned} Y_{t,I_1} &= Y_{t-1,I_1} + Y'_{t,I_1} - Y'_{t,I_2} - Y'_{t,D_I} - Y'_{t,H} \\ Y'_{t,I_1} &= Y_{t,I_1} - Y_{t-1,I_1} + Y'_{t,I_2} + Y'_{t,D_I} + Y'_{t,H} \end{aligned}$$

and since $0 \leq Y'_{t,I_1} \leq Y_{t-1,P}$ we can derive bounds:

$$\begin{aligned} & 0 \leq Y'_{t,I_1} \leq Y_{t-1,P}, \\ & 0 \leq Y_{t,I_1} - Y_{t-1,I_1} + Y'_{t,I_2} + Y'_{t,D_I} + Y'_{t,H} \leq Y_{t-1,P}, \\ & 0 \leq X_{t,I_1} + \Delta_{t,I_1} - Y_{t-1,I_1} + Y'_{t,I_2} + Y'_{t,D_I} + Y'_{t,H} \leq Y_{t-1,P} \\ -X_{t,I_1} + Y_{t-1,I_1} - Y'_{t,I_2} - Y'_{t,D_I} - Y'_{t,H} \leq \Delta_{t,I_1} & \leq Y_{t-1,P} - X_{t,I_1} + Y_{t-1,I_1} - Y'_{t,I_2} - Y'_{t,D_I} - Y'_{t,H}, \end{aligned}$$

leading to the the form given in (A.11).

Now consider $Y_{t,P}$. From the model structure we have that

$$Y_{t,P} = Y_{t-1,P} + Y'_{t,P} - Y'_{t,I_1}$$

with MD placed on the *counts* as

$$Y_{t,P} = X_{t,P} + \Delta_{t,P}.$$

Since $0 \leq Y'_{t,P} \leq Y_{t-1,E}$, we can derive bounds

$$\begin{aligned} & 0 \leq Y'_{t,P} \leq Y_{t-1,E}, \\ & 0 \leq Y_{t,P} - Y_{t-1,P} + Y'_{t,I_1} \leq Y_{t-1,E}, \\ & 0 \leq X_{t,P} + \Delta_{t,P} - Y_{t-1,P} + Y'_{t,I_1} \leq Y_{t-1,E}, \\ -X_{t,P} + Y_{t-1,P} - Y'_{t,I_1} \leq \Delta_{t,P} & \leq Y_{t-1,E} - X_{t,P} + Y_{t-1,P} - Y'_{t,I_1}, \end{aligned}$$

leading to the the form given in (A.12).

Now consider $Y_{t,A}$. From the model structure we have that

$$Y_{t,A} = Y_{t-1,A} + Y'_{t,A} - Y'_{t,R_A}$$

and place MD on the *counts*, such that

$$Y_{t,A} = X_{t,A} + \Delta_{t,A}$$

Conditional on the adjusted counts simulated so far, we have that $0 \leq Y'_{t,A} \leq Y_{t-1,E} - Y'_{t,P}$, and thus we can derive bounds:

$$\begin{aligned}
0 &\leq Y'_{t,A} \leq Y_{t-1,E} - Y'_{t,P} \\
0 &\leq Y_{t,A} - Y_{t-1,A} + Y'_{t,R_A} \leq Y_{t-1,E} - Y'_{t,P} \\
0 &\leq X_{t,A} + \Delta_{t,A} - Y_{t-1,A} + Y'_{t,R_A} \leq Y_{t-1,E} - Y'_{t,P} \\
-X_{t,A} + Y_{t-1,A} - Y'_{t,R_A} &\leq \Delta_{t,A} \leq Y_{t-1,E} - Y'_{t,P} - X_{t,A} + Y_{t-1,A} - Y'_{t,R_A}
\end{aligned}$$

leading to the the form given in (A.13).

Now consider $Y_{t,E}$. From the model structure we have that

$$Y_{t,E} = Y_{t-1,E} + Y'_{t,E} - Y'_{t,P} - Y'_{t,A}$$

and we place MD on the *count*, such that

$$Y_{t,E} = X_{t,E} + \Delta_{t,E}$$

Since $0 \leq Y'_{t,E} \leq Y_{t-1,S}$ we can derive bounds:

$$\begin{aligned}
0 &\leq Y'_{t,E} \leq Y_{t-1,S}, \\
0 &\leq Y_{t,E} - Y_{t-1,E} + Y'_{t,P} + Y'_{t,A} \leq Y_{t-1,S}, \\
0 &\leq X_{t,E} + \Delta_{t,E} - Y_{t-1,E} + Y'_{t,P} + Y'_{t,A} \leq Y_{t-1,S} \\
-X_{t,E} + Y_{t-1,E} - Y'_{t,P} - Y'_{t,A} &\leq \Delta_{t,E} \leq Y_{t-1,S} - X_{t,E} + Y_{t-1,E} - Y'_{t,P} - Y'_{t,A},
\end{aligned}$$

leading to the the form given in (A.14). Finally we have

$$Y_{t,S} = Y_{t-1,S} - Y'_{t,E}.$$

# F Redistribution of events across movement cohorts

Model discrepancy and the infection process is applied at the LTLA-level, not the movement cohort level. Furthermore, we apply the model discrepancy within each LTLA at the end of the day when all individuals are in their home LTLAs. However, since movements are governed by movement cohorts, we require a mechanism to redistribute the model discrepancy adjustments and infections (both sampled at the LTLA level) across the relevant movement cohorts. We do this proportionately to the numbers of affected transitions across all movement cohorts within each LTLA. In the following discussion, let $\mathcal{M}_{tas}$ denote the set of movement cohorts at time $t$ in age-class $a$ that are nested in spatial area $s$. With a slight abuse of notation, we use the specific subscripts ($s$ and $m$) to denote the aggregation level of the observation when referring to the spatial component. Since we simulate at the movement cohort level we also have access to $\boldsymbol{X}_{tam}$ for all $m \in \mathcal{M}_{tas}$ resulting from the simulator.

As an example of the redistribution process, consider that we wish to apply model discrepancy to hospital death incidence. Hence, once we have sampled $\Delta'_{tas,D_H}$ according to the process described in the *Model discrepancy* section of the main text and Section E, then if $\Delta'_{tas,D_H} > 0$ we must move additional individuals from the $H$ state to the $D_H$ state, whereas if $\Delta'_{tas,D_H} < 0$ we will instead be reversing some of the $H \to D_H$ moves that have already occurred. An example algorithm for redistributing $\Delta'_{tas,D_H}$ across movement cohorts $m \in \mathcal{M}_{tas}$ is given in Algorithm A. Similar algorithms can be derived for the other states in the model, but for brevity we have not shown these here.

**Algorithm A** Redistribution of $\Delta'_{tas,D_H}$ at time $t$ for age-class $a$ in spatial area $s$ across movement cohorts $m \in \mathcal{M}_{tas}$.

**Require:** For spatial area $s$ at time $t$ and age-class $a$, we have a sampled model discrepancy for the $D_H$ incidence, $\Delta'_{tas,D_H}$, the set of movement cohorts in $s$ ($\mathcal{M}_{tas}$) and the results of the simulator, $X_{tam,H}$, $X_{tam,D_H}$ and $X'_{tam,D_H}$ for each movement cohort $m \in \mathcal{M}_{tas}$.

**for** $m \in \mathcal{M}_{tas}$ **do**
    **if** $\nexists\ Y_{tam,H}$ **then**
        Set $Y_{tam,H} = X_{tam,H}$.
    **end if**
    **if** $\nexists\ Y_{tam,D_H}$ **then**
        Set $Y_{tam,D_H} = X_{tam,D_H}$.
    **end if**
    **if** $\nexists\ Y'_{tam,D_H}$ **then**
        Set $Y'_{tam,D_H} = X'_{tam,D_H}$.
    **end if**
**end for**
Set $r = \Delta'_{tas,D_H}$.
**while** $r > 0$ **do**
    **for** $m \in \mathcal{M}_{tas}$ **do**
        Calculate $p_{tam,H} = \frac{Y_{tam,H}}{\sum_{n \in \mathcal{M}_{tas}} Y_{tan,H}}$.
    **end for**
    Sample $m' \in \mathcal{M}_{tas}$ according to probabilities $\boldsymbol{p}_{ta\cdot,H}$.
    Set $Y_{tam',H} = Y_{tam',H} - 1$.
    Set $Y_{tam',D_H} = Y_{tam',D_H} + 1$.
    Set $Y'_{tas,D_H} = Y_{tas,D_H} + 1$.
    Set $r = r - 1$.
**end while**
**while** $r < 0$ **do**
    **for** $m \in \mathcal{M}_{tas}$ **do**
        Calculate $p'_{tam,D_H} = \frac{Y'_{tam,D_H}}{\sum_{n \in \mathcal{M}_{tas}} Y'_{tan,D_H}}$.
    **end for**
    Sample $m' \in \mathcal{M}_{tas}$ according to probabilities $\boldsymbol{p}'_{ta\cdot,D_H}$.
    Set $Y_{tam',H} = Y_{tam',H} + 1$.
    Set $Y_{tam',D_H} = Y_{tam',D_H} - 1$.
    Set $Y'_{tas,D_H} = Y'_{tas,D_H} - 1$.
    Set $r = r + 1$.
**end while**
**return** $Y_{tam,H}$, $Y_{tam,D_H}$ and $Y'_{tam,D_H}$ for all $m \in \mathcal{M}_{tas}$.

# G Observation distributions

Let $\mathcal{S}$ denote the set of LTLAs and $\mathcal{A}$ be the set of age-classes used in the model. Also let $\mathcal{R}_r \subset \mathcal{S}$ be the set of LTLAs that make up region $r$ ($r = 1, \dots, N_r$); $\mathcal{N}_n \subset \mathcal{S}$ be the set of LTLAs that make up NHS region $n$ ($n = 1, \dots, N_n$); and $\mathcal{B}_b \subset \mathcal{A}$ be the set of age-classes that make up NHS age-class $b$ ($b = 1, \dots, N_b$). With a slight abuse of notation, we use the specific subscripts ($a$, $b$, $s$ and $r$) to denote the aggregation level of the observation. Hence at each time $t$ we observe the death incidence by age-class and region, $Z'_{tar,D}$ ($a = 1, \dots, N_a$ and $r = 1, \dots, N_r$), and the death incidence in each LTLA, $Z'_{t\cdot s,D}$ ($s = 1, \dots, N_s$). We also observe the hospital incidence by NHS age-class and NHS region, $Z'_{tbn,H}$ ($b = 1, \dots, N_b$ and $n = 1, \dots, N_n$), and the number of people in hospital in each NHS region (but not broken down by age), $Z_{t\cdot n,H}$ (see the *Data* section in the main text for more details). We also know that the data at different aggregations are *not independent*; for example, $Z'_{tar,D}$ and $Z'_{t\cdot s,D}$ are correlated for $s \in \mathcal{R}_r$. Furthermore, there are additional errors in the data sets which means they do not exactly match when aggregated up to a common level (for example, $\sum_{s \in \mathcal{R}_r} Z'_{t\cdot s,D} \neq \sum_{a=1}^{N_a} Z'_{tar,D}$ for all $r = 1, \dots, N_r$, even though they should match).

One way to deal with these issues is to introduce a set of latent variables, denoting the hypothetical observation error (OE) at the LTLA/age-class level of the model. For example, for deaths we could introduce $Z'_{tas,D_H}$ and

$Z'_{tas,D_I}$, from which:

$$Z'_{tar,D} = \sum_{s \in \mathcal{R}_r} \left(Z'_{tas,D_H} + Z'_{tas,D_I}\right), \text{and} \tag{A.15}$$

$$Z'_{t \cdot s,D} = \sum_{a=1}^{N_a} \left(Z'_{tas,D_H} + Z'_{tas,D_I}\right). \tag{A.16}$$

Given functional forms for the p.m.f.s of $Z'_{tar,D}$ and $Z'_{t \cdot s,D}$, one may be able to derive the relevant joint distribution $g\left(\boldsymbol{Z}'_{t(1:N_a)(1:N_r),D}, \boldsymbol{Z}'_{t(1:N_s),D} \mid \boldsymbol{Y}'_t\right)$. In Part 1 Williamson et al. [2025] we discussed the use of Skellam distributions for the observation error, but as for the MD terms, here we chose truncated discrete Gaussian distributions instead, which have similar properties but are more computationally tractable. As such, we could use

$$\begin{aligned} Z'_{tas,c} &= Y'_{tas,c} + \delta'_{tas,c} \\ \delta'_{tas,c} \mid Y'_{tas,c} &\sim tN^D\left(a_1 - a_2 + (b_1 - b_2)\, Y'_{tas,c}, a_1 + a_2 + (b_1 + b_2)\, Y'_{tas,c}, -Y'_{tas,c}, \infty\right) \end{aligned} \tag{A.17}$$

for the OE around the *incidence* for some state $c$ (with analogous forms if the OE is placed around a *count* instead of the incidence). Here $a_1, a_2, b_1, b_2 > 0$ are parameters that enable us to control the mean and variance of the OE process and their dependence on the hidden states (see *Reporting errors* section in Part 1 Williamson et al. [2025]).

We note that the upper bound of the OE should be finite (bounded by the data), but in practice the hidden states represent a small proportion of the overall population and unless the OE variance is very large (A.17) is a suitable approximation. We set $a_1 = a_2 = a_{\text{OE}}$ and $b_1 = b_2 = b_{\text{OE}}$ here, which simplifies to

$$\begin{aligned} Z'_{tas,c} &= Y'_{tas,c} + \delta'_{tas,c} \\ \delta'_{tas,c} \mid Y'_{tas,c} &\sim tN^D\left(0, 2a_{\text{OE}} + 2b_{\text{OE}} Y'_{tas,c}, -Y'_{tas,c}, \infty\right), \end{aligned}$$

or equivalently

$$Z'_{tas,c} \mid Y'_{tas,c} \sim tN^D\left(Y'_{tas,c}, 2a_{\text{OE}} + 2b_{\text{OE}} Y'_{tas,c}, 0, \infty\right).$$

We note that setting $a_1 < a_2$ allows for average under-reporting to be introduced, but with the benefit that the OE will always have a non-zero probability mass function (in contrast to a stricter binomial-type OE model, which will have a zero likelihood if the hidden states are simulated to be larger than the observed states. This helps to alleviate some challenges with particle depletion in the subsequent particle filters). We will explore these variations in future work.

Another challenge is that the sums of truncated discrete Gaussians in equations (A.15)–(A.16) do not have convenient mathematical forms, so instead we work with a set of *continuous*, *unbounded* latent variables,

$$\tilde{Z}'_{tas,c} \mid Y'_{tas,c} \sim N\left(Y'_{tas,c}, 2a_{\text{OE}} + 2b_{\text{OE}} Y'_{tas,c}\right), \tag{A.18}$$

where $\tilde{Z}'_{tas,c}$ now corresponds to the *expected* observation given the hidden states. Since these are Gaussian, then any sum of these is also Gaussian, and for deaths this allows us to specify:

$$\begin{aligned} \tilde{Z}'_{tar,D} &= \sum_{s \in \mathcal{R}_r} \left(\tilde{Z}'_{tas,D_H} + \tilde{Z}'_{tas,D_I}\right), \\ \tilde{Z}'_{t \cdot s,D} &= \sum_{a=1}^{N_a} \left(\tilde{Z}'_{tas,D_H} + \tilde{Z}'_{tas,D_I}\right), \\ Z'_{tar,D} \mid \tilde{Z}'_{tar,D} &\sim tN^D\left(\tilde{Z}'_{tar,D}, \sigma^2_{D_{RA}}, 0, \infty\right), \\ Z'_{t \cdot s,D} \mid \tilde{Z}'_{t \cdot s,D} &\sim tN^D\left(\tilde{Z}'_{t \cdot s,D}, \sigma^2_{D_S}, 0, \infty\right). \end{aligned} \tag{A.19}$$

Here $\sigma^2_{D_{RA}}$ and $\sigma^2_{D_S}$ are additional variance terms chosen to represent additional aggregation errors at the regional/age-class and LTLA levels respectively (described in the *Data* section of the main text).

Since the OE for hospitalisations occurs on both the *incidence* and *counts*, they are not independent of the OE for deaths, since hospital case numbers depend on death and removal incidence as well as hospital incidence. As

such, we also introduce latent variables for the expected observed hospital removals for each age/LTLA. Hence we can specify:

$$
\begin{aligned}
\tilde{Z}'_{tbn,H} &= \sum_{a \in \mathcal{B}_b} \sum_{s \in \mathcal{N}_n} \tilde{Z}'_{tas,H}, \\
\tilde{Z}_{t \cdot n,H} &= \sum_{a=1}^{N_a} \sum_{s \in \mathcal{N}_n} \left( Y_{(t-1)as,H} + \tilde{Z}'_{tas,H} - \tilde{Z}'_{tas,R_H} - \tilde{Z}'_{tas,D_H} \right), \\
Z'_{tbn,H} \mid \tilde{Z}'_{tbn,H} &\sim tN^D \left( \tilde{Z}'_{tbn,H}, \sigma^2_{D_{NB}}, 0, \infty \right), \\
Z_{t \cdot n,H} \mid \tilde{Z}_{t \cdot n,H} &\sim tN^D \left( \tilde{Z}_{t \cdot n,H}, \sigma^2_{D_N}, 0, \infty \right).
\end{aligned}
\tag{A.20}
$$

Here $\sigma^2_{D_{NB}}$ and $\sigma^2_{D_N}$ are additional variance terms chosen to represent the additional aggregation errors at the NHS region/NHS age-class and NHS region levels described above. The correlation structure between the multiple aggregations of death and hospitalisation data is thus captured through this hierarchical latent process. The aggregation error process captures the additional recording errors as described above. In the examples in this paper we chose $a_{\text{OE}} = 0.001$ and $b_{\text{OE}} = 0.025$. We then chose $\sigma^2_{D_{RA}} = 1/\left(N_a + N_r\right)$, $\sigma^2_{D_S} = 1/N_s$, $\sigma^2_{D_{NB}} = 1/\left(N_n + N_b\right)$ and $\sigma^2_{D_N} = 1/N_n$.

# H Likelihood

To facilitate some of the later discussion regarding the particle filters, let's consider the form of the likelihood functions for the simulator, model discrepancy and observation terms as described above. In all discussions below we drop dependence on the parameters, $\boldsymbol{\theta}$, for brevity, and we use $p(\cdot)$ to denote an arbitrary probability *mass* function. To be consistent with much of the particle filtering literature, we will use $g(\cdot)$ to denote a generic observation distribution across all observations, and $f(\cdot)$ to denote a joint state transition distribution.

Since we have fixed initial conditions, the target likelihood can be written as:

$$
\prod_{t=1}^{T} g\left(\boldsymbol{Z}_t, \boldsymbol{Z}'_t \mid \tilde{\boldsymbol{Z}}'_t, \boldsymbol{Y}_{t-1}\right) g\left(\tilde{\boldsymbol{Z}}'_t \mid \boldsymbol{Y}'_t\right) f\left(\boldsymbol{Y}_t \mid \boldsymbol{X}_t, \boldsymbol{Y}_{t-1}\right) f\left(\boldsymbol{X}_t \mid \boldsymbol{Y}_{t-1}\right). \tag{A.21}
$$

(The dependence on $\boldsymbol{Y}_{t-1}$ in $g\left(\boldsymbol{Z}_t, \boldsymbol{Z}'_t \mid \tilde{\boldsymbol{Z}}'_t, \boldsymbol{Y}_{t-1}\right)$ is required for calculating $\tilde{\boldsymbol{Z}}_{t,H}$ in (A.20).) The component relating to the transmission model can be written as:

$$
\begin{aligned}
f\left(\boldsymbol{X}_t \mid \boldsymbol{Y}_{t-1}\right) = {} & p\left(\boldsymbol{X}'_{t,D_H}, \boldsymbol{X}'_{t,R_H}, \cdot \mid \boldsymbol{Y}_{t-1}\right) p\left(\boldsymbol{X}'_{t,H}, \boldsymbol{X}'_{t,I_2}, \boldsymbol{X}'_{t,D_I}, \cdot \mid \boldsymbol{Y}_{t-1}\right) p\left(\boldsymbol{X}'_{t,R_I} \mid \boldsymbol{Y}_{t-1}\right) \\
& \times p\left(\boldsymbol{X}'_{t,I_1} \mid \boldsymbol{Y}_{t-1}\right) p\left(\boldsymbol{X}'_{t,P}, \boldsymbol{X}'_{t,A} \mid \boldsymbol{Y}_{t-1}\right) p\left(\boldsymbol{X}'_{t,R_A} \mid \boldsymbol{Y}_{t-1}\right) \\
& \times p\left(\boldsymbol{X}'_{t,E} \mid \boldsymbol{Y}_{t-1}\right).
\end{aligned}
\tag{A.22}
$$

The infection distribution, $p\left(\boldsymbol{X}'_{t,E} \mid \boldsymbol{Y}_{t-1}\right)$ is a joint distribution over all spatial areas, age-classes and movement cohorts, whereas all other transitions are independent over area and age-class. For example:

$$
p\left(\boldsymbol{X}'_{t,D_H}, \boldsymbol{X}'_{t,R_H}, \cdot \mid \boldsymbol{Y}_{t-1}\right) = \prod_{a=1}^{N_a} \prod_{s=1}^{N_s} p\left(X'_{tas,D_H}, X'_{tas,R_H}, \cdot \mid \boldsymbol{Y}_{(t-1)sa}\right),
$$

and similarly for the other transitions. See Section C for details of each transition distribution.

The conditional model discrepancy distribution can be written as:

$$
\begin{aligned}
f\left(\boldsymbol{Y}_t \mid \boldsymbol{X}_t, \boldsymbol{Y}_{t-1}\right) = {} & p\left(\boldsymbol{Y}'_{t,D_H} \mid \boldsymbol{X}_t, \boldsymbol{Y}_{t-1}\right) p\left(\boldsymbol{Y}'_{t,D_I} \mid \boldsymbol{X}_t, \boldsymbol{Y}_{t-1}\right) p\left(\boldsymbol{Y}'_{t,R_H} \mid \boldsymbol{Y}'_{t,D_H}, \boldsymbol{X}_t, \boldsymbol{Y}_{t-1}\right) \\
& \times p\left(\boldsymbol{Y}'_{t,R_I} \mid \boldsymbol{X}_t, \boldsymbol{Y}_{t-1}\right) p\left(\boldsymbol{Y}'_{t,R_A} \mid \boldsymbol{X}_t, \boldsymbol{Y}_{t-1}\right) \\
& \times p\left(\boldsymbol{Y}_{t,H} \mid \boldsymbol{Y}'_{t,D_H}, \boldsymbol{Y}'_{t,R_H}, \boldsymbol{Y}'_{t,D_I}, \boldsymbol{X}_t, \boldsymbol{Y}_{t-1}\right) \\
& \times p\left(\boldsymbol{Y}_{t,I_2} \mid \boldsymbol{Y}'_{t,H}, \boldsymbol{Y}'_{t,D_I}, \boldsymbol{Y}'_{t,R_I}, \boldsymbol{X}_t, \boldsymbol{Y}_{t-1}\right) \\
& \times p\left(\boldsymbol{Y}_{t,I_1} \mid \boldsymbol{Y}'_{t,I_2}, \boldsymbol{Y}'_{t,D_I}, \boldsymbol{Y}'_{t,H}, \boldsymbol{X}_t, \boldsymbol{Y}_{t-1}\right) \\
& \times p\left(\boldsymbol{Y}_{t,P} \mid \boldsymbol{Y}'_{t,I_1}, \boldsymbol{X}_t, \boldsymbol{Y}_{t-1}\right) p\left(\boldsymbol{Y}_{t,A} \mid \boldsymbol{Y}'_{t,R_A}, \boldsymbol{X}_t, \boldsymbol{Y}_{t-1}\right) \\
& \times p\left(\boldsymbol{Y}_{t,E} \mid \boldsymbol{Y}'_{t,P}, \boldsymbol{Y}'_{t,A}, \boldsymbol{X}_t, \boldsymbol{Y}_{t-1}\right),
\end{aligned}
\tag{A.23}
$$

and here all the component distributions in (A.23) are independent over spatial area and age given $\boldsymbol{X}_t$ and $\boldsymbol{Y}_{t-1}$. We note that some of the terms depend on **incidence** counts from the transmission model (e.g. $X'_{tas,D_H}$), but these can be directly derived from $\boldsymbol{X}_t$ and $\boldsymbol{Y}_{t-1}$ as required. To keep notation as simple as possible, we have only made explicit the conditional dependence of the various MD states on other MD states in (A.23).

The first component of the conditional observation density can be written as:

$$g\left(\tilde{\boldsymbol{Z}}'_t \mid \boldsymbol{Y}'_t\right) = \prod_{a=1}^{N_a}\prod_{s=1}^{N_s}\prod_{c\in(D_H,R_H,H,D_I)} p\left(\tilde{Z}'_{tas,c} \mid Y'_{tas,c}\right), \tag{A.24}$$

where the $p\left(\tilde{Z}'_{tas,c} \mid Y'_{tas,c}\right)$ are given by (A.18). Then we have

$$\begin{aligned} g\left(\boldsymbol{Z}_t, \boldsymbol{Z}'_t \mid \tilde{\boldsymbol{Z}}'_t, \boldsymbol{Y}_{t-1}\right) = &\left[\prod_{r=1}^{N_r}\prod_{a=1}^{N_a} p\left(Z'_{tar,D} \mid \tilde{Z}'_{tar,D}\right)\right]\left[\prod_{s=1}^{N_s} p\left(Z'_{t\cdot s,D} \mid \tilde{Z}'_{t\cdot s,D}\right)\right] \\ &\times \left[\prod_{n=1}^{N_n}\prod_{b=1}^{N_b} p\left(Z'_{tbn,H} \mid \tilde{Z}'_{tbn,H}\right)\right]\left[\prod_{n=1}^{N_n} p\left(Z_{t\cdot n,H} \mid \tilde{Z}_{t\cdot n,H}\right)\right]. \end{aligned} \tag{A.25}$$

As an aside, we note that for other data structures it may be possible to analytically integrate across the continuous latent states, in which case the observation density could be written as:

$$\begin{aligned} g\left(\boldsymbol{Z}_t, \boldsymbol{Z}'_t \mid \boldsymbol{Y}_{(t-1):t}\right) = &\int_{\mathcal{Z}'_{t,D_H}}\int_{\mathcal{Z}'_{t,R_H}}\int_{\mathcal{Z}'_{t,H}}\int_{\mathcal{Z}'_{t,D_I}} g\left(\boldsymbol{Z}_t, \boldsymbol{Z}'_t \mid \tilde{\boldsymbol{Z}}'_t, \boldsymbol{Y}_{t-1}\right) \\ &\times g\left(\tilde{\boldsymbol{Z}}'_t \mid \boldsymbol{Y}'_t\right) d\tilde{\boldsymbol{Z}}'_{t,D_I} d\tilde{\boldsymbol{Z}}'_{t,H} d\tilde{\boldsymbol{Z}}'_{t,R_H} d\tilde{\boldsymbol{Z}}'_{t,D_H}, \end{aligned} \tag{A.26}$$

where the $\mathcal{Z}'_{t,c}$ represent the multi-dimensional latent spaces for the $\tilde{\boldsymbol{Z}}'_{t,c}$ terms for state $c \in (D_H, R_H, H, D_I)$. If possible, this would reduce the uncertainty in the likelihood estimates from the particle filter caused by having to simulate the additional latent states.

# I Bootstrap particle filter

A standard bootstrap particle filter (BPF) [Gordon et al., 1993] for this model is shown in Algorithm B, along with some amendments to help alleviate particle impoverishment using Markov chain Monte Carlo (MCMC; Gilks et al., 1996) steps [Gilks and Berzuini, 2001, Doucet and Johansen, 2011].

## I.1 Tackling particle impoverishment

In a complex spatial meta-population model such as that described here, it is common (with a small, finite number of particles), to experience partial, or complete, particle degeneracy and impoverishment at any given time $t$, thus resulting in the particles having low diversity and in extreme cases all particle trajectories can collapse onto a single trajectory. Following e.g. Gilks and Berzuini [2001] and Doucet and Johansen [2011] we can introduce variability by taking each particle in turn and performing a series of $N_{\text{iter}}$ MCMC steps, using a Markov kernel, $K_t(\cdot)$, of invariant distribution $p\left(\boldsymbol{X}_{1:t}, \boldsymbol{Y}_{1:t} \mid \boldsymbol{Z}_{1:t}\right)$. Such a kernel is only ergodic if all $\boldsymbol{X}_{1:t}$ and $\boldsymbol{Y}_{1:t}$ are updated, but this would be too computationally intensive, and so following Doucet and Johansen [2011] we will update only $\left(\boldsymbol{X}_t, \boldsymbol{Y}_t \mid \boldsymbol{X}_{1:(t-1)}, \boldsymbol{Y}_{1:(t-1)}, \boldsymbol{Z}_{1:t}\right)$ at each iteration $t$. We exploit the structure of the model and MD terms to produce efficient updates using *independence sampler* Metropolis-Hastings steps. The reader is referred to e.g. Gilks et al. [1996] for a more detailed introduction to MCMC.

To simplify future discussions, note that (from Section C) we can decompose

$$p\left(X'_{tas,D_H}, X'_{tas,R_H}, \cdot \mid \boldsymbol{Y}_{(t-1)as}\right) = p\left(X'_{tas,D_H} \mid \boldsymbol{Y}_{(t-1)as}\right) p\left(X'_{tas,R_H} \mid X'_{tas,D_H}, \boldsymbol{Y}_{(t-1)as}\right), \tag{A.27}$$

and

$$\begin{aligned} p\left(X'_{tas,H}, X'_{tas,I_2}, X'_{tas,D_I}, \cdot \mid \boldsymbol{Y}_{(t-1)as}\right) = &\, p\left(X'_{tas,D_I} \mid \boldsymbol{Y}_{(t-1)as}\right) \\ &\times p\left(X'_{tas,H}, X'_{tas,I_2}, \cdot \mid X'_{tas,D_I}, \boldsymbol{Y}_{(t-1)as}\right), \end{aligned} \tag{A.28}$$

**Algorithm B** Bootstrap Particle Filter with MCMC steps

**Require:** Data $\boldsymbol{Z}_{1:t}$ and a number of particles $K$.
**for** $k = 1, \ldots, K$ **do**
    Set initial states at time $t = 0$: $\boldsymbol{Y}_0^k$.
    Set normalised weight $\tilde{w}_0^k = \dfrac{1}{K}$.
**end for**
**for** $t = 1, \ldots, T$ **do**
    **for** $k = 1, \ldots, K$ **do**
        Sample $\boldsymbol{X}_t^k \sim f\left(\boldsymbol{X}_t \mid \boldsymbol{Y}_{t-1}^k\right)$.
        Sample $\boldsymbol{Y}_t^k \sim f\left(\boldsymbol{Y}_t \mid \boldsymbol{X}_t^k, \boldsymbol{Y}_{t-1}^k\right)$.
        Sample $\tilde{\boldsymbol{Z}}_t'^k \sim g\left(\tilde{\boldsymbol{Z}}_t' \mid \boldsymbol{Y}_t'^k\right)$.
        Set un-normalised weights $w_t^k = g\left(\boldsymbol{Z}_t, \boldsymbol{Z}_t' \mid \tilde{\boldsymbol{Z}}_t'^k, \boldsymbol{Y}_{t-1}^k\right)$.
    **end for**
    Estimate $\hat{p}^A\left(\boldsymbol{Z}_t \mid \boldsymbol{Z}_{t-1}\right) = \dfrac{1}{K} \sum_{k=1}^{K} w_t^k$.
    **for** $k = 1, \ldots, K$ **do**
        Calculate normalised weights $\tilde{w}_t^k = \dfrac{w_t^k}{\sum_{j=1}^K w_t^j}$.
    **end for**
    **for** $k = 1, \ldots, K$ **do**
        Sample a particle index $r \in \{1, \ldots, K\}$, with probabilities $\tilde{w}_t^{1:K}$.
        Set $\boldsymbol{X}_t^{*k} = \boldsymbol{X}_t^r$, $\boldsymbol{Y}_t^{*k} = \boldsymbol{Y}_t^r$ and $\tilde{\boldsymbol{Z}}_t'^{*k} = \tilde{\boldsymbol{Z}}_t'^r$.
    **end for**
    Update $\boldsymbol{X}_t = \boldsymbol{X}_t^*$, $\boldsymbol{Y}_t = \boldsymbol{Y}_t^*$ and $\tilde{\boldsymbol{Z}}_t' = \tilde{\boldsymbol{Z}}_t'^*$.
    **for** $k = 1, \ldots, K$ **do**
        Set $N_{\text{acc}} = 0$.
        **for** $i = 1, \ldots, N_{\text{iter}}$ **do**
            Sample $\boldsymbol{X}_t^{z*} \sim f\left(\boldsymbol{X}_t^z \mid \boldsymbol{Y}_{t-1}^k\right)$.
            Sample $\boldsymbol{Y}_t^{z*} \sim f\left(\boldsymbol{Y}_t^z \mid \boldsymbol{X}_t^{z*}, \boldsymbol{Y}_{t-1}^k\right)$.
            Sample $\tilde{\boldsymbol{Z}}_t'^{z*} \sim g\left(\tilde{\boldsymbol{Z}}_t'^z \mid \boldsymbol{Y}_t'^{z*}\right)$.
            Set

$$\alpha = \min\left(1, \frac{g\left(\boldsymbol{Z}_t, \boldsymbol{Z}_t' \mid \tilde{\boldsymbol{Z}}_t'^{z*}, \boldsymbol{Y}_{t-1}^k\right)}{g\left(\boldsymbol{Z}_t, \boldsymbol{Z}_t' \mid \tilde{\boldsymbol{Z}}_t'^{zk}, \boldsymbol{Y}_{t-1}^k\right)}\right).$$

            Sample $u \sim U(0,1)$.
            **if** $u < \alpha$ **then**
                Set $\boldsymbol{X}_t^{zk} = \boldsymbol{X}_t^{z*}$, $\boldsymbol{Y}_t^{zk} = \boldsymbol{Y}_t^{z*}$ and $\tilde{\boldsymbol{Z}}_t'^{zk} = \tilde{\boldsymbol{Z}}_t'^{z*}$.
                Set $N_{\text{acc}} = N_{\text{acc}} + 1$.
            **end if**
        **end for**
        **if** $N_{\text{acc}} > 0$ **then**
            Sample $\boldsymbol{X}_t^{-zk} \sim f\left(\boldsymbol{X}_t^{-z} \mid \boldsymbol{X}_t^{zk}, \boldsymbol{Y}_{t-1}^k\right)$.
            Sample $\boldsymbol{Y}_t^{-zk} \sim f\left(\boldsymbol{Y}_t^{-z} \mid \boldsymbol{Y}_t^{zk}, \boldsymbol{X}_t^k, \boldsymbol{Y}_{t-1}^k\right)$.
        **end if**
    **end for**
**end for**
**return** $\hat{p}^A\left(\boldsymbol{Z}_{1:T}\right) = \prod_{t=1}^T \hat{p}^A\left(\boldsymbol{Z}_t \mid \boldsymbol{Z}_{t-1}\right)$.

and since the distributions on the left-hand sides of (A.27) and (A.28) are multinomial, the marginal distributions on the right-hand sides are binomial and the conditional distributions are either binomial or multinomial.

Letting $\boldsymbol{Y}_t^z = \{Y_{tas,D_H}, Y_{tas,R_H}, Y_{tas,H}, Y_{tas,D_I}; a = 1, \ldots, N_a \text{ and } s = 1, \ldots, N_s\}$ be the adjusted states at the age-class/LTLA level that are necessary for mapping to the observed states $\boldsymbol{Z}_t$, with $\boldsymbol{X}_t^z$ denoting the corresponding states generated from the simulator, then based on (A.21)–(A.28) we can write the joint likelihood for the hidden states and the data at time $t$ as:

$$g\left(\boldsymbol{Z}_t, \boldsymbol{Z}'_t \mid \tilde{\boldsymbol{Z}}'_t, \boldsymbol{Y}_{t-1}\right) g\left(\tilde{\boldsymbol{Z}}'_t \mid \boldsymbol{Y}'_t\right) f\left(\boldsymbol{Y}_t^{-z} \mid \boldsymbol{Y}_t^z, \boldsymbol{X}_t, \boldsymbol{Y}_{t-1}\right) f\left(\boldsymbol{Y}_t^z \mid \boldsymbol{X}_t, \boldsymbol{Y}_{t-1}\right) \times f\left(\boldsymbol{X}_t^{-z} \mid \boldsymbol{X}_t^z, \boldsymbol{Y}_{t-1}\right) f\left(\boldsymbol{X}_t^z \mid \boldsymbol{Y}_{t-1}\right), \tag{A.29}$$

where we denote $\boldsymbol{Y}_t^{-z}$ as those states in $\boldsymbol{Y}_t$ that *do not* include those in $\boldsymbol{Y}_t^z$, and similarly for $\boldsymbol{X}_t^{-z}$.

We can further simplify the dependencies, and then exploit these to produce efficient update schemes. Firstly, from Section E we can see that

$$f\left(\boldsymbol{Y}_t^z \mid \boldsymbol{X}_t, \boldsymbol{Y}_{t-1}\right) = f\left(\boldsymbol{Y}_t^z \mid \boldsymbol{X}_t^z, \boldsymbol{Y}_{t-1}\right), \tag{A.30}$$

and from (A.24)–(A.25) we can also see that

$$g\left(\boldsymbol{Z}_t, \boldsymbol{Z}'_t \mid \tilde{\boldsymbol{Z}}'_t, \boldsymbol{Y}_{t-1}\right) g\left(\tilde{\boldsymbol{Z}}'_t \mid \boldsymbol{Y}'_t\right) = g\left(\boldsymbol{Z}_t, \boldsymbol{Z}'_t \mid \tilde{\boldsymbol{Z}}_t'^z, \boldsymbol{Y}_{t-1}\right) g\left(\tilde{\boldsymbol{Z}}_t'^z \mid \boldsymbol{Y}_t'^z\right). \tag{A.31}$$

From the decomposition in (A.29) we can then write the likelihood contribution at time $t$ as:

$$\underbrace{g\left(\boldsymbol{Z}_t, \boldsymbol{Z}'_t \mid \tilde{\boldsymbol{Z}}_t'^z, \boldsymbol{Y}_{t-1}\right) g\left(\tilde{\boldsymbol{Z}}_t'^z \mid \boldsymbol{Y}_t'^z\right)}_{a} \underbrace{f\left(\boldsymbol{Y}_t^{-z} \mid \boldsymbol{Y}_t^z, \boldsymbol{X}_t, \boldsymbol{Y}_{t-1}\right) f\left(\boldsymbol{X}_t^{-z} \mid \boldsymbol{X}_t^z, \boldsymbol{Y}_{t-1}\right)}_{b} \times \underbrace{f\left(\boldsymbol{Y}_t^z \mid \boldsymbol{X}_t^z, \boldsymbol{Y}_{t-1}\right) f\left(\boldsymbol{X}_t^z \mid \boldsymbol{Y}_{t-1}\right)}_{c}. \tag{A.32}$$

From (A.22) we have that:

$$f\left(\boldsymbol{X}_t^z \mid \boldsymbol{Y}_{t-1}\right) = \prod_{a=1}^{N_a} \prod_{s=1}^{N_s} p\left(X'_{tas,D_H} \mid \boldsymbol{Y}_{(t-1)as}\right) p\left(X'_{tas,R_H} \mid X'_{tas,D_H}, \boldsymbol{Y}_{(t-1)as}\right) \times p\left(X'_{tas,D_I} \mid \boldsymbol{Y}_{(t-1)as}\right) p\left(X'_{tas,H} \mid X'_{tas,D_I}, \boldsymbol{Y}_{(t-1)as}\right), \tag{A.33}$$

and from (A.23) and Section E section we have that:

$$f\left(\boldsymbol{Y}_t^z \mid \boldsymbol{X}_t^z, \boldsymbol{Y}_{t-1}\right) = \prod_{a=1}^{N_a} \prod_{s=1}^{N_s} p\left(Y'_{tas,D_H} \mid X'_{tas,D_H}, \boldsymbol{Y}_{t-1}\right) p\left(Y'_{tas,R_H} \mid Y'_{tas,D_H}, X'_{tas,R_H}, \boldsymbol{Y}_{t-1}\right) \times p\left(Y'_{tas,D_I} \mid X'_{tas,D_I}, \boldsymbol{Y}_{t-1}\right) \times p\left(Y'_{tas,H} \mid Y'_{tas,D_I}, Y'_{tas,D_H}, Y'_{tas,R_H}, X'_{tas,H}, \boldsymbol{Y}_{t-1}\right). \tag{A.34}$$

For a given particle $k$, we can simulate proposals $\boldsymbol{X}_t^{z*}$ and $\boldsymbol{Y}_t^{z*}$ from (A.32c) easily by sampling directly from (A.33) and then (A.34). We can then simulate proposals $\boldsymbol{X}_t^{-z*}$ and $\boldsymbol{Y}_t^{-z*}$ given $\boldsymbol{X}_t^{z*}$ and $\boldsymbol{Y}_t^{z*}$ from (A.32b), by sampling directly from the conditional distributions $f\left(\boldsymbol{X}_t^{-z*} \mid \boldsymbol{X}_t^{z*}, \boldsymbol{Y}_{t-1}^k\right)$ and $f\left(\boldsymbol{Y}_t^{-z*} \mid \boldsymbol{Y}_t^{z*}, \boldsymbol{X}_t^*, \boldsymbol{Y}_{t-1}^k\right)$ respectively. Details for how to sample from these conditional distributions are given in Section J, and one particularly desirable feature is that $f\left(\boldsymbol{X}_t^{-z*} \mid \boldsymbol{X}_t^{z*}, \boldsymbol{Y}_{t-1}^k\right)$ can be sampled from by simply changing some of the inputs in the simulator model, meaning that it can be evaluated with minimal changes to the code-base. Finally, we can sample proposals $\tilde{\boldsymbol{Z}}_t'^*$ directly from $g\left(\tilde{\boldsymbol{Z}}'_t \mid \boldsymbol{Y}_t'^*\right)$ defined in (A.24).

With this proposal mechanism and the likelihood structure in (A.32), we can see that these proposals are accepted with probability:

$$\alpha = \min\left(1, \frac{g\left(\boldsymbol{Z}_t, \boldsymbol{Z}'_t \mid \tilde{\boldsymbol{Z}}_t'^{z*}, \boldsymbol{Y}_{t-1}^k\right)}{g\left(\boldsymbol{Z}_t, \boldsymbol{Z}'_t \mid \tilde{\boldsymbol{Z}}_t'^{z(i)}, \boldsymbol{Y}_{t-1}^k\right)}\right), \tag{A.35}$$

where $\tilde{\boldsymbol{Z}}_t'^{z(i)}$ is the value of $\tilde{\boldsymbol{Z}}_t'^z$ at iteration $i$ ($i = 1, \ldots, N_{\text{iter}}$).

The computational burden of this proposed update scheme is sampling from (A.32b). However, it is worth noting that we do not actually have to sample from (A.32b) at all in order to evaluate the accept-reject ratio

(A.35). Furthermore, using the proposal mechanism above means that $\boldsymbol{X}_t^z$ and $\boldsymbol{Y}_t^z$ can be sampled *independently* of $\boldsymbol{X}_t^{-z}$ and $\boldsymbol{Y}_t^{-z}$ given $\boldsymbol{Y}_{t-1}^k$; meaning that multiple iterations of these update steps can be done quickly and efficiently without having to sample from (A.32b). If, at the end of these iterations at least one proposal has been accepted, then we can do a single draw of $\boldsymbol{X}_t^{-z}$ and $\boldsymbol{Y}_t^{-z}$ respectively, given the current values of $\boldsymbol{X}_t^z$ and $\boldsymbol{Y}_t^z$. This is much more efficient than sampling from (A.32b) multiple times. Since proposals can be rejected, running multiple update steps increases the probability of accepting a proposal, and thus increases the likelihood of reducing particle impoverishment.

# J Sampling from conditional distributions

## J.1 Sampling from conditional simulator

It turns out that we can sample from $\pi\left(\boldsymbol{X}_t^{-z} \mid \boldsymbol{X}_t^z, \boldsymbol{Y}_{t-1}\right)$ *directly from the simulator*, by adjusting some parameters and inputs slightly. In the subsequent discussion we present the case for sampling from a single region and age-class, and for clarity we drop the $s$ and $a$ subscripts.

If we first consider

$$\left(X'_{t,D_H}, X'_{t,R_H}, \cdot \mid \boldsymbol{Y}_{t-1}\right) \sim \text{Multinomial}\left(Y_{t-1,H}, \boldsymbol{p}\right), \tag{A.36}$$

where $\boldsymbol{p} = \left(p_H p_{HD}, p_H\left(1-p_{HD}\right), 1-p_H\right)$. (Here the $\cdot$ represents those individuals who do not transition between states.) From this we have that

$$\left(X'_{t,R_H} \mid X'_{t,D_H}, \boldsymbol{Y}_{t-1}\right) \sim \text{Binomial}\left(Y_{t-1,H} - X'_{t,D_H}, \frac{p_H\left(1-p_{HD}\right)}{1-p_H p_{HD}}\right). \tag{A.37}$$

Sampling from (A.37) is equivalent to sampling from

$$\left(D, X'_{t,R_H}, \cdot \mid X'_{t,D_H}, \boldsymbol{Y}_{t-1}\right) \sim \text{Multinomial}\left(Y_{t-1,H} - X'_{t,D_H}, \boldsymbol{p}^*\right), \tag{A.38}$$

where $\boldsymbol{p}^* = \left(0, \frac{p_H(1-p_{HD})}{1-p_H p_{HD}}, \frac{1-p_H}{1-p_H p_{HD}}\right)$ and $D = 0$ is a dummy variable. Similarly, if we consider

$$\left(X'_{t,H}, X'_{t,I_2}, X'_{t,D_I}, \cdot \mid \boldsymbol{Y}_{t-1}\right) \sim \text{Multinomial}\left(Y_{t-1,I_1}, \boldsymbol{q}\right), \tag{A.39}$$

where $\boldsymbol{q} = \left(p_{I_1} p_{I_1 H}, p_{I_1}\left(1 - p_{I_1 H} - p_{I_1 D}\right), p_{I_1} p_{I_1 D}, 1 - p_{I_1}\right)$, then we can derive the conditional:

$$\left(X'_{t,H}, X'_{t,I_2}, \cdot \mid X'_{t,D_I}, \boldsymbol{Y}_{t-1}\right) \sim \text{Multinomial}\left(Y_{t-1,I_1} - X'_{t,D_I}, \boldsymbol{q}'\right), \tag{A.40}$$

where $\boldsymbol{q}' = \left(\frac{p_{I_1} p_{I_1 H}}{1-p_{I_1} p_{I_1 D}}, \frac{p_{I_1}\left(1-p_{I_1 H}-p_{I_1 D}\right)}{1-p_{I_1} p_{I_1 D}}, \frac{1-p_{I_1}}{1-p_{I_1} p_{I_1 D}}\right)$. Sampling from (A.40) is equivalent to sampling from

$$\left(X'_{t,H}, X'_{t,I_2}, D, \cdot \mid X'_{t,D_I}, \boldsymbol{Y}_{t-1}\right) \sim \text{Multinomial}\left(Y_{t-1,I_1} - X'_{t,D_I}, \boldsymbol{q}^*\right), \tag{A.41}$$

where $\boldsymbol{q}^* = \left(\frac{p_{I_1} p_{I_1 H}}{1-p_{I_1} p_{I_1 D}}, \frac{p_{I_1}\left(1-p_{I_1 H}-p_{I_1 D}\right)}{1-p_{I_1} p_{I_1 D}}, 0, \frac{1-p_{I_1}}{1-p_{I_1} p_{I_1 D}}\right)$ and $D = 0$ is a dummy variable. Hence we can use the existing simulator code to sample from the conditional simulator by just adjusting the inputs and parameters.

## J.2 Sampling from conditional model discrepancy distribution

The conditional model discrepancy distribution is:

$$\begin{aligned}
f\left(\boldsymbol{Y}_t^{-z} \mid \boldsymbol{Y}_t^z, \boldsymbol{X}_t^{-z}, \boldsymbol{Y}_{t-1}\right) = {} & p\left(\boldsymbol{Y}'_{t,R_H} \mid \boldsymbol{Y}'_{t,D_H}, \boldsymbol{X}_t, \boldsymbol{Y}_{t-1}\right) p\left(\boldsymbol{Y}'_{t,R_I} \mid \boldsymbol{X}_t, \boldsymbol{Y}_{t-1}\right) p\left(\boldsymbol{Y}'_{t,R_A} \mid \boldsymbol{X}_t, \boldsymbol{Y}_{t-1}\right) \\
& \times p\left(\boldsymbol{Y}_{t,H} \mid \boldsymbol{Y}'_{t,D_H}, \boldsymbol{Y}'_{t,R_H}, \boldsymbol{Y}'_{t,D_I}, \boldsymbol{X}_t, \boldsymbol{Y}_{t-1}\right) \\
& \times p\left(\boldsymbol{Y}_{t,I_2} \mid \boldsymbol{Y}'_{t,H}, \boldsymbol{Y}'_{t,D_I}, \boldsymbol{Y}'_{t,R_I}, \boldsymbol{X}_t, \boldsymbol{Y}_{t-1}\right) \\
& \times p\left(\boldsymbol{Y}_{t,I_1} \mid \boldsymbol{Y}'_{t,I_2}, \boldsymbol{Y}'_{t,D_I}, \boldsymbol{Y}'_{t,H}, \boldsymbol{X}_t, \boldsymbol{Y}_{t-1}\right) p\left(\boldsymbol{Y}_{t,P} \mid \boldsymbol{Y}'_{t,I_1}, \boldsymbol{X}_t, \boldsymbol{Y}_{t-1}\right) \\
& \times p\left(\boldsymbol{Y}_{t,A} \mid \boldsymbol{Y}'_{t,R_A}, \boldsymbol{X}_t, \boldsymbol{Y}_{t-1}\right) p\left(\boldsymbol{Y}_{t,E} \mid \boldsymbol{Y}'_{t,P}, \boldsymbol{Y}'_{t,A}, \boldsymbol{X}_t, \boldsymbol{Y}_{t-1}\right),
\end{aligned} \tag{A.42}$$

and here all the component distributions in (A.42) are independent over space and age given $\boldsymbol{Y}_t^z$, $\boldsymbol{X}_t^{-z}$ and $\boldsymbol{Y}_{t-1}$ and thus are straightforward to sample from.

# K Initial plausible regions

Derivations of the initial plausible regions for the parameters of our initial COVID-19 model are given in Part 1 Williamson et al. [2025]. In addition, for this spatial version we set prior ranges for the introduction of MD in each spatial region between the start of the study and the time of first lockdown ($t_{\text{lockdown}}$). The rate at which the initial MD increases, $\alpha_{\text{MD}}$, is bounded in $(0.1, 1)$. The parameters $\eta_I$ and $\eta_H$ that scale the age-relationship with the death outcome in the symptomatic and hospitalisation pathways respectively, are allowed to vary between $(0.5, 2)$. For a full description of these parameters, see Section C. We also introduced a parameter $p_{\text{move}}$, that is bounded in $(0, 1)$ and represents the effect of lockdown on the probability of movements occurring, and a parameter $\beta_s$, also bounded in $(0, 1)$ that scales the force-of-infection to account for other measures such as social distancing.

| Parameter | Ranges |
|---|---|
| $R_0$ | (2, 4.5) |
| $\nu_A$ | (0, 1) |
| $T_E$ | (0.1, 2) |
| $T_P$ | (1.2, 3) |
| $T_{I_1}$ | (2.8, 4.5) |
| $T_{I_2}$ | (0.0001, 0.5) |
| $\alpha_{EP}$, $\alpha_{I_1H}$, $\alpha_{I_1D}$, $\alpha_{HD}$, $\eta$ | drawn from distribution as described in Part 1 Williamson et al. [2025] |
| $\eta_I$ | (0.5, 2) |
| $\eta_H$ | (0.5, 2) |
| $\alpha_{T_H}$, $\eta_{T_H}$ | drawn from distribution as described in Part 1 Williamson et al. [2025] |
| $\beta_s$ | (0, 1) |
| $p_{\text{move}}$ | (0, 1) |
| $\alpha_{\text{MD}}$ | (0.1, 1) |
| $t_{\text{MD, East of England}}$ | $(0, t_{\text{lockdown}})$ |
| $t_{\text{MD, East Midlands}}$ | $(0, t_{\text{lockdown}})$ |
| $t_{\text{MD, London}}$ | $(0, t_{\text{lockdown}})$ |
| $t_{\text{MD, North-East}}$ | $(0, t_{\text{lockdown}})$ |
| $t_{\text{MD, North-West}}$ | $(0, t_{\text{lockdown}})$ |
| $t_{\text{MD, South-East}}$ | $(0, t_{\text{lockdown}})$ |
| $t_{\text{MD, South-West}}$ | $(0, t_{\text{lockdown}})$ |
| $t_{\text{MD, West Midlands}}$ | $(0, t_{\text{lockdown}})$ |
| $t_{\text{MD, Yorkshire and the Humber}}$ | $(0, t_{\text{lockdown}})$ |

Table A: Initial plausible regions

## L Simulation study parameters

| Parameter | Value |
|---|---|
| $R_0$ | 4 |
| $\nu_A$ | 0.37 |
| $T_E$ | 0.91 |
| $T_P$ | 1.6 |
| $T_{I_1}$ | 3.8 |
| $T_{I_2}$ | 0.24 |
| $\alpha_{EP}$ | -4.3 |
| $\alpha_{I_1H}$ | -3.6 |
| $\alpha_{I_1D}$ | -8.1 |
| $\alpha_{HD}$ | -4.4 |
| $\eta$ | 0.038 |
| $\eta_I$ | 0.74 |
| $\eta_H$ | 1.4 |
| $\alpha_{TH}$ | 0.47 |
| $\eta_{TH}$ | 0.027 |
| $\beta_s$ | 0.27 |
| $p_{\text{move}}$ | 0.49 |
| $\alpha_{MD}$ | 0.24 |
| $t_{\text{MD, East of England}}$ | 20 |
| $t_{\text{MD, East Midlands}}$ | 36 |
| $t_{\text{MD, London}}$ | 26 |
| $t_{\text{MD, North-East}}$ | 25 |
| $t_{\text{MD, North-West}}$ | 18 |
| $t_{\text{MD, South-East}}$ | 34 |
| $t_{\text{MD, South-West}}$ | 29 |
| $t_{\text{MD, West Midlands}}$ | 30 |
| $t_{\text{MD, Yorkshire and the Humber}}$ | 5 |

Table B: Parameters chosen for simulation study (to 2 significant figures)

# M Additional figures

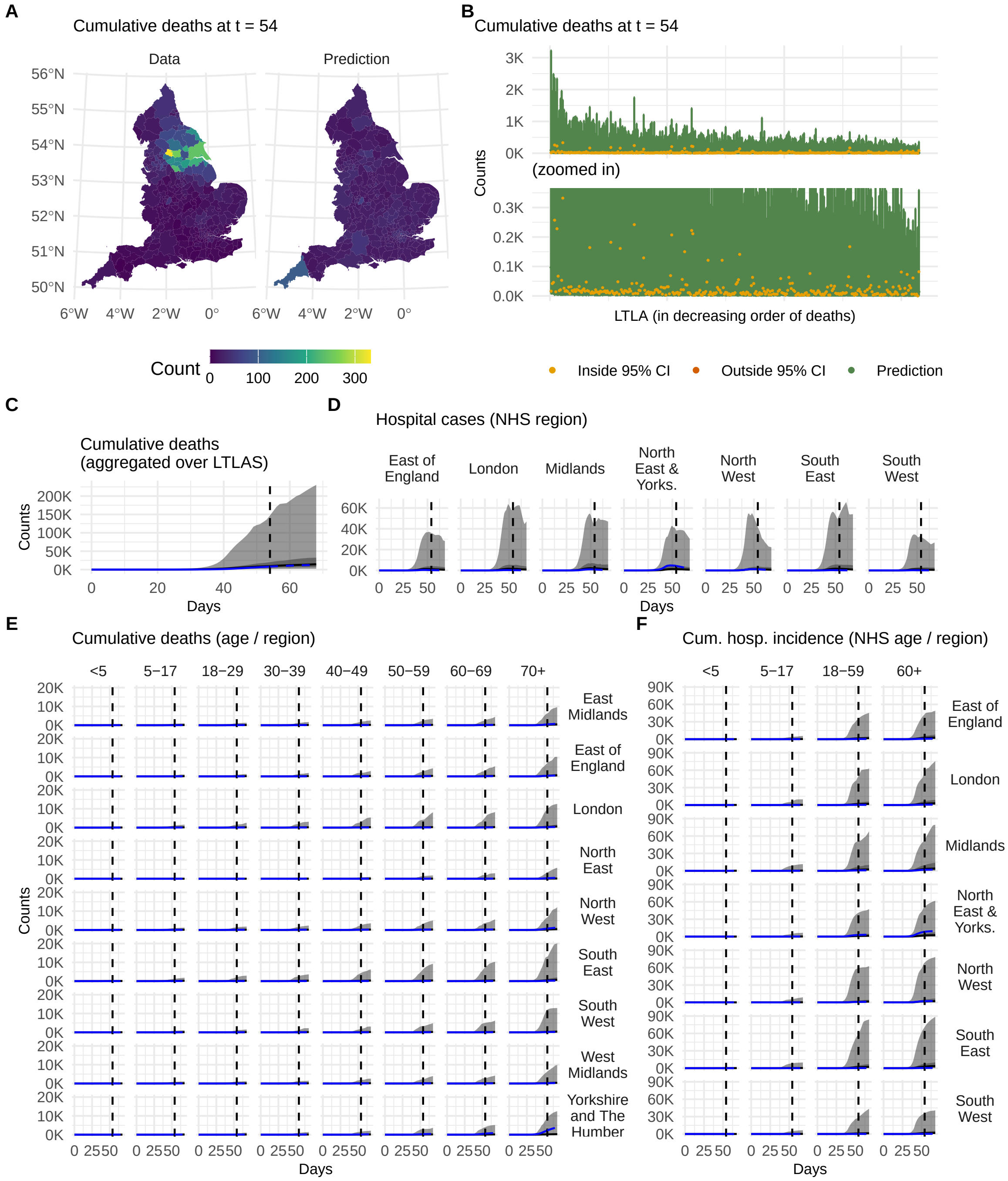


Figure B: Particle trajectory plots across the ensemble of design points at Wave 1 for the simulated outbreak. A) Spatial (LTLA-level) plots of the mean number of deaths by day 54, for the data and the fitted model. B) Cumulative deaths at day 54 within each LTLA (ranked in decreasing order of predicted deaths). C) Cumulative deaths over time, aggregated over the 315 LTLAs with death data available. D) Hospital cases over time in each NHS region. E) Cumulative deaths over time in each age/region category. F) Cumulative hospital incidence over time by each NHS age/region category. In plot B) the green points are the predicted ensemble means, and the error bars are the 95% prediction intervals. For clarity we show a zoomed-in version of the plot also. The yellow and red points are the observed data coloured by whether they lie inside and outside of the prediction intervals respectively. In plots C–F, the blue dashed lines correspond to the observed data and the black solid lines to the mean trajectories from the particles taken across the ensemble. The ribbons correspond to 50% and 95% prediction intervals. A vertical dashed line corresponds to the end point of the observed data, such that trajectories before the line are generated from the particle filter, and trajectories to the right of the line are simulated forecasts from the model. Source for shapefiles: https://geoportal.statistics.gov.uk from the Office for National Statistics licensed under the Open Government Licence v.3.0. Contains OS data: Crown copyright and database right 2022.

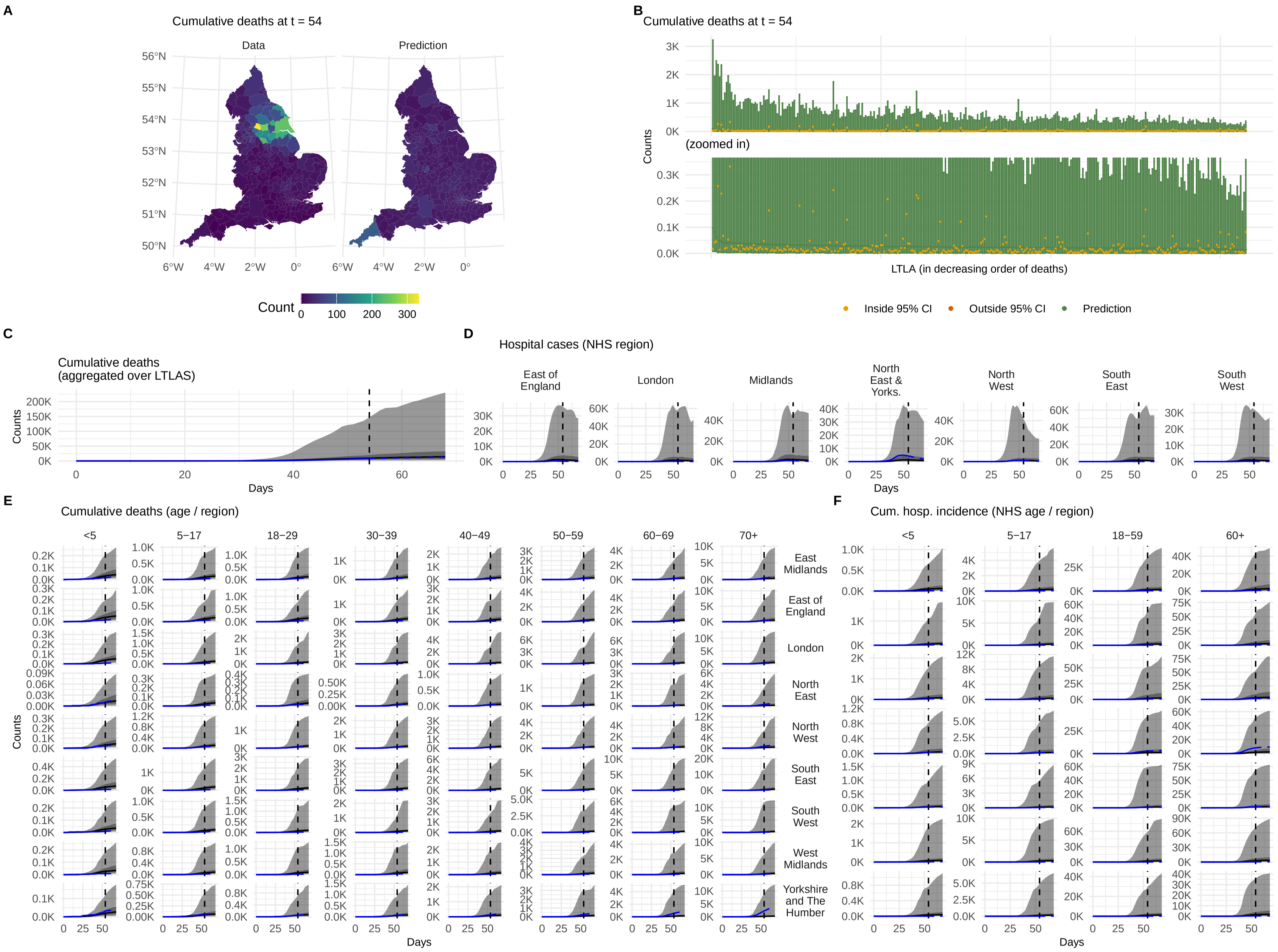


Figure C: Particle trajectory plots across the ensemble of design points at Wave 1 for the simulated outbreak. Plots identical to Figure B except $y$-axes are allowed to vary in D–F to better examine model fits. Source for shapefiles: https://geoportal.statistics.gov.uk from the Office for National Statistics licensed under the Open Government Licence v.3.0. Contains OS data: Crown copyright and database right 2022.

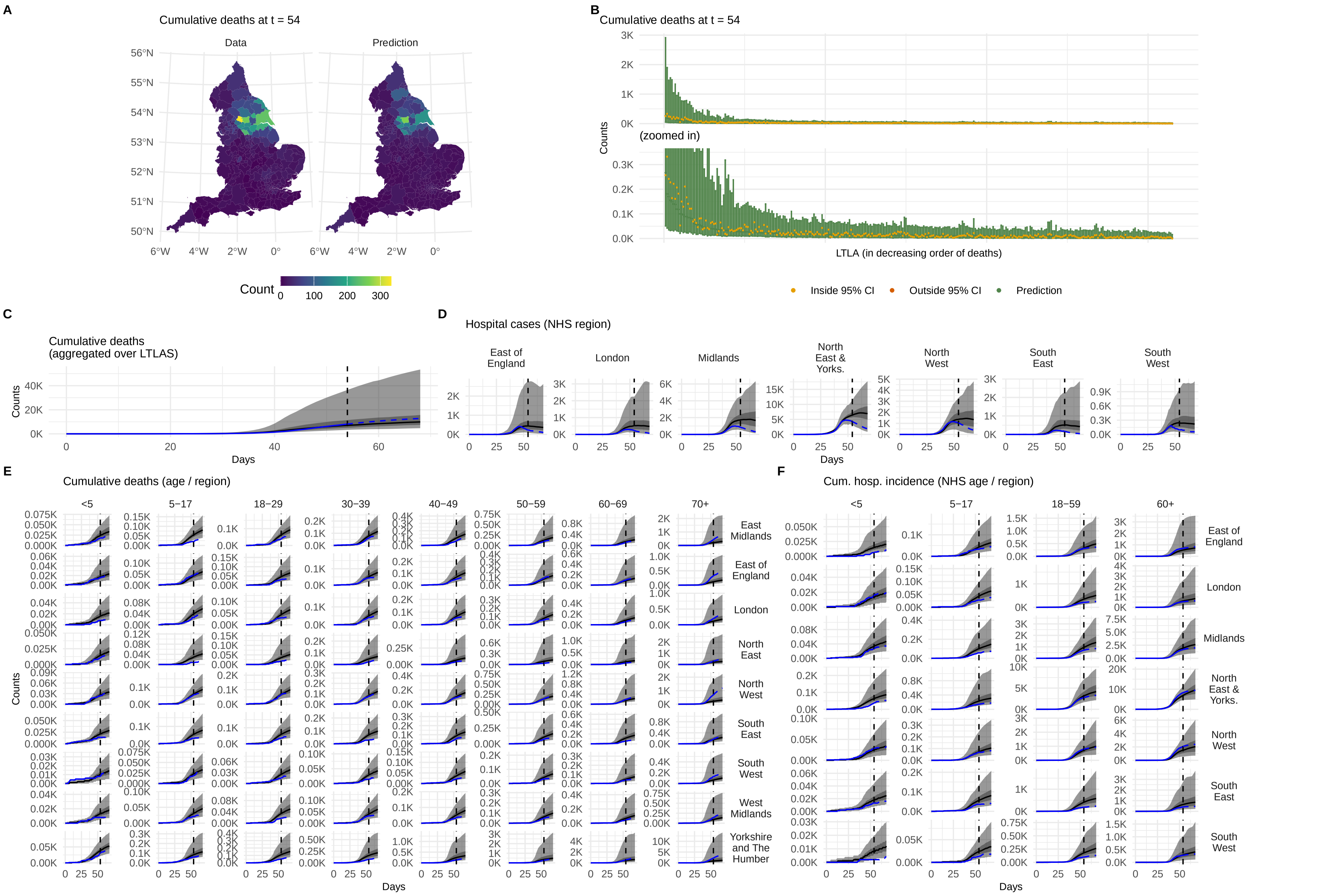


Figure D: Particle trajectory plots across the ensemble of design points at Wave 10 for the simulated outbreak. Plots identical to Figure 3 in the main text, except $y$-axes are allowed to vary in D–F to better examine model fits. Source for shapefiles: https://geoportal.statistics.gov.uk from the Office for National Statistics licensed under the Open Government Licence v.3.0. Contains OS data: Crown copyright and database right 2022.

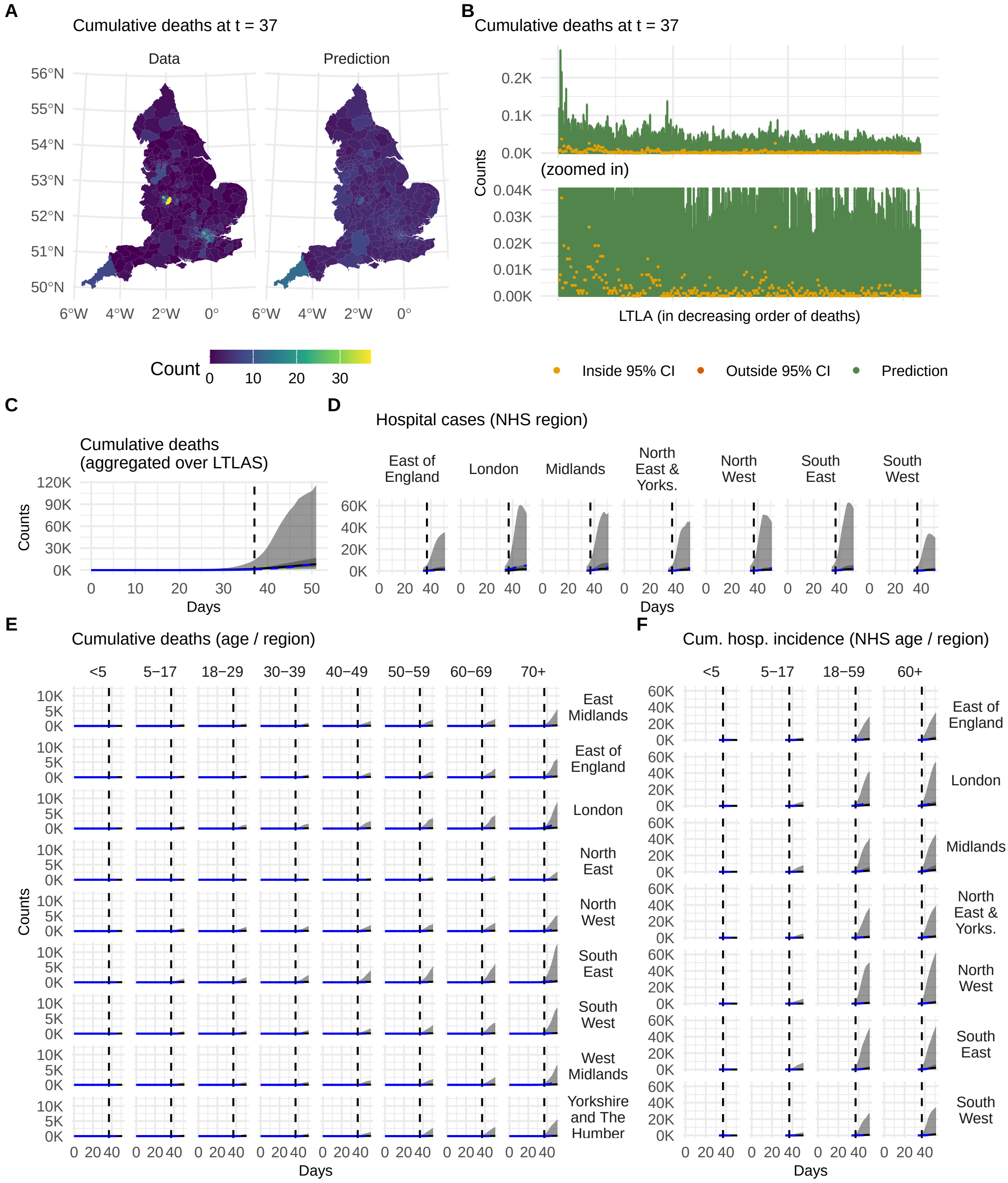


Figure E: Particle trajectory plots across the ensemble of design points at Wave 1 for the real UK data up to the first lockdown. A) Spatial (LTLA-level) plots of the mean number of deaths by day 37, for the data and the fitted model. B) Cumulative deaths at day 37 within each LTLA (ranked in decreasing order of predicted deaths). C) Cumulative deaths over time, aggregated over the 315 LTLAs with death data available. D) Hospital cases over time in each NHS region. E) Cumulative deaths over time in each age/region category. F) Cumulative hospital incidence over time by each NHS age/region category. In plot B) the green points are the predicted ensemble means, and the error bars are the 95% prediction intervals. For clarity we show a zoomed-in version of the plot also. The yellow and red points are the observed data coloured by whether they lie inside and outside of the prediction intervals respectively. In plots C–F, the blue dashed lines correspond to the observed data and the black solid lines to the mean trajectories from the particles taken across the ensemble. The ribbons correspond to 50% and 95% prediction intervals. A vertical dashed line corresponds to the end point of the observed data, such that trajectories before the line are generated from the particle filter, and trajectories to the right of the line are simulated forecasts from the model. Source for shapefiles: https://geoportal.statistics.gov.uk from the Office for National Statistics licensed under the Open Government Licence v.3.0. Contains OS data: Crown copyright and database right 2022.

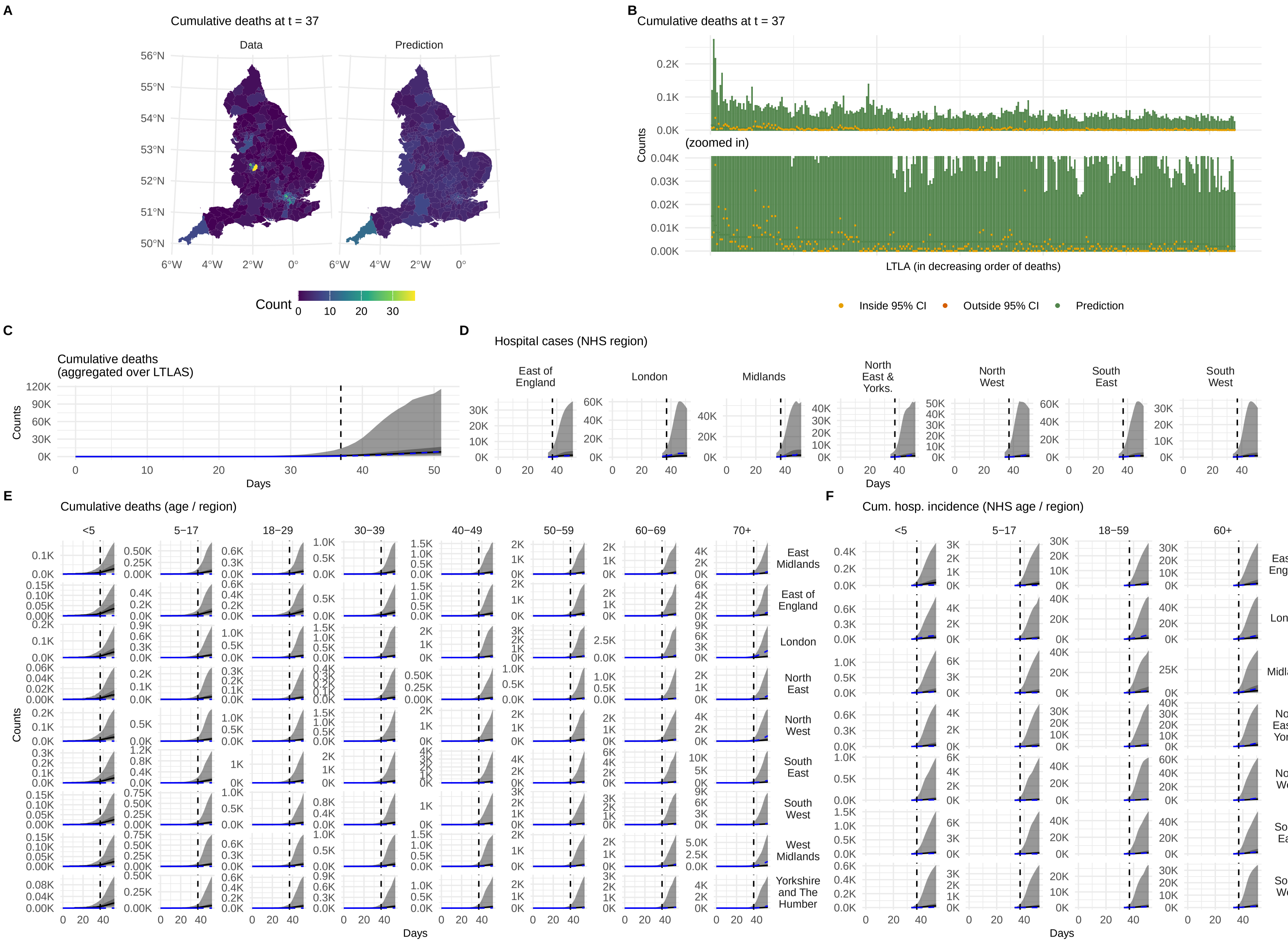


Figure F: Particle trajectory plots across the ensemble of design points at Wave 1 for the real UK data up to the first lockdown. Plots identical to Figure E except $y$-axes are allowed to vary in D–F to better examine model fits. Source for shapefiles: https://geoportal.statistics.gov.uk from the Office for National Statistics licensed under the Open Government Licence v.3.0. Contains OS data: Crown copyright and database right 2022.

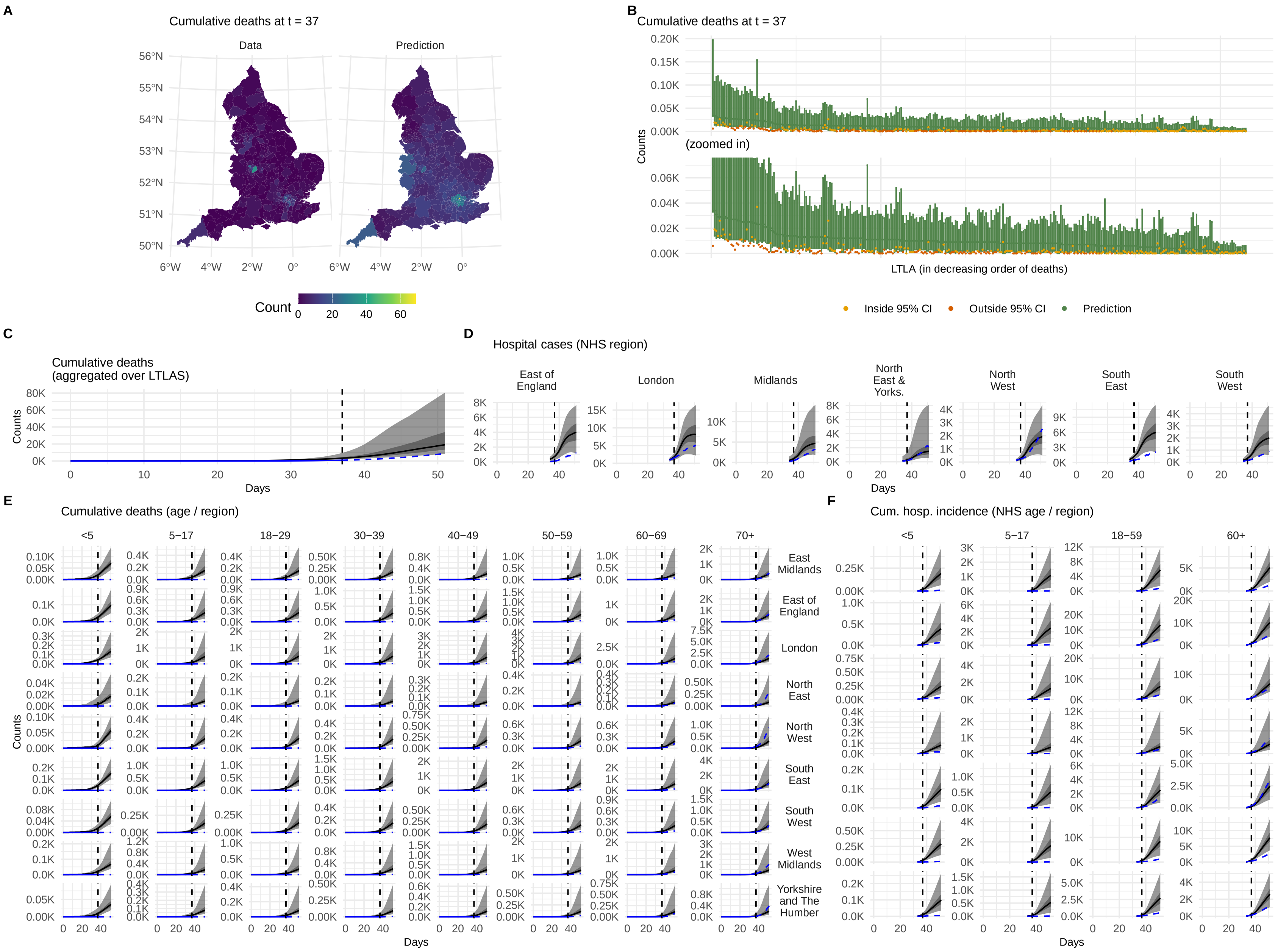


Figure G: Particle trajectory plots across the ensemble of design points at Wave 10 for the real UK data up to the first lockdown. Plots identical to Figure 4 in the main text, except $y$-axes are allowed to vary in D–F to better examine model fits. Source for shapefiles: https://geoportal.statistics.gov.uk from the Office for National Statistics licensed under the Open Government Licence v.3.0. Contains OS data: Crown copyright and database right 2022.

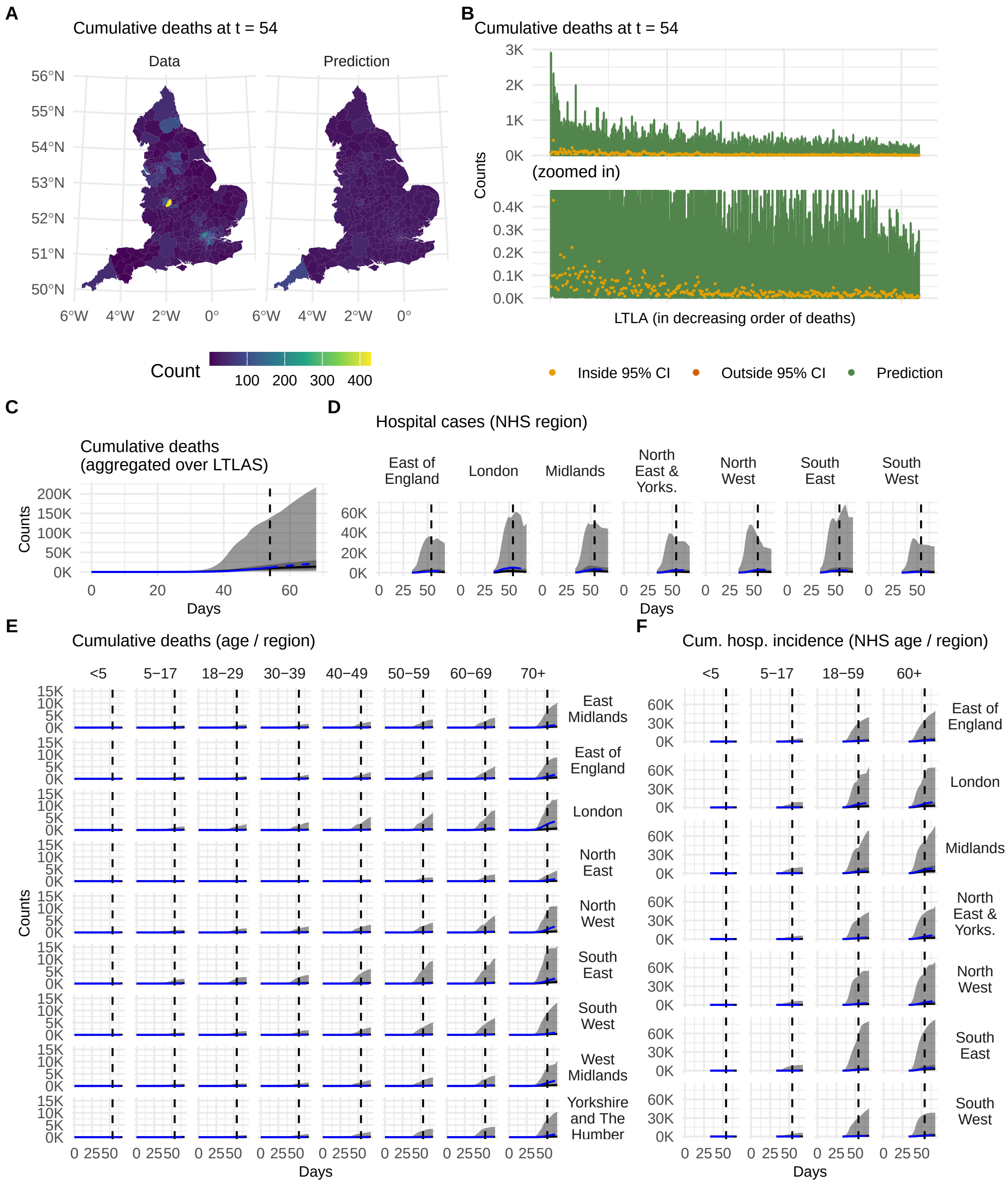


Figure H: Particle trajectory plots across the ensemble of design points at Wave 1 for the real UK data beyond the first lockdown. A) Spatial (LTLA-level) plots of the mean number of deaths by day 54, for the data and the fitted model. B) Cumulative deaths at day 54 within each LTLA (ranked in decreasing order of predicted deaths). C) Cumulative deaths over time, aggregated over the 315 LTLAs with death data available. D) Hospital cases over time in each NHS region. E) Cumulative deaths over time in each age/region category. F) Cumulative hospital incidence over time by each NHS age/region category. In plot B) the green points are the predicted ensemble means, and the error bars are the 95% prediction intervals. For clarity we show a zoomed-in version of the plot also. The yellow and red points are the observed data coloured by whether they lie inside and outside of the prediction intervals respectively. In plots C–F, the blue dashed lines correspond to the observed data and the black solid lines to the mean trajectories from the particles taken across the ensemble. The ribbons correspond to 50% and 95% prediction intervals. A vertical dashed line corresponds to the end point of the observed data, such that trajectories before the line are generated from the particle filter, and trajectories to the right of the line are simulated forecasts from the model. Source for shapefiles: https://geoportal.statistics.gov.uk from the Office for National Statistics licensed under the Open Government Licence v.3.0. Contains OS data: Crown copyright and database right 2022.

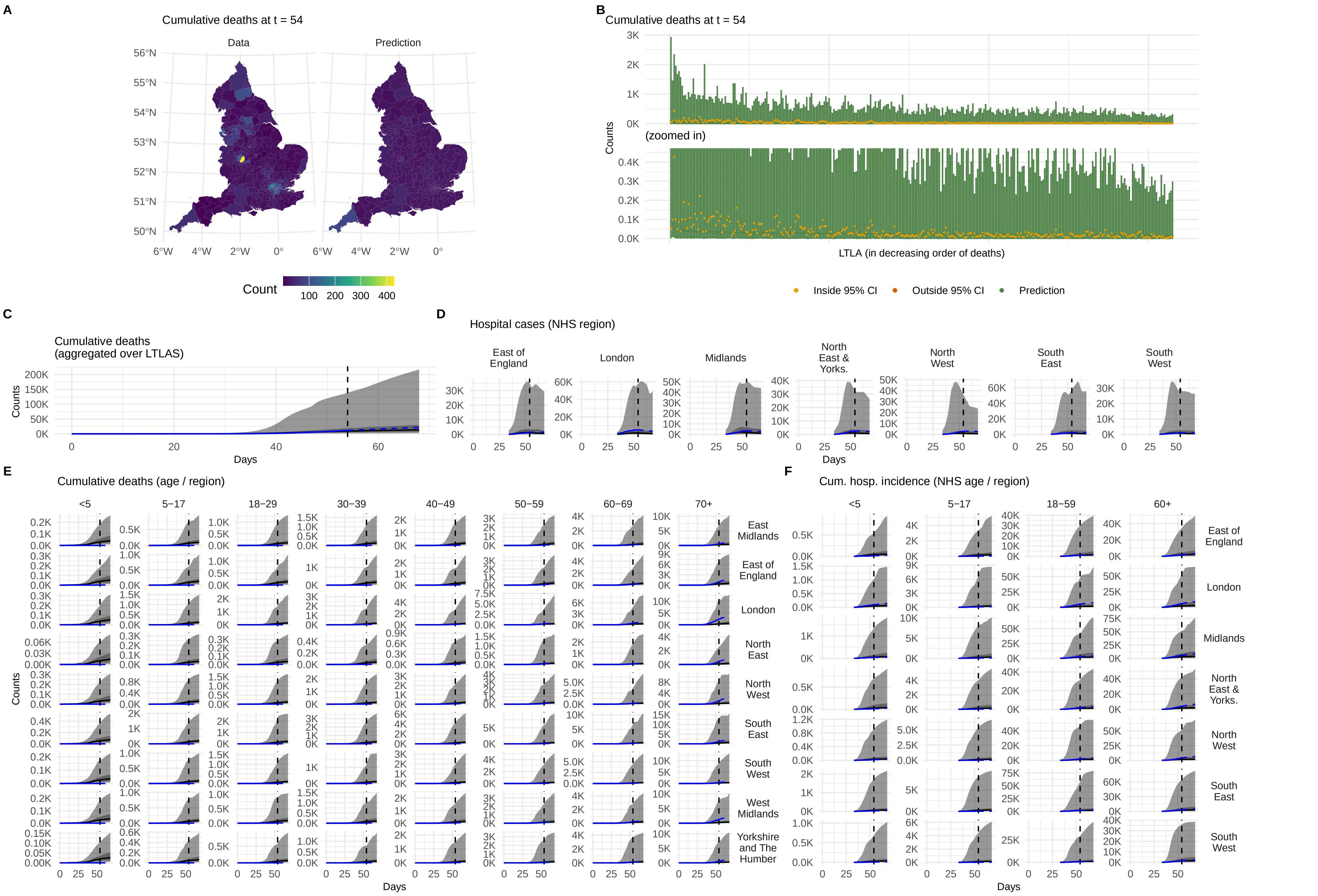


Figure I: Particle trajectory plots across the ensemble of design points at Wave 1 for the real UK data beyond the first lockdown. Plots identical to Figure H except $y$-axes are allowed to vary in D–F to better examine model fits. Source for shapefiles: https://geoportal.statistics.gov.uk from the Office for National Statistics licensed under the Open Government Licence v.3.0. Contains OS data: Crown copyright and database right 2022.

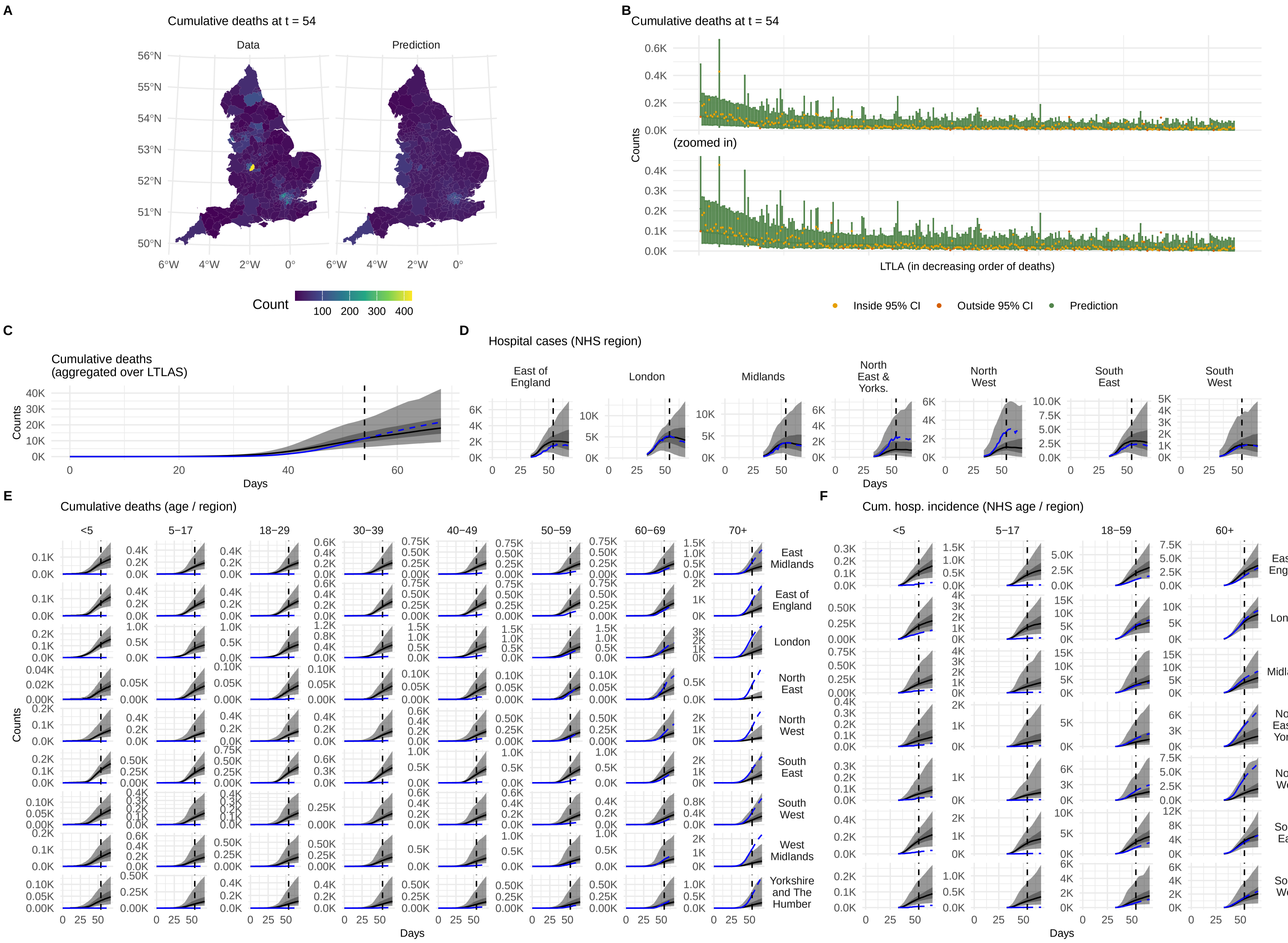


Figure J: Particle trajectory plots across the ensemble of design points at Wave 10 for the real UK data beyond the first lockdown. Plots identical to Figure 5 in the main text, except $y$-axes are allowed to vary in D–F to better examine model fits. Source for shapefiles: https://geoportal.statistics.gov.uk from the Office for National Statistics licensed under the Open Government Licence v.3.0. Contains OS data: Crown copyright and database right 2022.

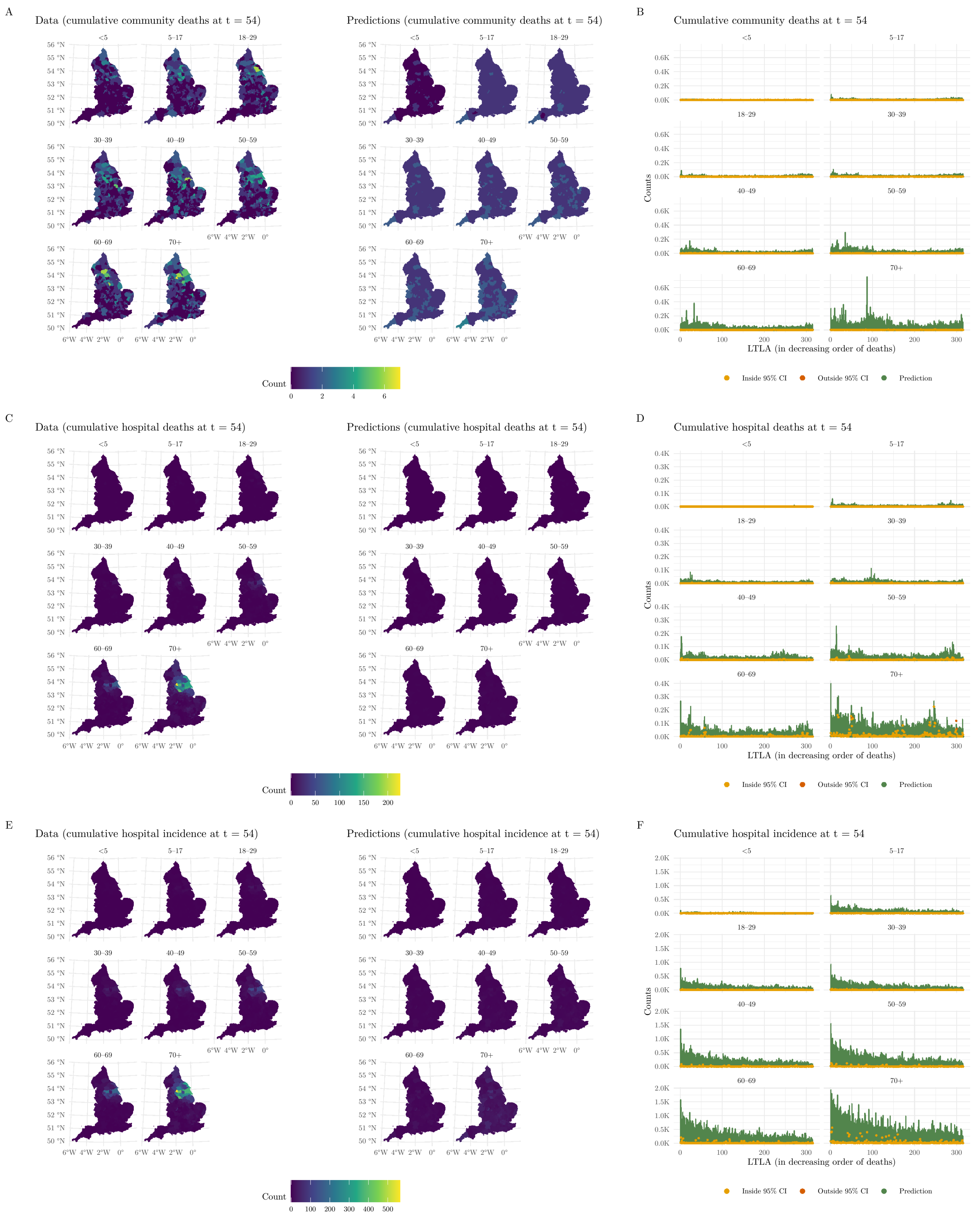


Figure K: Particle trajectory plots across the ensemble of design points at Wave 1 for the simulated outbreak assuming data are available at the resolution of the model. Spatial (LTLA-level) plots of the data and the fitted model, are given for A) the mean number of community deaths, C) the mean number of hospital deaths, and E) the cumulative hospital incidence by day 54. Similarly, predicted and observed counts within each LTLA (ranked in decreasing order of predicted deaths) are given for B) the mean number of community deaths, D) the mean number of hospital deaths, and F) the cumulative hospital incidence by day 54 in each age category. In plots B), D) and F) the green points are the predicted ensemble means, and the error bars are the 95% prediction intervals. The yellow and red points are the observed data coloured by whether they lie inside and outside of the prediction intervals respectively. Source for shapefiles: https://geoportal.statistics.gov.uk from the Office for National Statistics licensed under the Open Government Licence v.3.0. Contains OS data: Crown copyright and database right 2022.

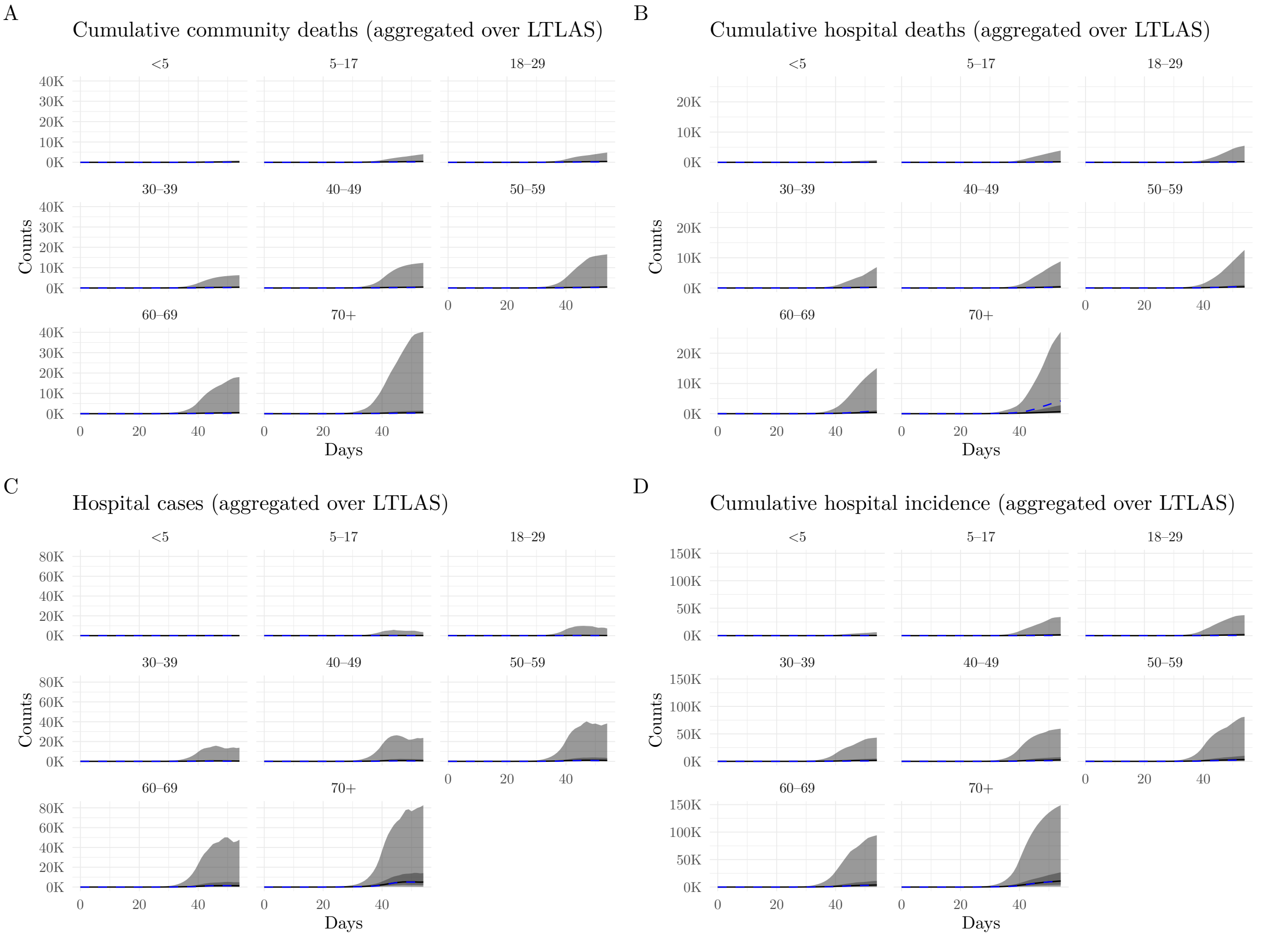


Figure L: Particle trajectory plots across the ensemble of design points at Wave 1 for the simulated outbreak assuming data are available at the resolution of the model. Counts over time, by each age category aggregated over the 315 LTLAs with death data available are given for A) cumulative community deaths, B) cumulative hospital deaths, C) hospital cases and D) cumulative hospital incidence. The blue dashed lines correspond to the observed data and the black solid lines to the mean trajectories from the particles taken across the ensemble. The ribbons correspond to 50% and 95% prediction intervals.

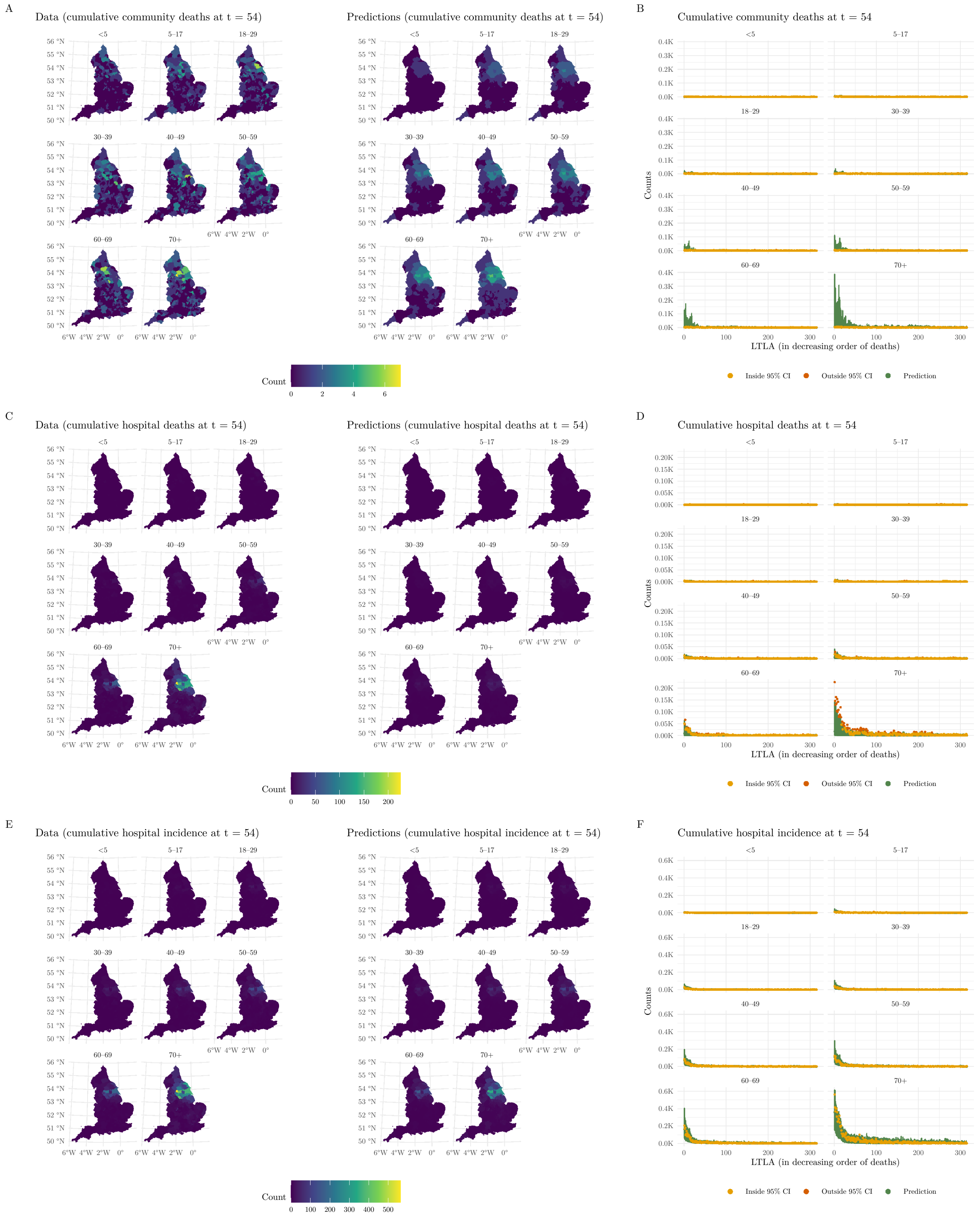


Figure M: Particle trajectory plots across the ensemble of design points at Wave 10 for the simulated outbreak assuming data are available at the resolution of the model. Spatial (LTLA-level) plots of the data and the fitted model, are given for A) the mean number of community deaths, C) the mean number of hospital deaths, and E) the cumulative hospital incidence by day 54. Similarly, predicted and observed counts within each LTLA (ranked in decreasing order of predicted deaths) are given for B) the mean number of community deaths, D) the mean number of hospital deaths, and F) the cumulative hospital incidence by day 54 in each age category. In plots B), D) and F) the green points are the predicted ensemble means, and the error bars are the 95% prediction intervals. The yellow and red points are the observed data coloured by whether they lie inside and outside of the prediction intervals respectively. Source for shapefiles: https://geoportal.statistics.gov.uk from the Office for National Statistics licensed under the Open Government Licence v.3.0. Contains OS data: Crown copyright and database right 2022.

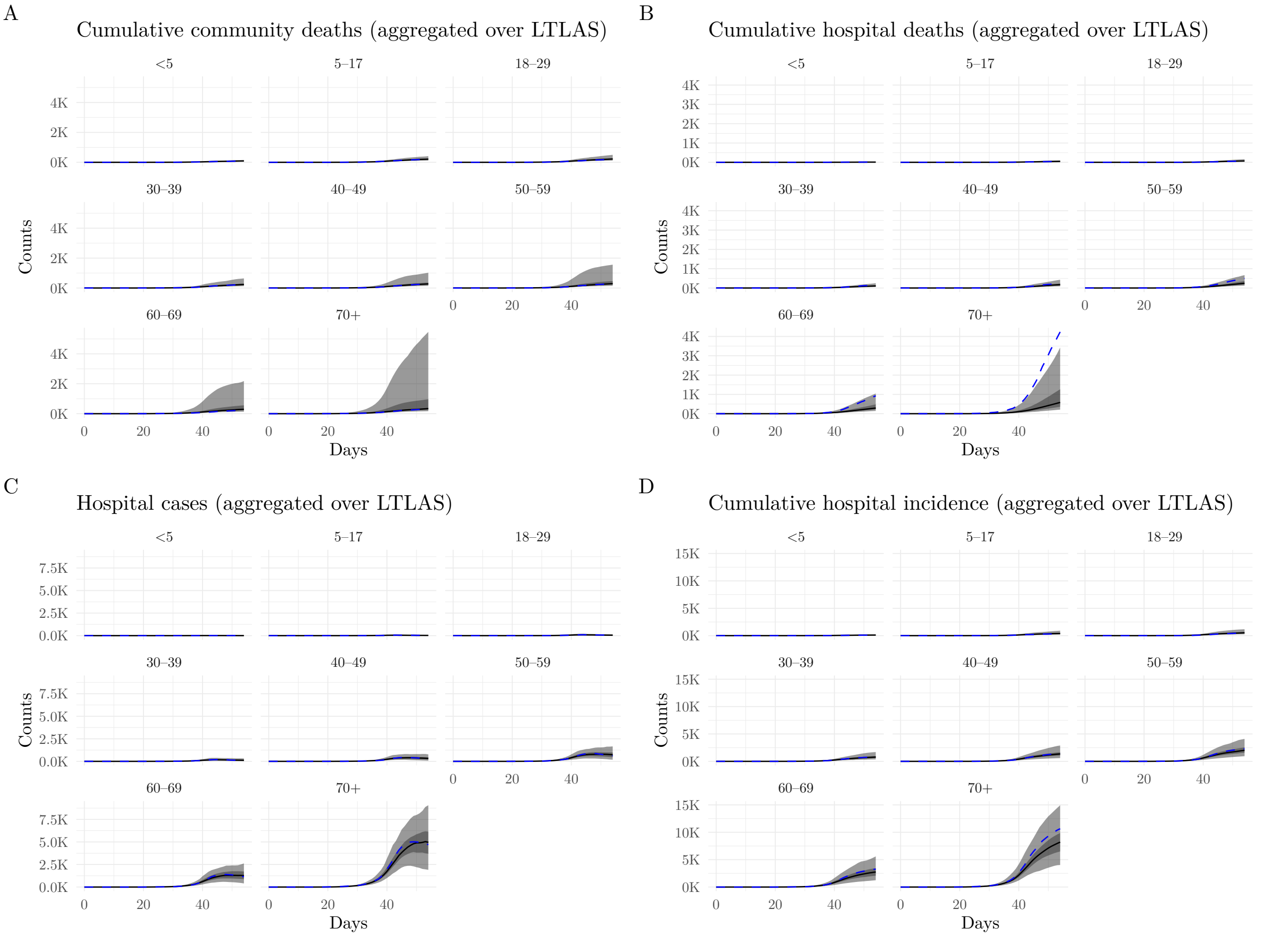


Figure N: Particle trajectory plots across the ensemble of design points at Wave 10 for the simulated outbreak assuming data are available at the resolution of the model. Counts over time, by each age category aggregated over the 315 LTLAs with death data available are given for A) cumulative community deaths, B) cumulative hospital deaths, C) hospital cases and D) cumulative hospital incidence. The blue dashed lines correspond to the observed data and the black solid lines to the mean trajectories from the particles taken across the ensemble. The ribbons correspond to 50% and 95% prediction intervals.

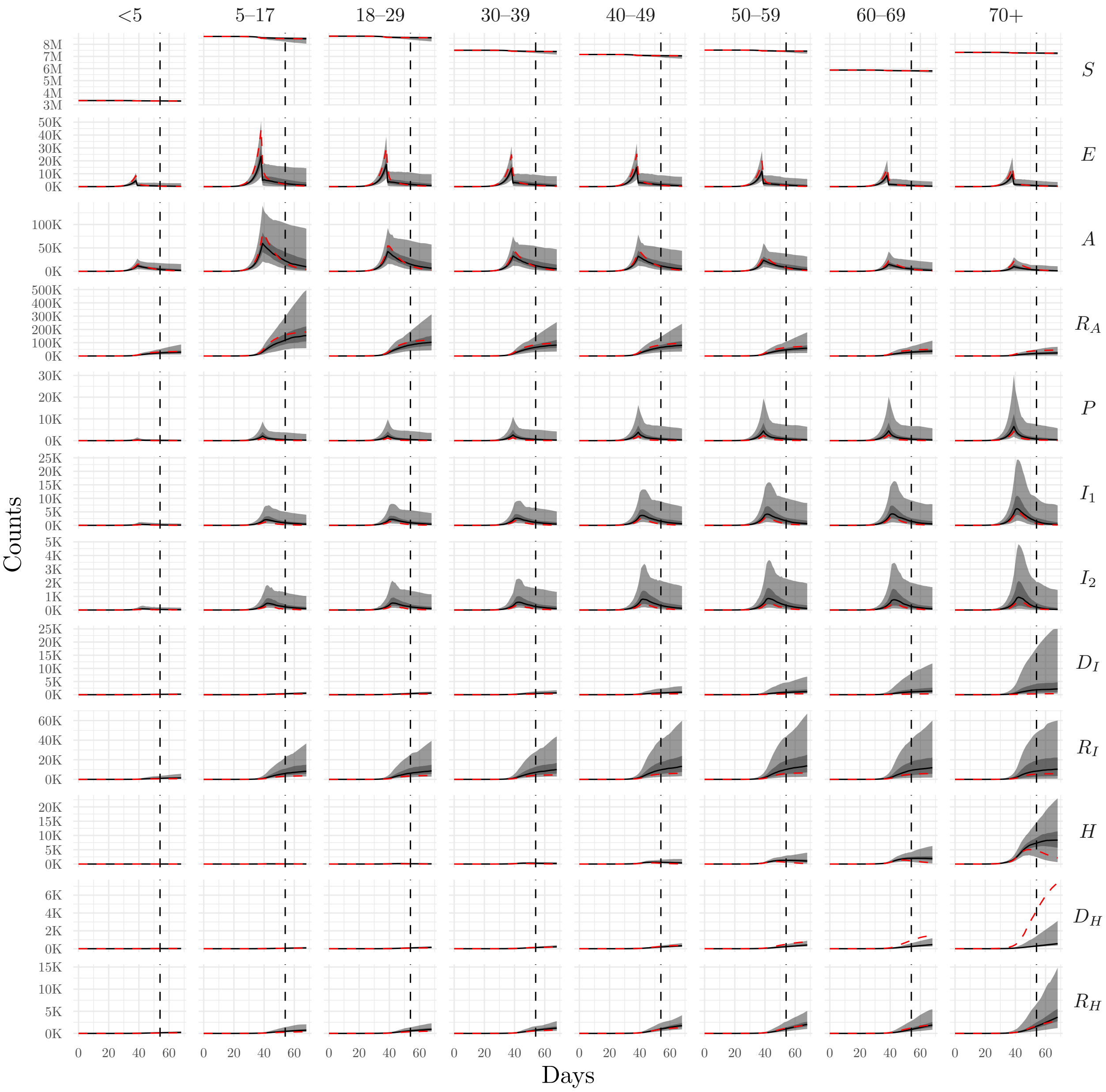


Figure O: Particle trajectory plots for the hidden states across the ensemble of design points at Wave 10 for the simulated outbreak. The red lines correspond to the true trajectories, the black lines to the mean trajectories from the particles taken across the ensemble, and the ribbons correspond to 50% and 95% prediction intervals. A vertical dashed line corresponds to the end point of the observed data, such that trajectories before the line are generated from the particle filter, and trajectories to the right of the line are simulated forecasts from the model.

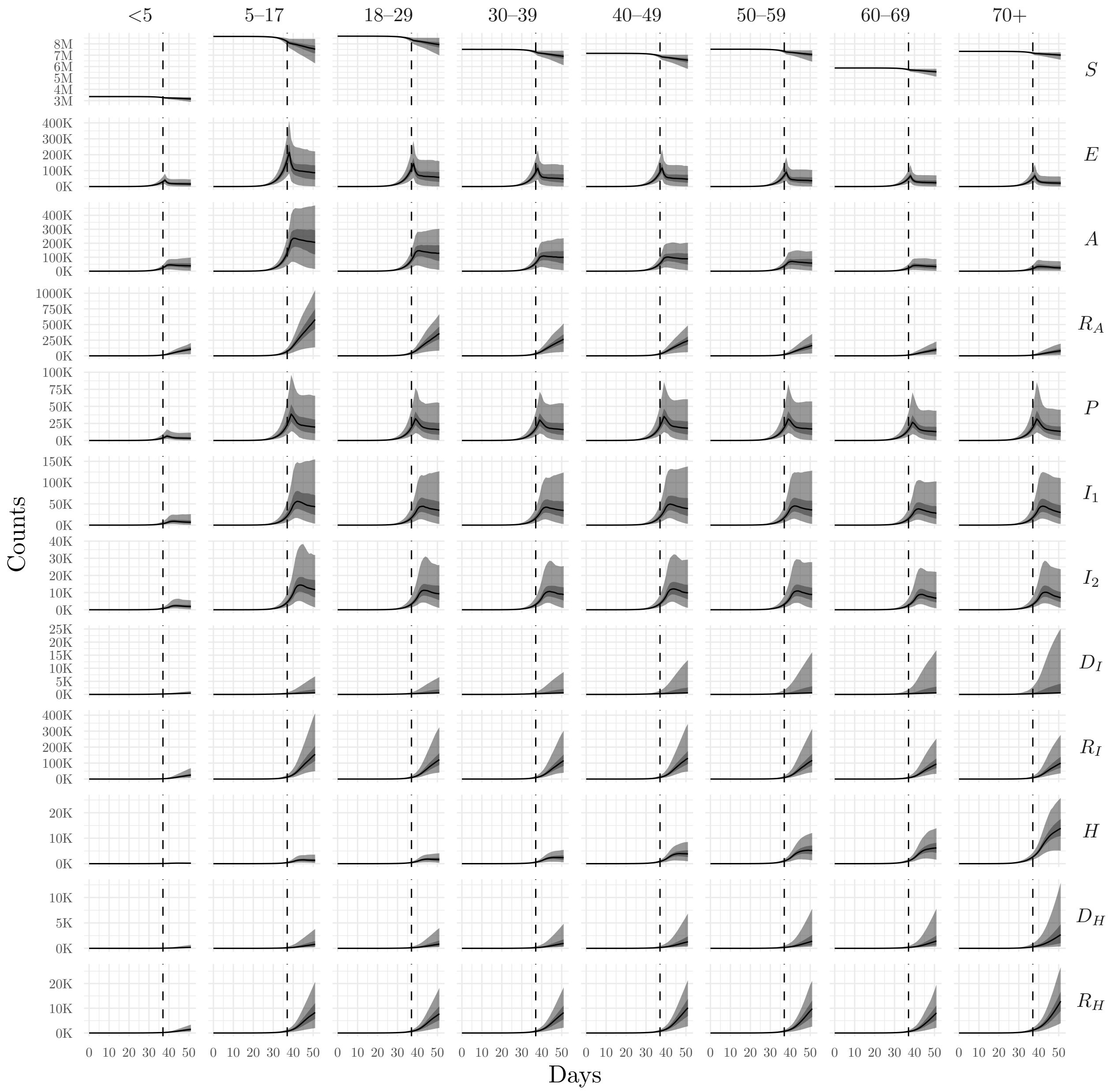


Figure P: Particle trajectory plots for the hidden states across the ensemble of design points at Wave 10 for the real UK data up to the first lockdown. The black lines are the mean trajectories from the particles taken across the ensemble, and the ribbons correspond to 50% and 95% prediction intervals. A vertical dashed line corresponds to the end point of the observed data, such that trajectories before the line are generated from the particle filter, and trajectories to the right of the line are simulated forecasts from the model.

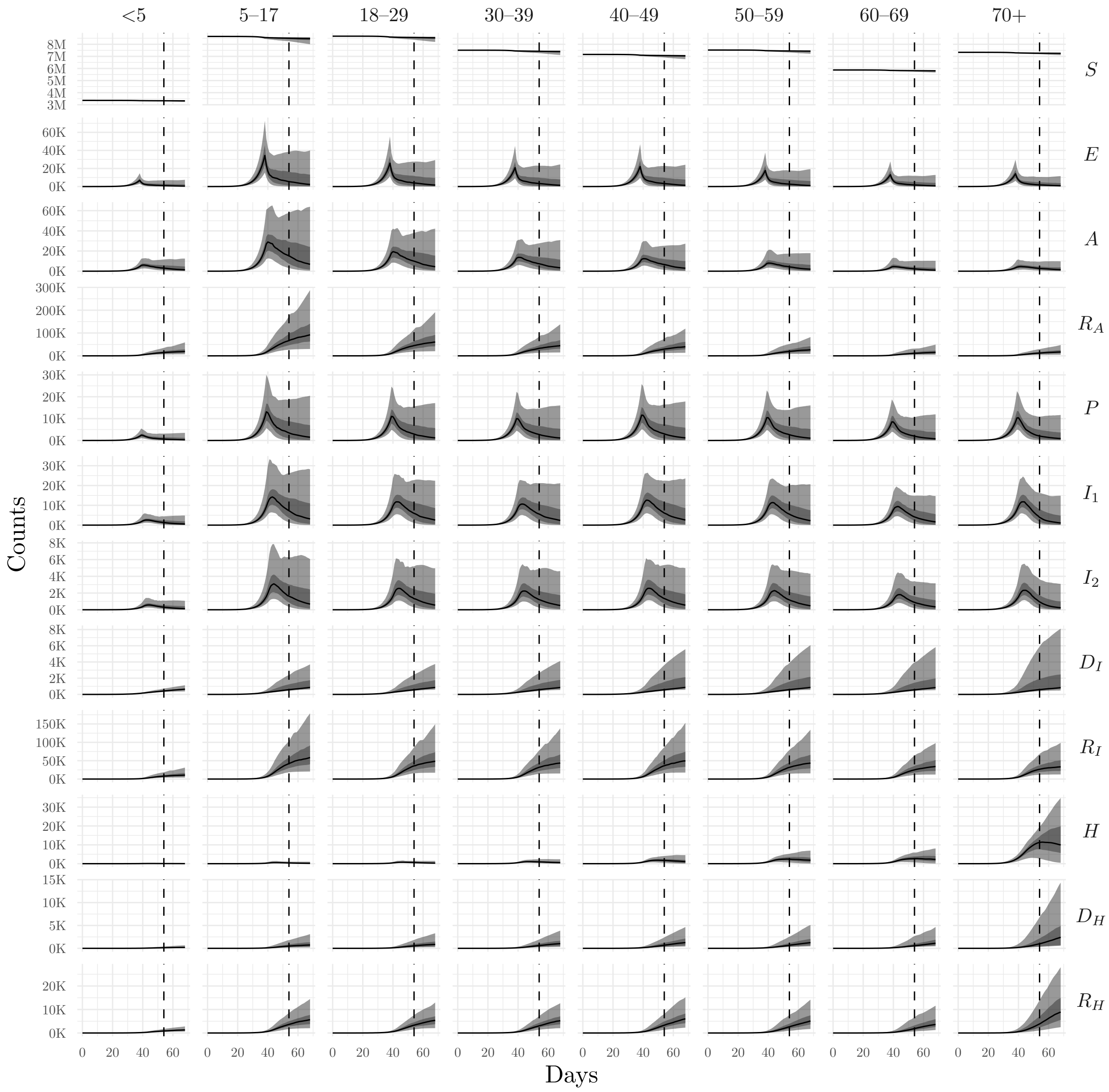


Figure Q: Particle trajectory plots for the hidden states across the ensemble of design points at Wave 10 for the real UK data beyond the first lockdown. The black lines are the mean trajectories from the particles taken across the ensemble, and the ribbons correspond to 50% and 95% prediction intervals. A vertical dashed line corresponds to the end point of the observed data, such that trajectories before the line are generated from the particle filter, and trajectories to the right of the line are simulated forecasts from the model.

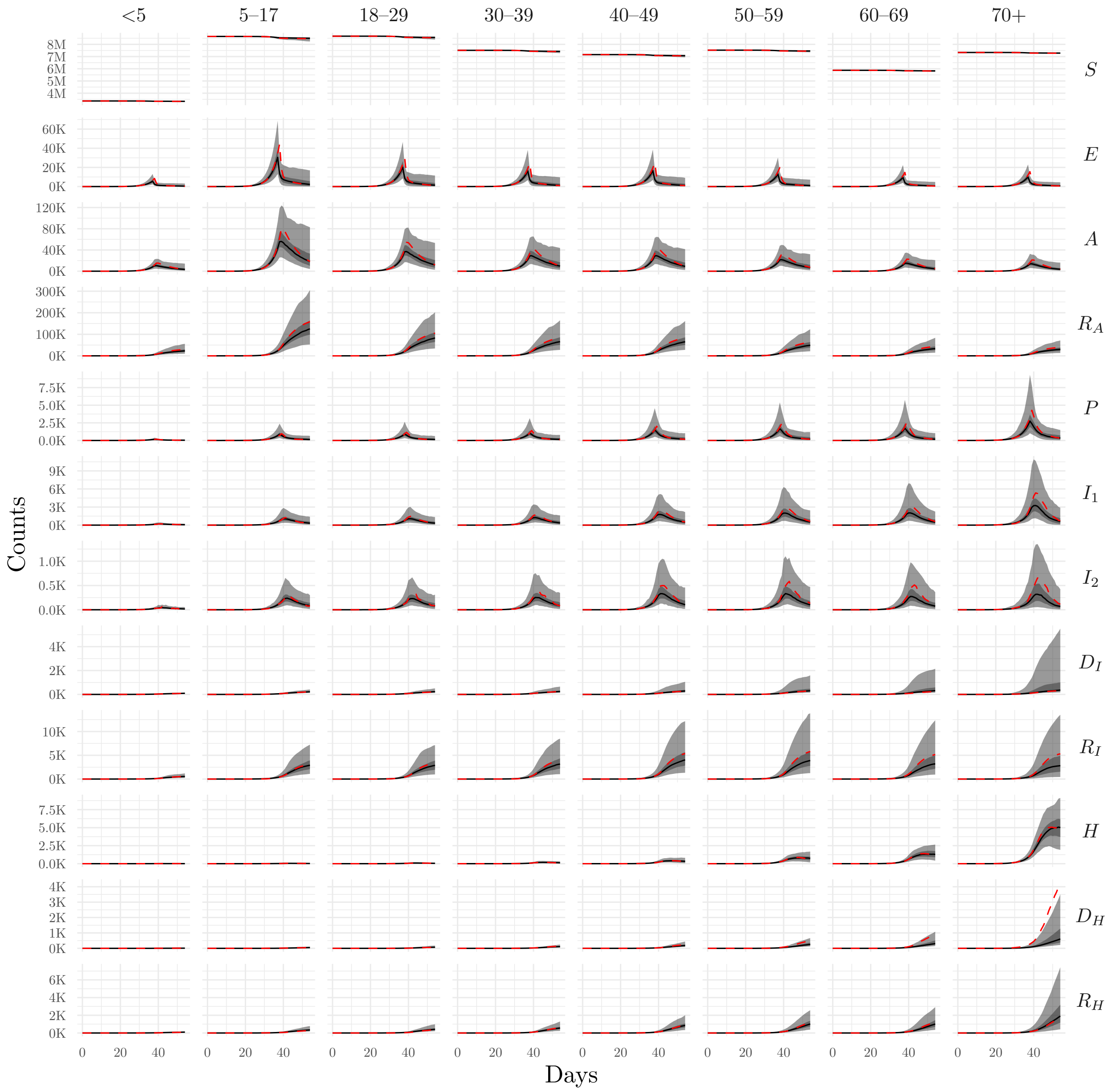


Figure R: Particle trajectory plots for the hidden states across the ensemble of design points at Wave 10 for the simulated outbreak assuming data are available at the resolution of the model. The red lines correspond to the true trajectories, the black lines to the mean trajectories from the particles taken across the ensemble, and the ribbons correspond to 50% and 95% prediction intervals.

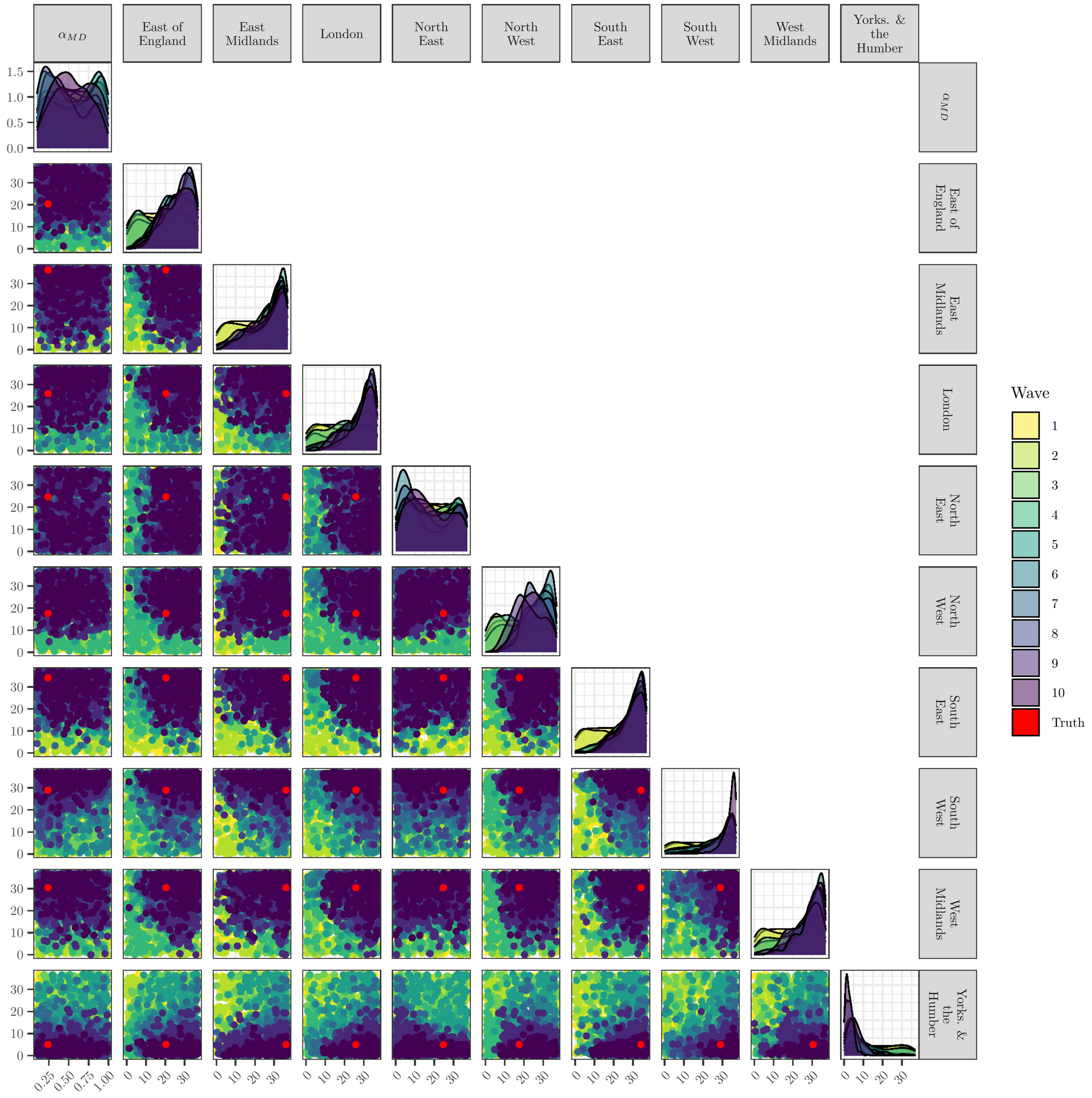


Figure S: Inputs for Waves 1–10 for the $t_{r,\text{MD}}$ parameters from a model fitted to a simulated outbreak. The red points correspond to the true parameter values.

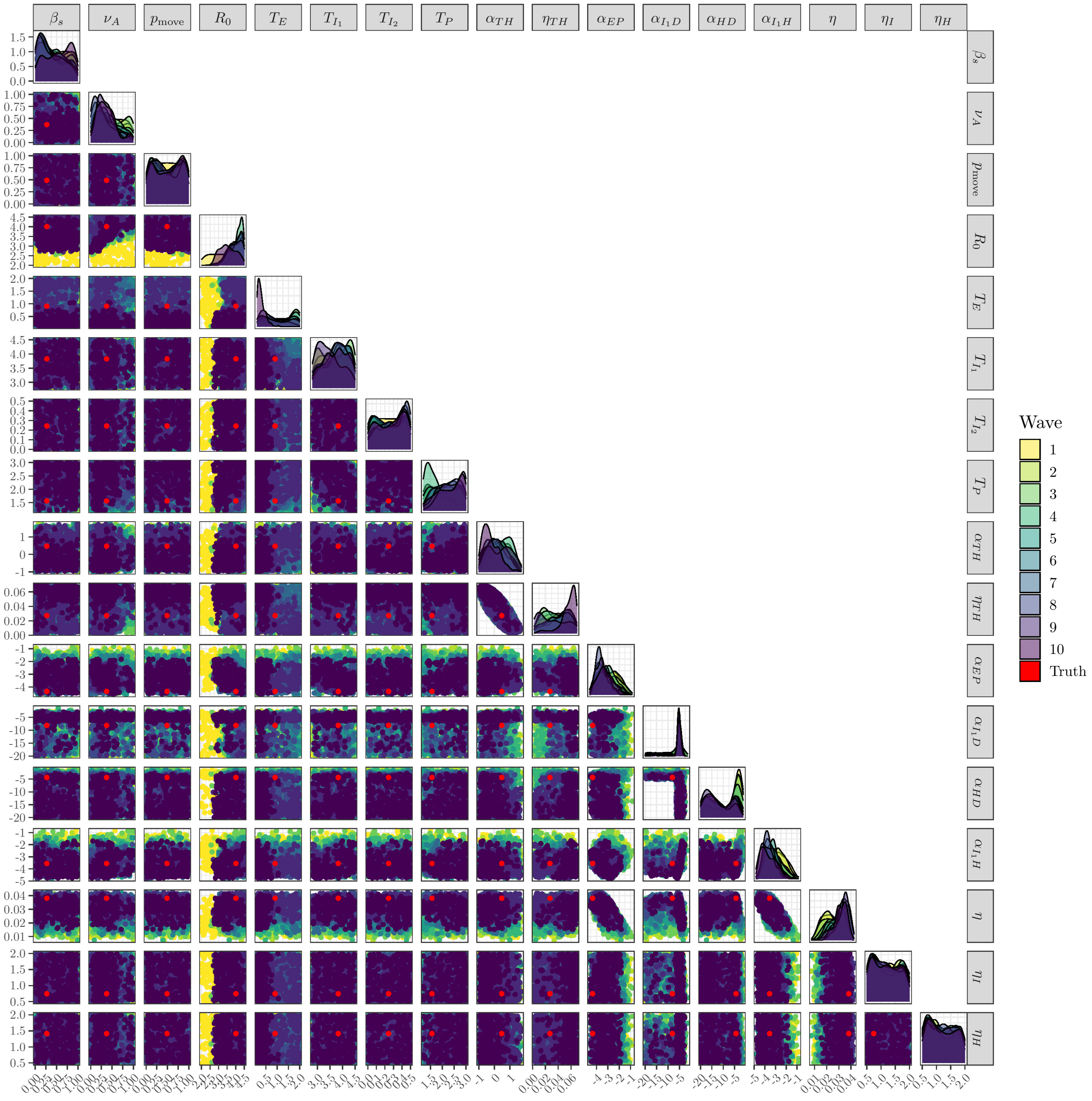


Figure T: Inputs for Waves 1–10 for all parameters except the $t_{r,\text{MD}}$ parameters from a model fitted to a simulated outbreak. The red points correspond to the true parameter values.

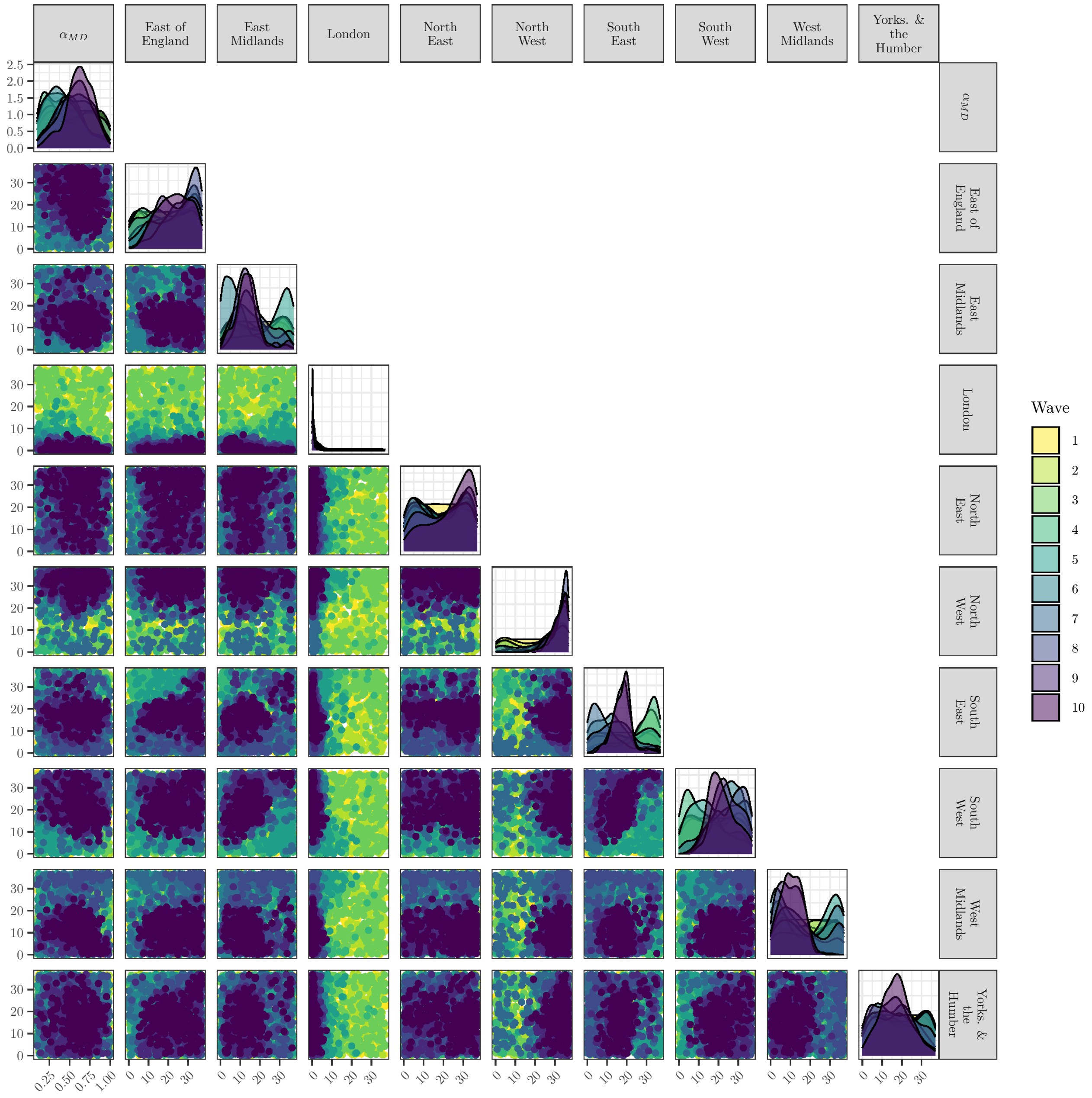


Figure U: Inputs for Waves 1–10 for the $t_{r,\mathrm{MD}}$ parameters from a model fitted to the real UK data up to the first lockdown.

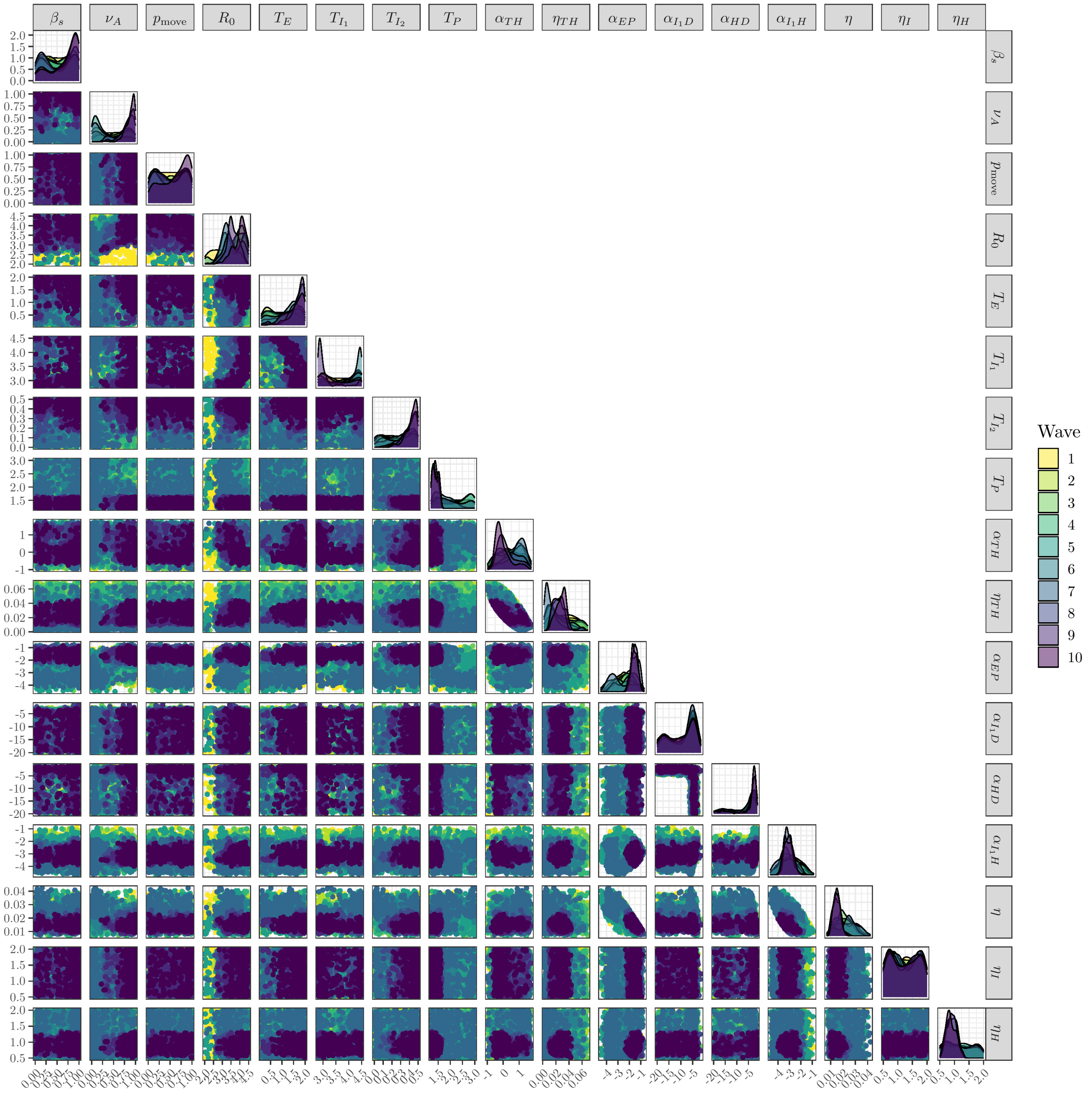

Figure V: Inputs for Waves 1–10 for all parameters except the $t_{r,\mathrm{MD}}$ parameters from a model fitted to the real UK data up to the first lockdown.

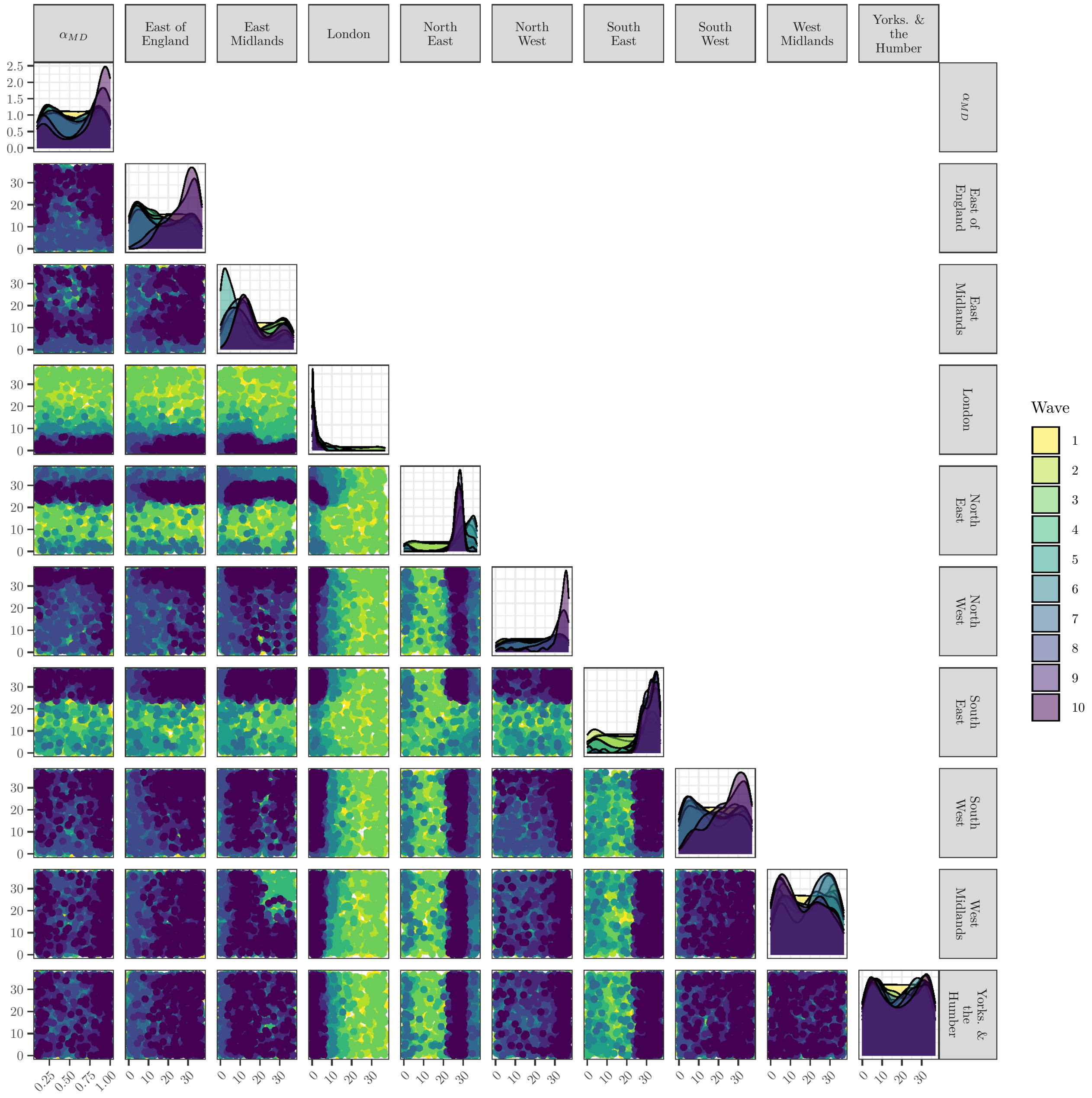


Figure W: Inputs for Waves 1–10 for the $t_{r,\mathrm{MD}}$ parameters from a model fitted to the real UK data beyond the first lockdown.

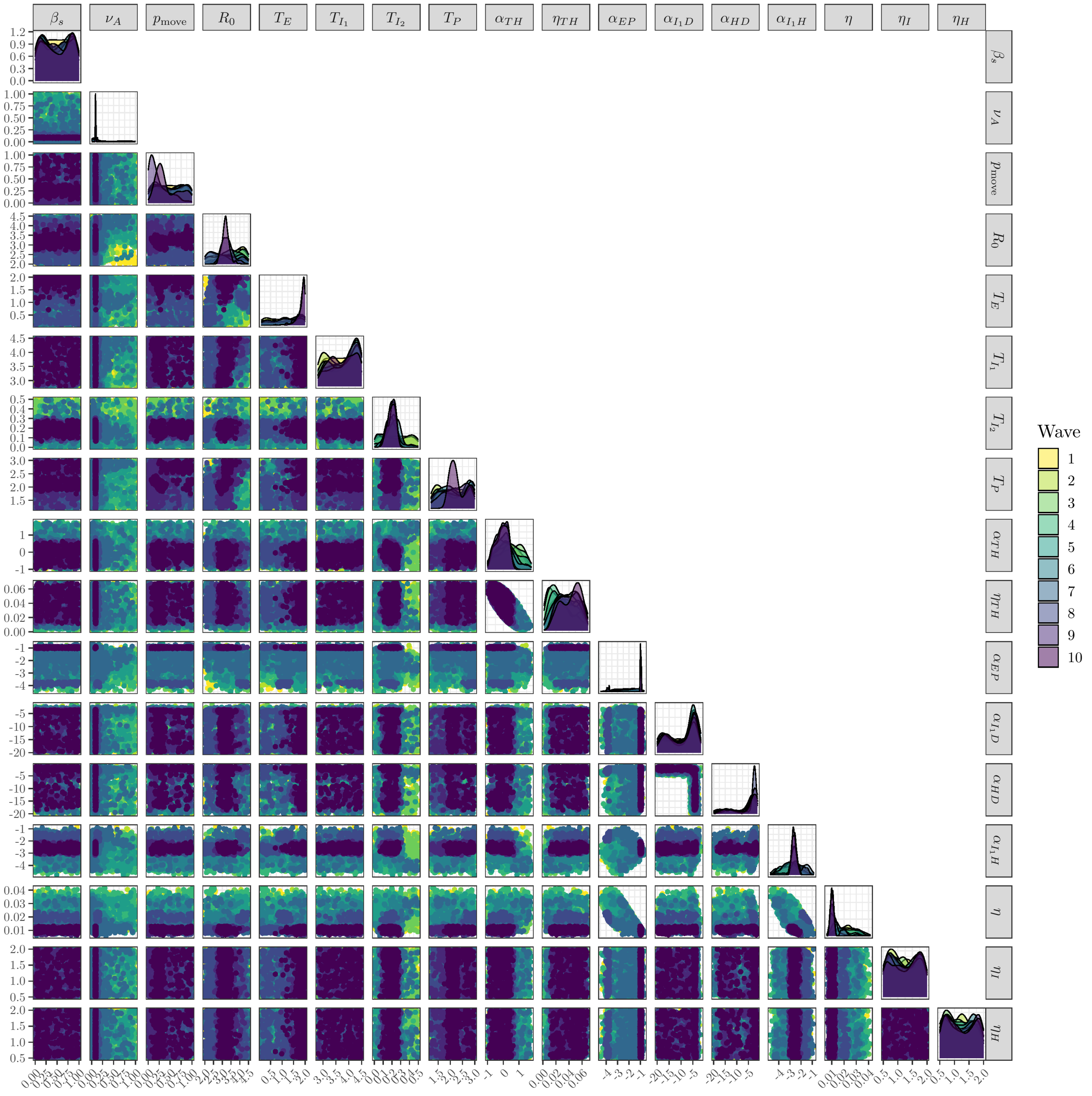


Figure X: Inputs for Waves 1–10 for all parameters except the $t_{r,\text{MD}}$ parameters from a model fitted to the real UK data beyond the first lockdown.

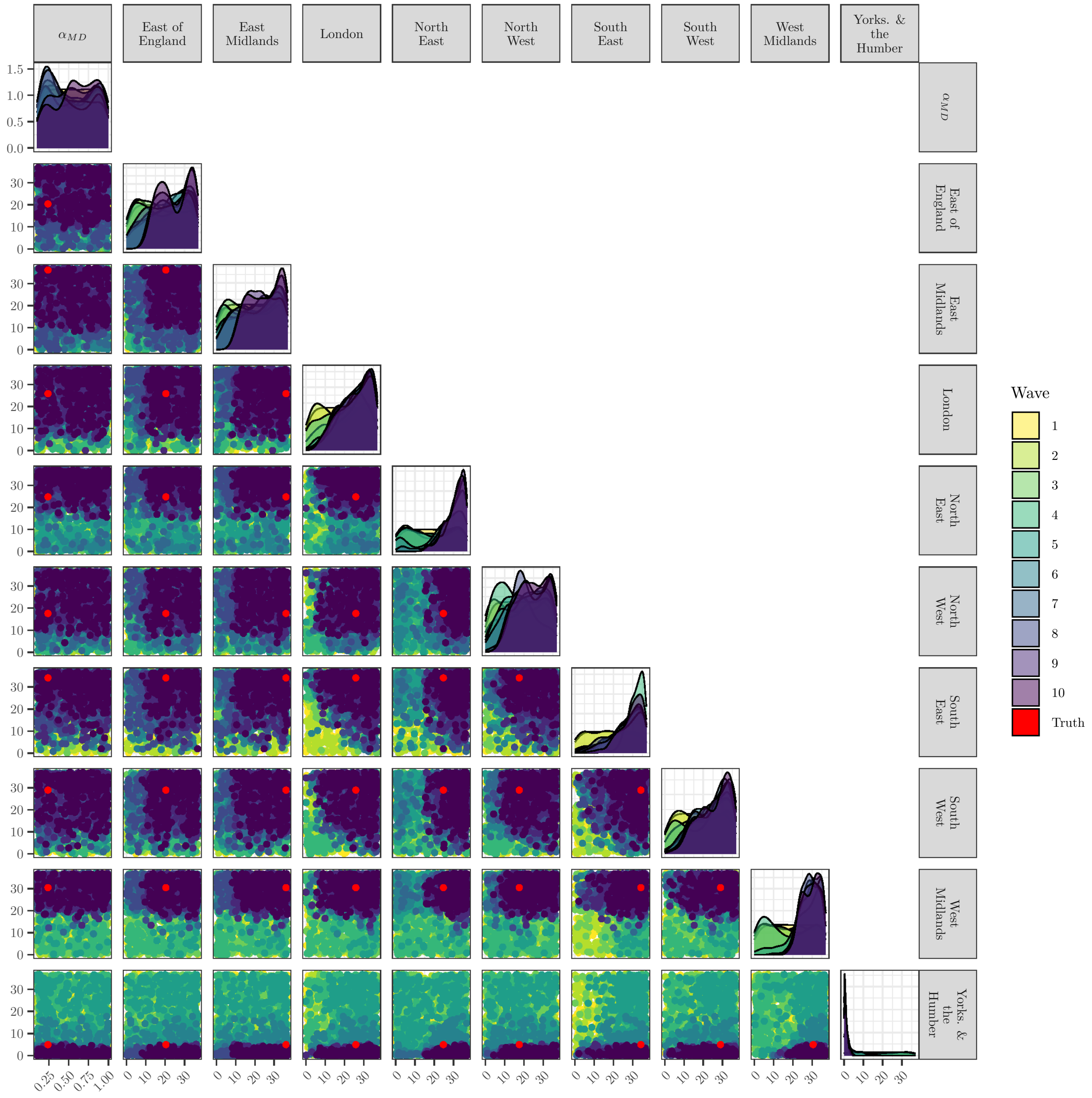


Figure Y: Inputs for Waves 1–10 for the $t_{r,\mathrm{MD}}$ parameters for a model fitted to the simulated data assuming data are available at the resolution of the model. The red points correspond to the true parameter values.

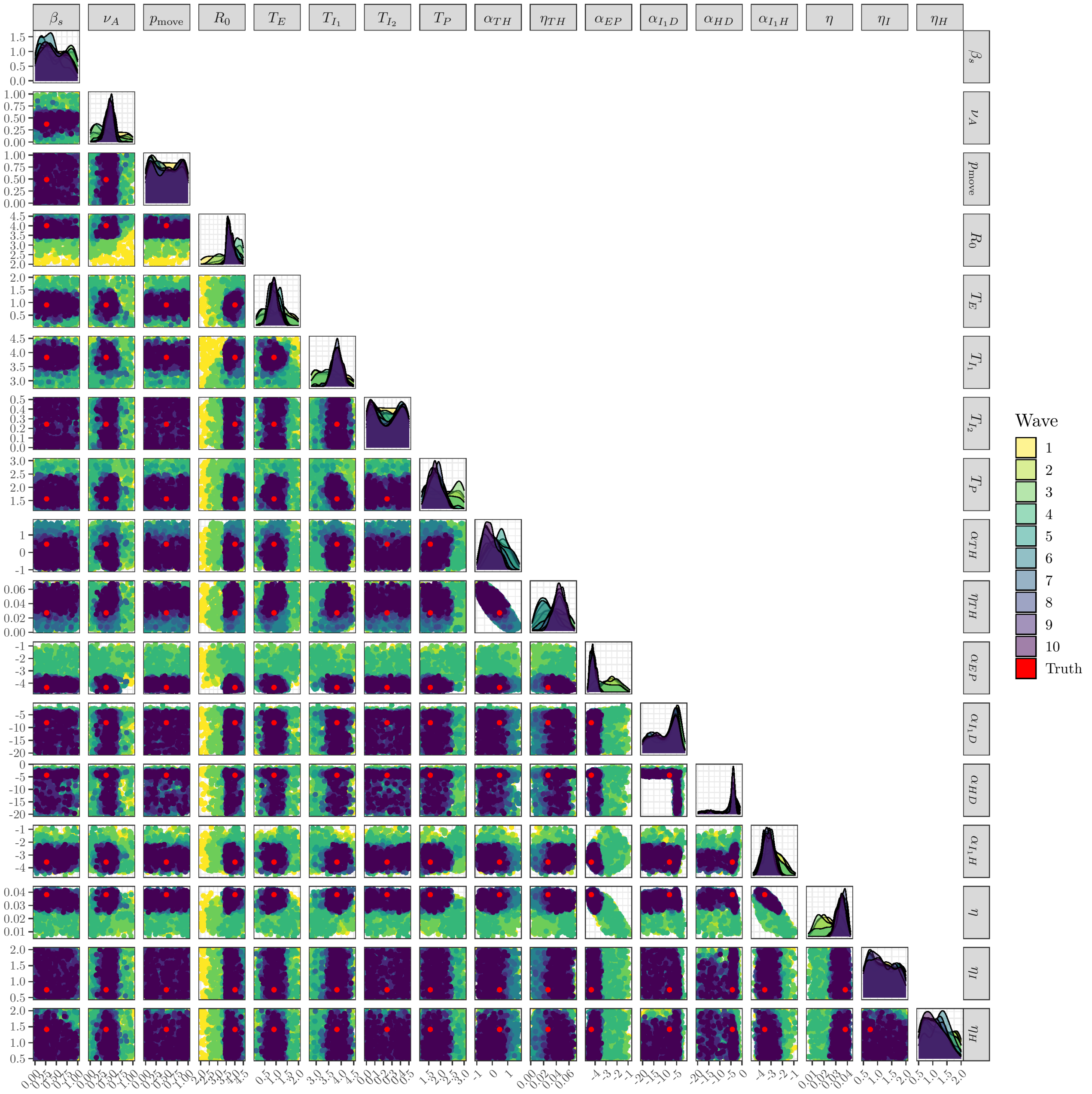


Figure Z: Inputs for Waves 1–10 for all parameters except the $t_{r,\mathrm{MD}}$ parameters for a model fitted to the simulated data assuming data are available at the resolution of the model. The red points correspond to the true parameter values.

## References


James Balamuta, Thijs van den Berg, and Ralf Stubner. *sitmo: Parallel Pseudo Random Number Generator (PPRNG) 'sitmo' Header Files*, 2021. URL `https://github.com/coatless-rpkg/sitmo`. R package version 2.0.2, https://github.com/coatless-rpkg/sitmo.

Tyson Barrett, Matt Dowle, Arun Srinivasan, Jan Gorecki, Michael Chirico, Toby Hocking, Benjamin Schwendinger, and Ivan Krylov. *data.table: Extension of 'data.frame'*, 2025. URL `https://r-datatable.com`. R package version 1.17.99, https://Rdatatable.gitlab.io/data.table, https://github.com/Rdatatable/data.table.

Henrik Bengtsson. *R.utils: Various Programming Utilities*, 2025. URL `https://henrikbengtsson.github.io/R.utils/`. R package version 2.13.0, https://henrikbengtsson.github.io/R.utils/.

Rob Carnell. *lhs: Latin Hypercube Sampless*, 2024. URL `https://github.com/bertcarnell/lhs`. R package version 1.2.0, https://github.com/bertcarnell/lhs.

Perry de Valpine, Daniel Turek, Christopher Paciorek, Cliff Anderson-Bergman, Duncan Temple Lang, and Ras Bodik. Programming with models: writing statistical algorithms for general model structures with NIMBLE. *Journal of Computational and Graphical Statistics*, 26:403–413, 2017. doi: 10.1080/10618600.2016.1172487.

O. Diekmann, J. A. P. Heesterbeek, and J. A. J. Metz. On the definition and the computation of the basic reproduction ratio $R_0$ in models for infectious diseases in heterogeneous populations. *Journal of Mathematical Biology*, 28(4):365–382, 1990. doi: 10.1007/BF00178324.

O. Diekmann, J. A. P. Heesterbeek, and M. G. Roberts. The construction of next-generation matrices for compartmental epidemic models. *Journal of the Royal Society Interface*, 7:873–885, 2010. doi: 10.1098/rsif.2009.0386.

Arnaud Doucet and Adam M. Johansen. A Tutorial on Particle Filtering and Smoothing: Fifteen years later. In Dan Crisan and Boris Rozovskii, editors, *The Oxford Handbook of Nonlinear Filtering*. Oxford University Press, 2011.

Dirk Eddelbuettel and Romain François. Rcpp: Seamless R and C++ integration. *Journal of Statistical Software*, 40(8):1–18, 2011. doi: 10.18637/jss.v040.i08.

Dirk Eddelbuettel and Conrad Sanderson. RcppArmadillo: Accelerating R with high-performance C++ linear algebra. *Computational Statistics and Data Analysis*, 71:1054–1063, March 2014. doi: 10.1016/j.csda.2013.02.005.

W. R. Gilks, S. Richardson, and D. J. Spiegelhalter, editors. *Markov Chain Monte Carlo In Practice*. Chapman and Hall, 1996.

Walter R. Gilks and Carlo Berzuini. Following a moving target—Monte Carlo inference for dynamic Bayesian models. *Journal of the Royal Statistical Society. Series B (Methodological)*, 63(1):127–146, 2001. doi: 10.1111/1467-9868.00280.

N. J. Gordon, D. J. Salmond, and A. F. M. Smith. Novel approach to nonlinear/non-Gaussian Bayesian state estimation. *Radar and Signal Processing, IEE Proceedings F.*, 140(2):107–113, 1993. doi: 10.1049/ip-f-2.1993.0015.

Garrett Grolemund and Hadley Wickham. Dates and times made easy with lubridate. *Journal of Statistical Software*, 40(3):1–25, 2011. URL `https://www.jstatsoft.org/v40/i03/`.

Andrew Iskauskas, Ian Vernon, Michael Goldstein, Danny Scarponi, Nicky McCreesh, Trevelyan J. McKinley, and Richard G. White. Emulation and history matching using the `hmer` package. *Journal of Statistical Software*, 109(10):1–48, 2024.

Christopher I. Jarvis, Kevin Van Zandvoort, Amy Gimma, Kiesha Prem, CMMID COVID-19 working group, Petra Klepac, G. James Rubin, and W. John Edmunds. Quantifying the impact of physical distance measures on the transmission of COVID-19 in the UK. *BMC Medicine*, 18(124), 2020. doi: 10.1186/s12916-020-01597-8.

Olaf Mersmann, Heike Trautmann, Detlef Steuer, and Björn Bornkamp. *truncnorm: Truncated Normal Distribution*, 2023. URL `https://github.com/olafmersmann/truncnorm`. R package version 1.0-9, https://github.com/olafmersmann/truncnorm.

Deyu Ming and Daniel Williamson. *dgpsi: An R package powered by Python for modelling linked deep Gaussian processes*, 2024. URL `https://CRAN.R-project.org/package=dgpsi`. R package version 2.4.0.

Joël Mossong, Niel Hens, Mark Jit, Philippe Beutels, Kari Auranen, Rafael Mikolajczyk, Marco Massari, Stefania Salmaso, Gianpaolo Scalia Tomba, Jacco Wallinga, Janneke Heijne, Malgorzata Sadkowska-Todys, Magdalena Rosinska, and W. John Edmunds. Social contacts and mixing patterns relevant to the spread of infectious diseases. *PLoS Medicine*, 5(3):e74, 2008. doi: 10.1371/journal.pmed.0050074.

Douglas Nychka, Reinhard Furrer, John Paige, and Stephan Sain. fields: Tools for spatial data, 2021. URL `https://github.com/dnychka/fieldsRPackage`. R package version 16.3.

Edzer Pebesma. Simple Features for R: Standardized Support for Spatial Vector Data. *The R Journal*, 10(1): 439–446, 2018. doi: 10.32614/RJ-2018-009. URL `https://doi.org/10.32614/RJ-2018-009`.

Thomas Lin Pedersen. *patchwork: The Composer of Plots*, 2022. URL `https://CRAN.R-project.org/package=patchwork`. R package version 1.1.2.

Tony Plate and Richard Heiberger. *abind: Combine Multidimensional Arrays*, 2024. URL `https://cran.r-project.org/web/packages/abind/index.html`. R package version 1.4-8.

Christopher G. Prener and Charles K. Revord. areal: An R package for areal weighted interpolation. *Journal of Open Source Software*, 4(37), 2019. doi: 10.21105/joss.01221. URL `https://doi.org/10.21105/joss.01221`.

R Core Team. *R: A Language and Environment for Statistical Computing*. R Foundation for Statistical Computing, Vienna, Austria, 2022. URL `https://www.R-project.org/`.

Barret Schloerke, Di Cook, Joseph Larmarange, Francois Briatte, Moritz Marbach, Edwin Thoen, Amos Elberg, and Jason Crowley. *GGally: Extension to 'ggplot2'*, 2024. URL `https://ggobi.github.io/ggally/`. R package version 2.2.1, https://github.com/ggobi/ggally.

Luca Scrucca, Chris Fraley, T. Brendan Murphy, and Adrian E. Raftery. *Model-Based Clustering, Classification, and Density Estimation Using mclust in R*. Chapman and Hall/CRC, 2023. ISBN 978-1032234953. doi: 10.1201/9781003277965. URL `https://mclust-org.github.io/book/`.

P. van den Driessche and J. Watmough. Reproduction numbers and sub-threshold endemic equilibria for compartmental models of disease transmission. *Mathematical Biosciences*, 180:29e48, 2002. doi: 10.1016/S0025-5564(02)00108-6.

Pauline van den Driessche. Reproduction numbers of infectious disease models. *Infectious Disease Modelling*, 2 (3):288–303, 2017. doi: 10.1016/j.idm.2017.06.002.

W. N. Venables and B. D. Ripley. *Modern Applied Statistics with S*. Springer, New York, fourth edition, 2002. URL `https://www.stats.ox.ac.uk/pub/MASS4/`. ISBN 0-387-95457-0.

Hadley Wickham, Mara Averick, Jennifer Bryan, Winston Chang, Lucy D'Agostino McGowan, Romain François, Garrett Grolemund, Alex Hayes, Lionel Henry, Jim Hester, Max Kuhn, Thomas Lin Pedersen, Evan Miller, Stephan Milton Bache, Kirill Müller, Jeroen Ooms, David Robinson, Dana Paige Seidel, Vitalie Spinu, Kohske Takahashi, Davis Vaughan, Claus Wilke, Kara Woo, and Hiroaki Yutani. Welcome to the tidyverse. *Journal of Open Source Software*, 4(43):1686, 2019. doi: 10.21105/joss.01686.

Daniel B. Williamson, Trevelyan J. McKinley, Xiaoyu Xiong, James M. Salter, Robert Challen, Leon Danon, Ben Youngman, and Doug McNeall. On real-time calibrated prediction for complex model-based decision support in pandemics: Part 1. *https://www.medrxiv.org/content/10.1101/2025.05.16.25327688v1*, 2025.
